# Coordinated transcriptional and epigenetic regulation of type 2 CD8^+^ T cells in asthma

**DOI:** 10.3389/fimmu.2026.1837973

**Published:** 2026-10-08

**Authors:** Michaela Schedel, Sonia M. Leach, Laura Harmacek, Thomas Danhorn, Yi Jia, Victoria Meyboom, Fangkun Ning, Katsuyuki Takeda, Sven Michel, Brian P. O’Connor, Erwin W. Gelfand

**Affiliations:** 1 Department of Pulmonary Medicine, University Medical Center Essen – Ruhrlandklinik, Essen, Germany; 2 Department of Pulmonary Medicine, University Medical Center Essen, Essen, Germany; 3 Division of Cell Biology, Department of Pediatrics, National Jewish Health, Denver, CO, United States; 4 Department of Immunology and Genomic Medicine, National Jewish Health, Denver, CO, United States; 5 Department of Microbiology and Immunology, Geisel School of Medicine, Dartmouth College, Lebanon, NH, United States; 6 Center for Genes, Environment & Health, National Jewish Health, Denver, CO, United States; 7 Lipedema Foundation, New York, NY, United States; 8 University of Colorado Cancer Center and Department of Biomedical Informatics, University of Colorado Anschutz, Aurora, CO, United States; 9 Department of Pediatric Pneumology and Allergy, University Children’s Hospital Regensburg (KUNO), Regensburg, Germany

**Keywords:** epigenetic regulation, histone acetylation, severe steroid-resistant asthma, Tc2 CD8+ T cells, transcriptional networks

## Abstract

**Rational:**

Steroid-insensitive type 2 cytokine-producing CD8^+^ T cells (Tc2) are enriched in patients with severe asthma and correlate with disease severity, highlighting their potential role as drivers of treatment-refractory disease. The molecular mechanisms governing their functional differentiation remain incompletely understood.

**Methods:**

Mouse CD8^+^ T cells were differentiated *in vitro* under type 1 or type 2 polarizing conditions. Genome-wide transcriptional and H3 histone acetylation profiles were analyzed using RNA sequencing and chromatin immunoprecipitation (ChIP) sequencing. Both were integrated to identify H3Ac-associated transcriptional programs. Functional consequences of pharmacologic inhibition of histone acetyltransferase (HAT) activity in Tc2 cells were assessed after adoptive transfer into allergen-sensitized CD8-deficient mice. In parallel, transcriptomic profiling of human CD8^+^ T cells from healthy individuals and steroid-resistant asthmatics was performed and integrated with the mouse Tc2 dataset to identify conserved regulatory pathways.

**Results:**

Tc1 and Tc2 differentiation were characterized by coordinated transcriptional remodeling with distinct type 2-associated networks emerging in response to IL-4 and T cell receptor stimulation including *Il-24*, *Cyp11a1*, and *Il-13*. Integration of histone acetylation data and transcriptional networks identified condition-specific H3Ac-associated differentially-expressed genes (DEGs) and functional programs specific to Tc1 and Tc2 cells. While H3Ac-associated DEGs in Tc1 cells were linked predominantly to broader inflammatory and immune effector pathways, this integrative approach showed an enrichment of type 2 immune pathways in Tc2 cells. Pharmacologic modulation of HAT activity increased Tc2 cytokine responses and enhanced the pathogenic effects of Tc2 cells *in vivo*, resulting in heightened airway hyperresponsiveness and eosinophilic inflammation. Human CD8^+^ T cells from steroid-resistant asthmatics exhibited distinct transcriptional profiles compared with healthy individuals, including increased *IL-13* expression. Cross-species integration revealed significant overlap between mouse Tc2 transcriptional signatures and gene networks in steroid-resistant asthmatics.

**Conclusion:**

These findings propose coordinated epigenetic and transcriptional remodeling as a central mechanism regulating functional differentiation of Tc2 cells. They further define shared molecular pathways linking CD8^+^ T cell programming to type 2 inflammation in severe asthma, providing insight into mechanisms that may contribute to persistent disease and therapeutic resistance.

## Graphical Abstract

**Figure:**
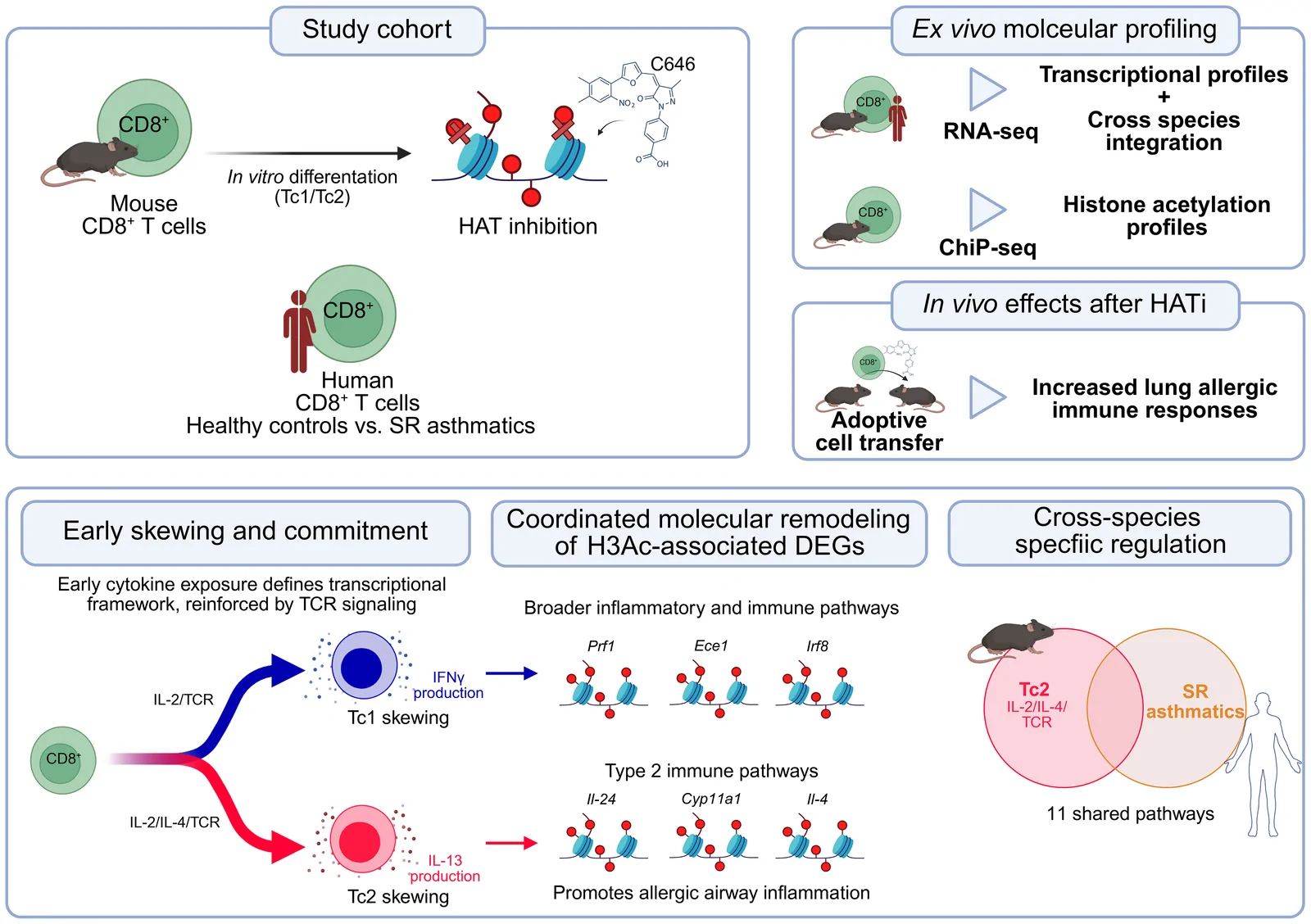

## 1 Introduction

Asthma is a heterogeneous inflammatory respiratory disease in which type 2 immune responses drive eosinophilia, airway hyperresponsiveness, and disease severity (1). Although CD4^+^ Th2 cells and type 2 innate lymphoid cells (ILC2s) are established mediators of these type 2 responses (2, 3), accumulating evidence identifies CD8^+^ T cells as important contributors to type 2 allergic airway disease, particularly in severe, corticosteroid-resistant (SR) asthma (4–10).

CD8^+^ T cells typically differentiate into cytotoxic IFNγ-producing effector cells upon activation (11–14). However, in type 2 inflammatory environments containing IL-4 and IL-33, CD8^+^ T cells acquire a type 2 cytokine-producing phenotype, termed Tc2 cells (6, 7, 10). In patients with severe asthma, Tc2 cells are increased in the blood and airways (8, 15), and produce IL-5 and IL-13, key mediators of type 2 and eosinophilic inflammation. Notably, Tc2 cells have been linked to corticosteroid resistance (10, 16), a hallmark of severe asthma. In addition to IL-4, the alarmin IL-33 promotes Tc2 differentiation (10). Despite this emerging importance and relevance in severe asthma, the molecular mechanisms regulating Tc2 lineage commitment and pathogenic function remain poorly understood and require definition if we are to improve asthma outcomes.

Differentiation of CD8^+^ T cells into distinct functional lineages is proposed to require coordinated transcriptional and epigenetic regulation. Prior studies demonstrated that lineage commitment is associated with epigenetic regulation at key transcription factor loci, including *Tbx21* and *Gata3* (17). Histone modifications regulate chromatin accessibility and transcriptional activity (18), stabilizing lineage-specific gene expression programs. However, the global epigenetic and transcriptional mechanisms that govern Tc2 differentiation, and how these processes are integrated during lineage commitment, need to be further defined.

Histone acetylation, mediated by histone acetyltransferases, promotes chromatin accessibility and transcriptional activity (19). Dysregulated histone acetylation has been implicated in inflammatory diseases such as asthma (20, 21), but a role in directing CD8^+^ T cell lineage specification and Tc2 differentiation is unknown. In particular, it is unclear if histone acetylation influences transcriptional regulation that promote Tc2 differentiation and pro-allergic effector function. We hypothesized that histone acetylation regulates lineage-specific transcriptional programs controlling Tc2 differentiation and pathogenic effector functions.

To address this, we performed integrated transcriptomic and epigenetic profiling of mouse CD8^+^ T cells during Tc1 and Tc2 differentiation. In an *in vivo* adoptive transfer model of allergic airway inflammation, we tested the functional role of histone acetylation using pharmacologic inhibition. Global expression patterns in CD8^+^ T cells from SR asthmatics were examined in an exploratory approach. These studies provided the first integrated analyses of histone acetylation and transcriptional remodeling, supporting a mechanistic framework linking epigenetic regulation to pathogenic CD8^+^ T cell differentiation in asthma.

## 2 Materials and methods

### 2.1 Animals

Pathogen-free, 8- to 10-week-old OT-1 mice expressing a T cell receptor (TCR) specific for the SIINFEKL peptide [(OVA_257-264_)] and homozygous CD8-deficient mice (6, 7, 18) were bred in the animal facility at National Jewish Health (Denver, CO, #AS2483).

### 2.2 Mouse CD8^+^ T cell culture and flow cytometry

CD8^+^ effector memory T cells were generated *in vitro* as previously described (see **Supplementary Figure 1**) (6, 7, 18). Only female OT-I mice were used as donors for CD8^+^ T-cell differentiation based on consistently higher differentiation efficiency and reproducibility, minimizing variability arising from sex-dependent intrinsic differences in donor CD8^+^ T cells. Using a single donor sex allowed the exclusion of a potential confounder. Briefly, mononuclear cells were isolated from the spleens of female OT-1 mice and stimulated with 1 µg/ml SIINFEKL peptide for 1.5 hours. After 2 days, living cells were isolated using histopaque (Sigma-Aldrich, St. Louis, MO) and cultured in recombinant mouse IL-2 (20 ng/ml, R&D, Minneapolis, MN) or IL-2/IL-4 (20 ng/ml, Peprotech, Rocky Hill, NJ). For some experiments, C646 (10 nM, p300 inhibitor, Sigma Aldrich) was added. The medium supplemented with IL-2 or IL-2/IL-4 was changed daily for 4 consecutive days. Cells were then re-stimulated with 1 µg/ml SIINFEKL and 2 µM monensin (Calbiochem, La Jolla, CA) for 4 hours. Intracellular staining of mouse IFNγ^+^ and IL-13^+^ CD8^+^ T cells was performed as previously described (6, 7, 18) using an eFluor 660-labeled anti-mouse IFNγ (#50-7311-82, eBioscience, San Diego, CA) and PE-labeled anti-mouse IL-13 (#17-7311-82, eBioscience) antibody. Cell staining was monitored on a FACSCalibur (BD Bioscience, Franklin Lakes, NJ) and analyzed using Flowjo software (Tree Star, Inc, Ashland, OR).

### 2.3 Secondary allergen challenge model and functional characterization of bronchoalveolar lavage

While donor cells were derived exclusively from female OT-I mice, both male and female CD8-deficient recipient mice were used in adoptive transfer experiments to assess the *in vivo* effects of adoptively transferred CD8^+^ T cells across sexes. The experimental protocol for secondary sensitization and challenge to OVA using CD8-deficient mice was performed as described previously (6, 7, 18). The number of mice per sex was comparable between experimental groups. To address the effect of C646 on CD8^+^ T cell-mediated airway hyperresponsiveness (AHR), CD8^+^ T cells (0.75x10^6^ cells) generated in medium containing IL-2/IL-4 without or with C646 (10 nM) were adoptively transferred into CD8-deficient mice intravenously prior to the secondary OVA challenge on day 44. Two hours after cell transfer, sensitized mice were re-challenged (secondary) with 1% OVA for 20 minutes by nebulization. Airway responsiveness to inhaled methacholine was measured and bronchoalveolar lavage (BAL) samples were collected 24 hours after the secondary challenge as described previously (6, 7, 18). Cytospin slides of leukocytes from the BAL fluid were stained with Leukostat (Fisher Scientific, Schwerte, Germany) and differentiated by standard hematological procedures in a blinded fashion (6, 7, 18). At least 200 cells were counted and identified as macrophages, lymphocytes, neutrophils, and eosinophils based on histological properties. The total count for each cell type was calculated based on relative cell counts and the initially determined total cell number. Statistical analyses of outcomes of OT-1 mice in response to the secondary allergen challenge model were conducted using the GraphPad Prism Software (GraphPad Company, Boston, MA). Changes in AHR and differential BAL cell count were assessed using 2-way analysis of variance (ANOVA) followed by Tukey-Kramer *post-hoc* tests for all pairwise comparisons and corrected for multiple testing. All data were normally distributed. A P-value less than 0.05 was considered statistically significant. Results were expressed as mean ± standard deviation. *In vivo* data were obtained from 2 independent experiments (5–9 mice per group).

### 2.4 Isolation and culturing of human CD8^+^ T cells

Patients with a diagnosis of SR asthma [n = 3, female and male, forced expiratory volume in 1 second (FEV1) ≤ 80%)] according to American Thoracic Society criteria were selected (16). SR asthmatics were characterized at baseline and received oral prednisolone (40 mg for 7 days). After prednisolone treatment, SR asthmatics showed less than 10% improvement in FEV1. Healthy controls (n = 2) had no history of atopic or respiratory disease. Ethics approval for this study was obtained by the Institutional Review Board of National Jewish Health (Denver, Colorado, #HS-2409-NJH). Sex was not analyzed as a biological variable.

Peripheral blood mononuclear cells were isolated from heparinized venous blood, followed by CD8^+^ T cell isolation by negative selection using the magnetic bead human CD8^+^ T cell isolation kit II (Miltenyi Biotec, Auburn, California) (16). Isolated CD8^+^ T cells (1x10^6^ cells/ml, > 95% purity) were stimulated with anti-human CD3/CD28 (2 mg/ml each; BD Pharmingen, San Jose, CA) and human recombinant IL-2 (100 U/ml; Peprotech, Cranbury, NJ) for 2 days. Cells were then transferred onto a new plate. The medium containing IL-2 was changed every other day and cells were harvested after 6 days in culture. Total RNA from CD8^+^ T cells (1x10^6^ cells) was isolated using the NucleoSpin RNA isolation kit (Macherey Nagel, Düren, Germany) following the manufacturer’s protocol.

### 2.5 Next-generation RNA-seq and ingenuity pathway analysis^®^

RNA libraries were prepared from 200 ng total RNA using the RNA Library Preparation Kit (Kapa Biosystems #KK8420, Woburn MA) and primers modified for Ion Torrent sequencing (unstranded, single-end). Libraries were analyzed for size and quality using the High Sensitivity Bioanalyzer Kit (Agilent #5067-4626, Santa Clara, CA) and sequenced with the Life Technologies Ion Proton. Between 10 and 21 million reads were generated for each RNA-seq library.

Base calling, quality filtering, and adapter trimming of sequence reads were performed with the Torrent Suite software (Life Technologies/Thermo Fisher, version 5.0.3) using default parameters. Reads were aligned to the mm9 (mouse) and hg19 (human) reference genomes using the STAR aligner (version 2.5.1b) (22) with splice site data from the Ensembl annotation (mouse: version 67; human: version 75). Reads mapping unambiguously to each gene in the Ensembl annotation were quantified using the featureCounts program from the subread package (version 1.5.0-p1). Statistical comparisons were performed with the DESeq2 package (version 1.811) (23) in R (version 3.2.0) (24) using the default built-in independent filtering and linear models that took into account any effects of the specific replicate (mouse) on gene expression, in addition to the variable of interest. A factor for replicate was included in the DESeq2 model to account for batch effects. Differentially expressed genes (DEGs) were defined using a threshold of fold change > |1.5| (=log2 fold change ± 0.58) and P_adjusted_ < 0.05. Heatmaps and unsupervised hierarchical clustering were generated using the heatmap in R (version 3.2.0). Principal component analysis (PCA) was performed using the prcomp function in R (version 3.2.0).

DEGs identified by DESeq2 were subjected to pathway enrichment analyses using the Ingenuity Pathway Analysis^®^ (IPA^®^, Qiagen, Hilden, Germany) tool. For both mouse and human datasets, genes meeting the significance criteria of a fold change > |1.5| and an adjusted P value (P_adjusted_ < 0.05) were imported into IPA^®^. Core analyses were performed using default settings to identify significantly enriched canonical pathways. For the mouse dataset, pathways with a Benjamini–Hochberg (BH)-adjusted enrichment P < 0.05 identified in IPA^®^ were considered significant. Due to the exploratory nature and limited sample size of the human SR asthma cohort, human pathway enrichment analyses were interpreted additionally using a nominal enrichment P < 0.05 to enable identification of biologically relevant immune-associated pathways despite the reduced statistical power. BH-adjusted values were additionally provided. To enable biologically focused comparison, additional analyses concentrated on pathways annotated under immune-related functional categories, including “cellular immune responses”.

### 2.6 Chromatin immunoprecipitation and next-generation sequencing

Genome-wide ChIP assays for H3Ac were performed on three biological replicates using CD8^+^ T cells (5x10^6^) cultured with IL-2 or IL-2/IL-4 (± re-stimulation with SIINFEKL) adapted from the manufacturer’s protocol using the ChIP-It Express kit (Active Motif, Carlsbad, CA). Cells were fixed with 1% EM-grade formaldehyde (Thermo Fisher, Waltham, MA), lysed, and chromatin sheared using the isothermal Covaris S2 (Woburn, MA) to obtain 100–300 bp fragments. DNA-protein complexes were immunoprecipitated with the Pan-acetyl H3 antibody (Active Motif), captured by magnetic beads, cross-links reversed, and the genomic DNA purified (Invitrogen Magnify ChIP Kit). The isolated DNA was processed for next-generation library construction using a modified protocol and the Ion Plus Fragment Library Kit #4471252. Briefly, library construction was completed starting with end repair of the ChIP DNA, followed by adaptor ligation, emulsion PCR, and finally size selection purity. Libraries were analyzed for size and quantity using the High Sensitivity Bioanalyzer Kit (Agilent Technologies #5067-4626). Libraries were sequenced (unstranded, single-end) on an Ion Proton (Life Technologies), generating 9–20 million reads for each ChIP and input sample. Total input control libraries were used to compare peaks in the ChIP samples.

### 2.7 Computational analyses of ChIP-seq data

ChIP-seq sequencing reads were aligned to the mouse reference genome (mm9) using Ion Torrent Suite software. Peak calling was performed using the MACS2 software (25), comparing acetylated histone H3 IPs to their respective total input controls. Peak significance was determined with a false discovery rate (FDR) cutoff of 0.001. Consensus peaks were identified across all narrowPeaks files using DiffBind (version 3.20.0) (26), and affinity matrices were established with minOverlap=2, summits=FALSE, bUseSummarizeOverlaps=TRUE and DBA_NORM_NATIVE for normalization. Differential peak binding analyses by DiffBind used DESeq2 with replicate and sample group as model parameters, and included all 12 samples as input. Consensus peaks were associated with the nearest gene based on genomic location using HOMER (version 5.1) with Ensembl 67 annotations restricted to protein coding genes. The ChIP- and RNA-seq data were integrated for each pairwise sample group comparison as follows. The H3Ac consensus peaks for a given pairwise comparison were first filtered to those peaks called in at least two of the six samples relevant to the comparison, and found within ±1,000 bp of a transcription start site (TSS) of DEGs specific for the comparison. The FDR was then recomputed among this filtered peak set using the DESeq2 nominal P values using the p.adjust function, with a significance threshold of Padjusted < 0.10. DEGs associated with at least one differential peak were used for IPA^®^ pathway analyses. If indicated, pathways were restricted to “cellular immune response” pathways only. Integrative heatmap visualization of the H3Ac affinity matrix of normalized read counts and RNA-seq log2-normalized transcript per million (TPM) counts used the ComplexHeatmap package (version 2.25.1).

### 2.8 Cross-species integration analyses of human and mouse RNA-seq data

For cross-species integrative analysis, DEGs were first identified independently for human CD8^+^ T cells (healthy controls vs. SR asthmatics) and mouse CD8^+^ T cells (Tc2 vs. IL-2) with a threshold of fold change > |1.5| and P_adjusted_ < 0.05. Using this prior gene-level FDR filtering, DEGs from each dataset were analyzed using IPA^®^. Pathways annotated under the category “cellular immune response” were preselected. A significance threshold of a nominal P < 0.05 was applied human pathways and BH P < 0.05 for mouse IPA^®^ enrichment output reporting. Cross-species comparisons were performed by identifying overlapping and distinct cellular immune response pathways in human and mouse datasets.

### 2.9 Data availability

RNA-seq data for mouse CD8^+^ T cells are available under GEO SuperSeries accession #GSE109840, and for human CD8^+^ T cells under GEO accession SuperSeries #GSE100624. ChIP-seq data are available at GEO SuperSeries accession #GSE109156.

## 3 Results

### 3.1 Distinct transcriptomic profiles of mouse CD8^+^ T cells during Tc1 or Tc2 conversion

The differentiation of naïve CD8**^+^** T cells from OT-1 mice into Tc1 (IFNγ^+^) or Tc2 (IL-13^+^) cells was examined under defined *in vitro* conditions. CD8^+^ T cells were cultured for 4 days with either IL-2 alone or IL-2/IL-4 (see **Supplementary Figure 1**) (6, 7, 18). Following T cell receptor (TCR) re-stimulation with the T cell-stimulating ovalbumin (OVA) epitope SIINFEKL, IL-2-treated cells primarily differentiated into IFNγ-producing Tc1 cells, whereas IL-2/IL-4-treated cells following SIINFEKL re-stimulation transitioned into IL-13-producing Tc2 cells (6, 7, 18). Visualization of global patterns of variation in complex datasets by PCA revealed condition-dependent clustering of samples with baseline (IL-2 alone), IL-2/IL-4-treated, and cytokine-expressing Tc1 (IL-2/TCR) or Tc2 (IL-2/IL-4/TCR) cells separating along the major axes of variance (**Figure 1A**). The largest differences in gene expression occurred between CD8**^+^** T cells exposed to IL-2 alone and TCR re-stimulated CD8^+^ T cells, indicating transcriptional reprogramming following TCR activation. Tc1 cells showed the strongest separation relative to the other conditions. Hierarchical clustering supported these findings (see **Supplementary Figure 2**).

**Figure 1:**
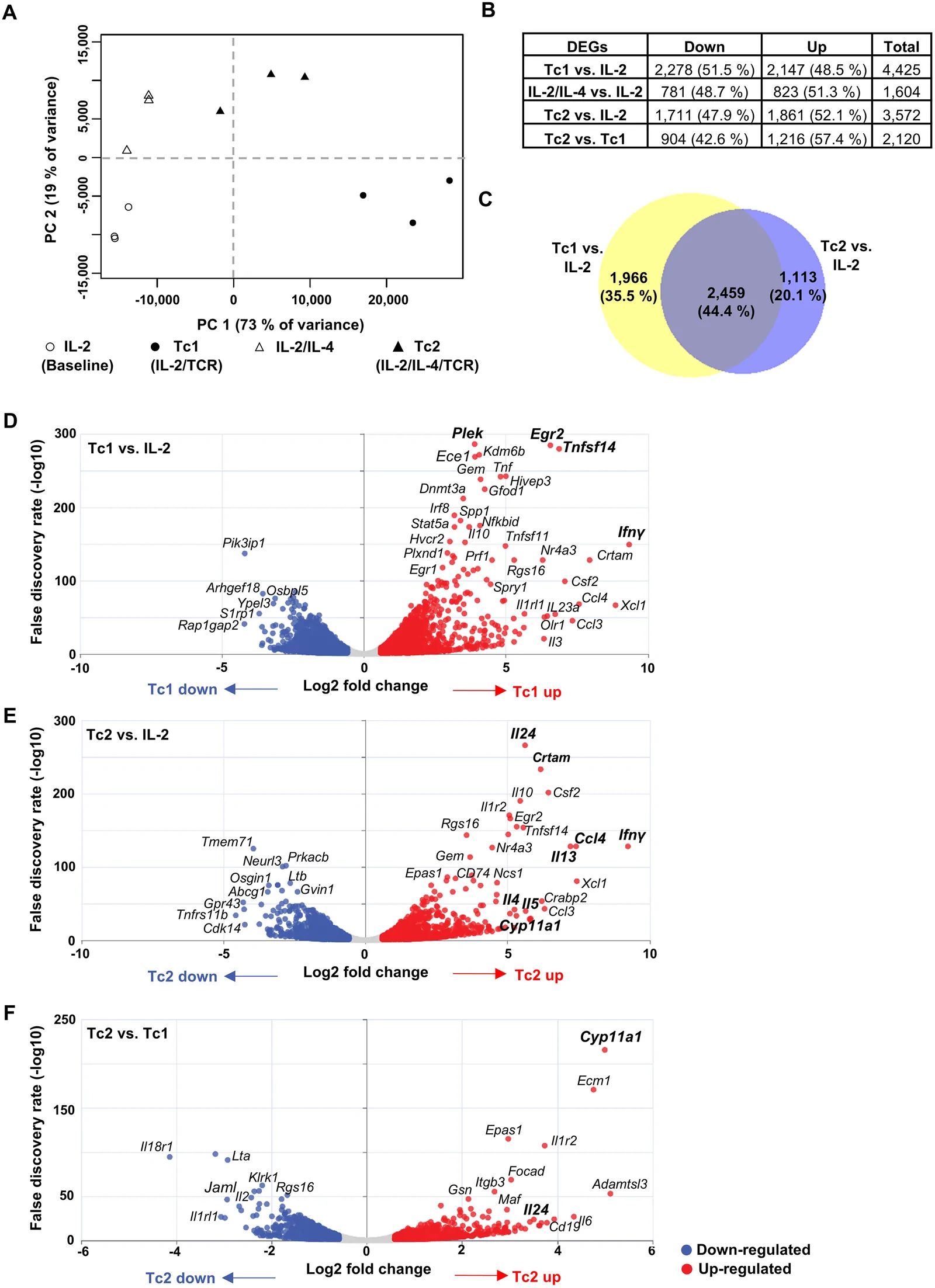
RNA-seq profiles are distinct in a stepwise differentiation of mouse CD8^+^ T cells. **(A)** Principal Component Analysis (PCA) plot. **(B)** Global changes in gene expression in response to TCR re-stimulation (SIINFEKL) with or without exposure to IL-4 (fold change > |1.5|, P_adjusted_ < 0.05). **(C)** Venn diagram for overlapping and distinct gene signatures in IFNγ-producing (Tc1) vs. IL-13-producing (Tc2) CD8^+^ T cells (fold change > |1.5|, P_adjusted_ < 0.05) compared to its baseline (IL-2 alone). Volcano plots showing differentially-expressed genes (DEGs) in CD8^+^ T cells from **(D)** Tc1 cells compared to cells cultured in IL-2 alone, **(E)** Tc2 cells compared to cells cultured in IL-2 alone, and **(F)** Tc2 cells compared to Tc1 cells. The x-axis represents log2 fold changes and the y-axis the –log10 P_adjusted_-values. Red circles indicate up-regulated DEGs and blue circles show down-regulated DEGs with a fold change > |1.5| (=log2 fold change ±0.58) and a P_adjusted_ < 0.05. Selected gene names are annotated. Results are depicted from 3 independent experiments using 1 mouse spleen per experiment to differentiate CD8^+^ T cells into Tc1 or Tc2 cells. TCR = T cell receptor.

Analyses of differentially-expressed genes (DEGs) at each stage showed no overall bias toward up- or down-regulation, while DEG numbers markedly increased after TCR re-stimulation (**Figure 1B**). Comparing DEGs of Tc1 and Tc2 (each compared to IL-2 baseline) revealed both shared and condition-specific transcriptional patterns (**Figure 1C**). Approximately 45% of DEGs (n = 2,459) were common to both Tc1 and Tc2 cells, consistent with a shared TCR-driven activation program. In contrast, 35% (n = 1,966) and 20% (n = 1,113) of DEGs were unique to Tc1 and Tc2 cells, respectively, supporting condition-specific transcriptional programs.

Volcano plot analyses (**Figures 1D–F**) illustrated the distribution of DEGs across conditions, capturing both the magnitude and statistical significance of transcriptional changes underlying Tc1 and Tc2 differentiation. Consistent with these findings, heatmaps of the top up- and down-regulated DEGs identified in the direct Tc1 vs. Tc2 comparison further demonstrated distinct but partially overlapping transcriptional regulation across all differentiation conditions (see **Supplementary Figure 3**). Comparison of Tc1 cells with IL-2 treated cells revealed prominent differential expression of *Plek*, *Egr2*, *Tnfsf14*, and *Ifnγ*, reflecting transcriptional changes associated with TCR re-stimulation in the absence of IL-4 (**Figure 1D**). In contrast, Tc2 cells showed the strongest differential expression profile for *Il-24*, *Crtam*, and *Ccl4* when compared with IL-2 treated cells (**Figure 1E**). Consistent with acquisition of a type 2-associated transcriptional program, *Il-13*, *Il-4*, *IL-5*, and *Cyp11a1* were highly differentially expressed. Notably, *Ifnγ* was also among the most significant DEGs in Tc2 cells, indicating that TCR-induced activation-associated transcriptional responses were not completely restricted to the respective lineage-specific program. Direct comparison of Tc2 and Tc1 cells further revealed distinct lineage-associated transcriptional profiles, with *Cyp11a1* representing the most prominent Tc2-associated DEG (**Figure 1F**). Examination of key lineage-defining genes further confirmed that early cytokine exposure (IL-2 vs. IL-2/IL-4) establishes a transcriptional framework prior to full lineage commitment, which is then reinforced by TCR signaling, thereby biasing differentiation towards IFNγ-producing Tc1 or IL-13-producing Tc2 cells (see **Supplementary Figures 4A, B**). Together, these findings demonstrate that TCR re-stimulation induces distinct transcriptional programs depending on the cytokine environment, while retaining a degree of overlap in activation-associated gene expression.

### 3.2 Coordinated Tc2 pathway activation drives Tc2 differentiation

IPA^®^ of DEGs revealed distinct pathway enrichment between Tc1 and Tc2 cells. Tc1 cells, compared to IL-2-treated cells without TCR re-stimulation were primarily associated with broad immune pathways related to their core functions, including antiviral responses, as well as non-immune pathways (**Figure 2A**, see **Supplementary Table 1**). In contrast, Tc2 cells showed stronger enrichment for canonical cellular immune response pathways, with top-ranked pathways directly related to type 2 cellular function (**Figure 2B**; see **Supplementary Table 2**). At the final Tc1 vs. Tc2 commitment stage, 482 pathways were shared between the two cell types, corresponding to an overlap of 65.5% across all canonical pathways, while 185 and 69 sets of pathways were uniquely enriched in Tc1 and Tc2 cells, respectively (**Figure 2D**; see **Supplementary Tables 1**, **2**). When restricting the analyses to cellular immune response pathways, the overlap increased to 80% (**Figure 2E**), indicating that both cell types engage shared immune signaling networks. Out of these, only 6 and 7 sets of pathways were uniquely enriched in Tc1 and Tc2 cells (**Figure 2E**; see **Supplementary Figures 5A, B**). However, the overall pathway ranking based on BH P_adjusted_ values revealed substantial differences in pathway prioritization between conditions (**Figure 2A, B**). In Tc1 cells compared to cells differentiated in IL-2, the highest-ranking cellular immune response pathway appeared only at rank 19 (cyclophilin signaling), with the top 10 immune pathways distributed between ranks 19 and 95 (**Figure 2A**; see **Supplementary Table 1**). In contrast, Tc2 cells showed enrichment of key type 2-associated pathways at higher ranks, including Th1 and Th2 activation (rank 4), Th2 pathway (rank 8), and IL-4/IL-13 signaling (rank 22, **Figure 2B**; see **Supplementary Table 2**).

**Figure 2:**
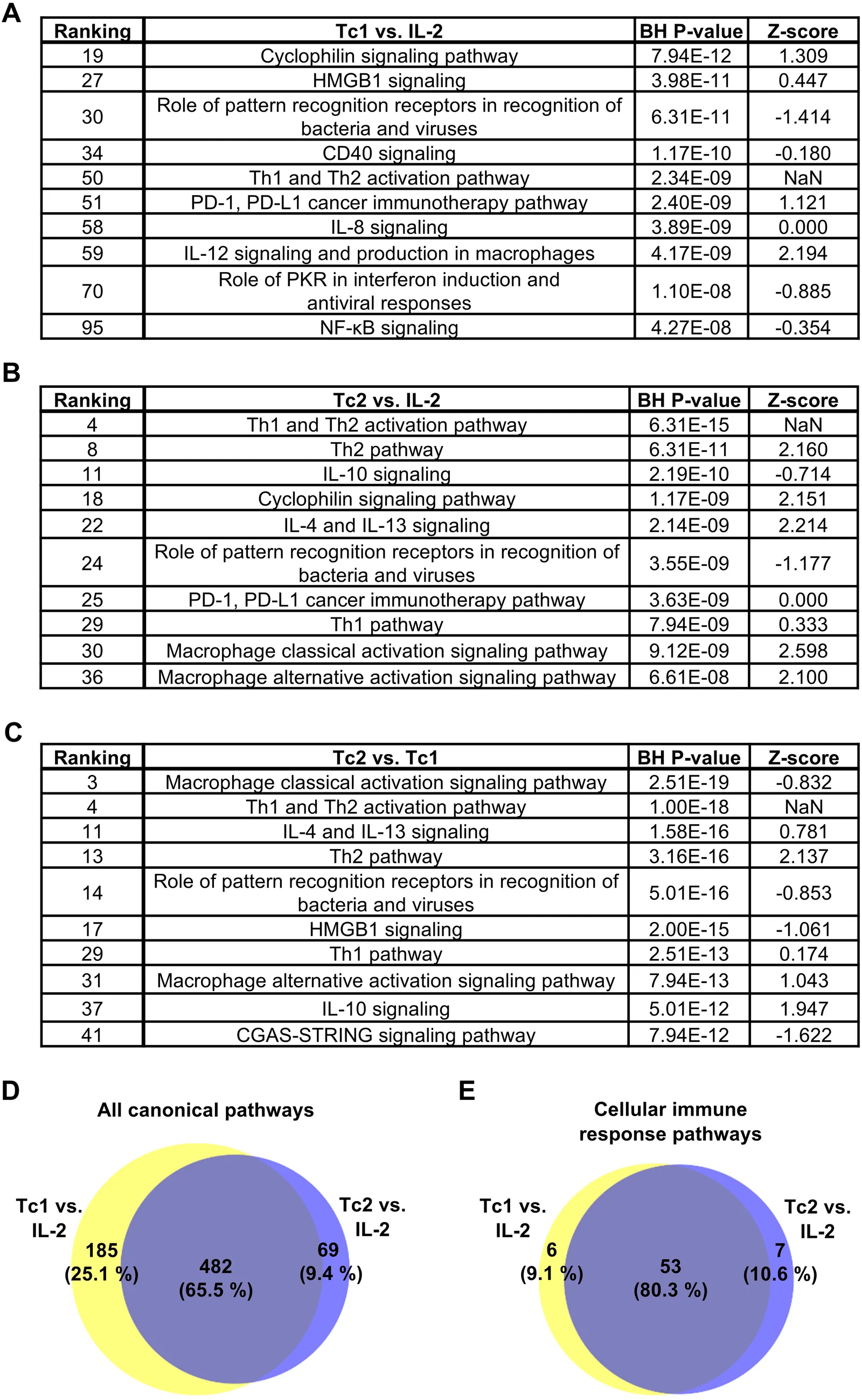
Differentially-expressed genes are enriched for shared and distinct pathways in Tc1 compared to Tc2 cells. Ingenuity Pathway Analysis^®^ (IPA^®^) was performed for differentially- expressed genes with a fold change > |1.5| and a P_adjusted_ < 0.05. Top immune pathways were selected and displayed based on their overall ranking in **(A)** Tc1 and **(B)** Tc2 cells compared to cells cultured in IL-2 alone, as well as **(C)** Tc2 cells compared to Tc1 cells. Benjamini Hochberg (BH) and z-scores are reported. Venn diagram of overlapping and distinct pathways derived from differentially-expressed genes in Tc1 vs. Tc2 after normalization to its baseline (IL-2 alone) for **(D)** all canonical pathways (BH P < 0.05) and **(E)** for cellular immune response pathways (BH P < 0.05). Results are depicted from 3 independent experiments using 1 mouse spleen per experiment to differentiate CD8^+^ T cells into Tc1 or Tc2 cells.

Direct pathway analysis of Tc2 vs. Tc1 DEGs identified Th1 and Th2 activation, IL-4/IL-13 signaling, and the Th2 pathway among the highest-ranking enriched canonical and cellular immune response pathways (**Figure 2C**; see **Supplementary Table 3**). These differences likely reflect fundamental distinctions in the biological roles of Tc1 and Tc2 cells. While Tc1 cells engage in broader transcriptional programs with diverse immune effector functions, Tc2 cells exhibit a more specialized transcriptional network aligned with type 2 immune functions.

### 3.3 Epigenetic remodeling influences Tc1 to Tc2 transition

Global histone acetylation (pan-H3 histone acetylation, H3Ac) was examined at defined stages during Tc1 and Tc2 differentiation to determine if transcriptional reprogramming was associated with epigenetic modification. Genomic distribution of H3Ac peaks identified by MACS2 were integrated across samples using Diffbind (26) and consensus peaks were further annotated using HOMER (27). Across differentiation stages, overall peak localization remained comparable, with the majority of H3Ac peaks mapping to promoter regions (73%, **Figure 3A**) and minimal variation in distance to transcription start sites (see **Supplementary Figure 6**), indicating stable genome-wide distribution patterns.

**Figure 3:**
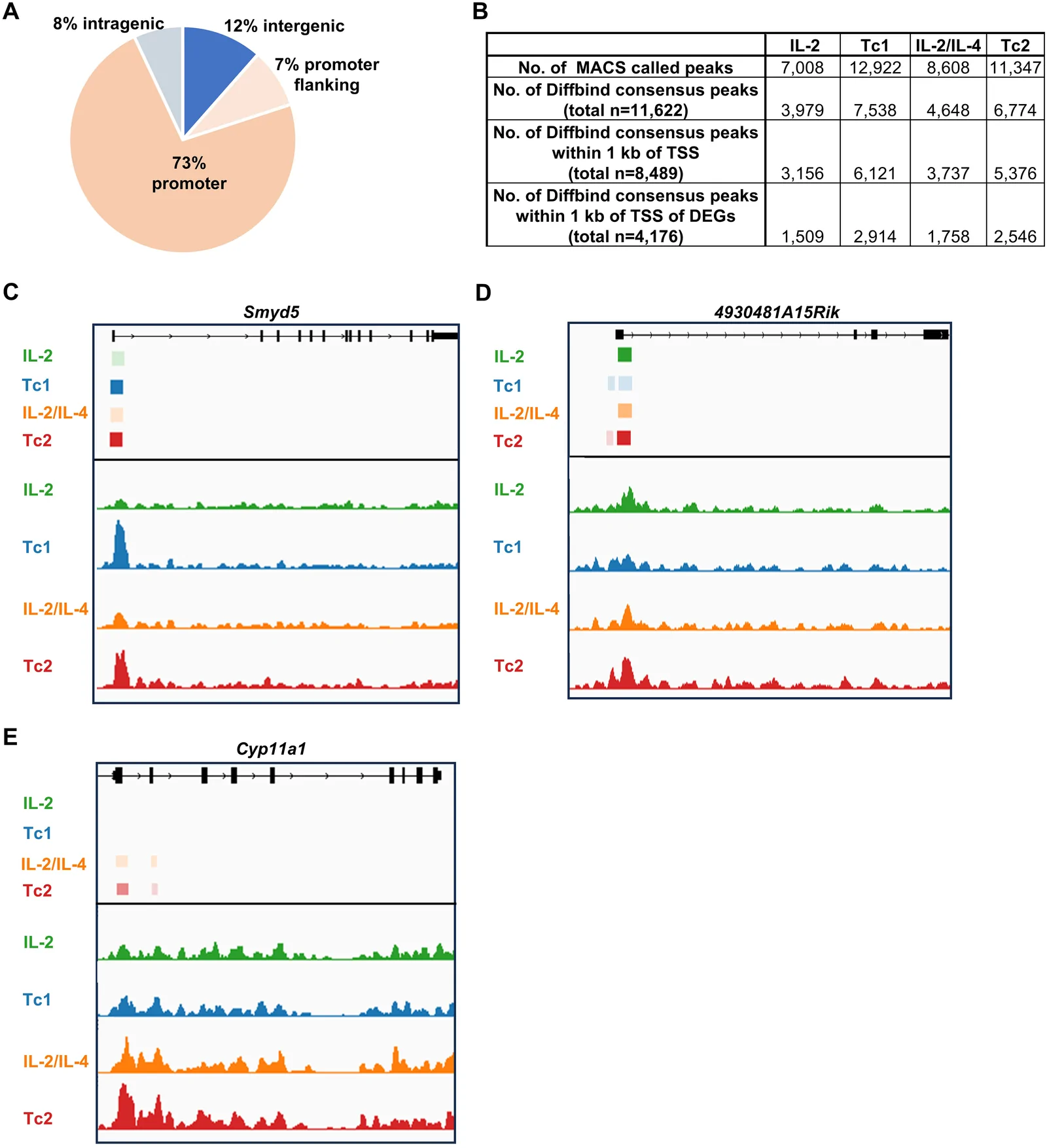
CD8^+^ T cells undergo epigenetic remodeling during differentiation to Tc1 or Tc2 cells. **(A)** Pie chart of the genetic loci distribution of 11,622 pan-H3 histone acetylation (H3Ac) consensus peaks are depicted as percentages. **(B)** Number of identified H3Ac peaks at each CD8^+^ T cell differentiation stage including number of MACS called peaks, number of DiffBind consensus peaks, number of DiffBind consensus peaks within ±1,000 bp of the transcription start site (TSS), and number of DiffBind consensus peaks within ±1,000 bp of differentially-expressed genes (DEGs). **(C–F)** ChIP-seq analyses of H3Ac at the most significant Tc1 and Tc2 regulator gene loci. H3Ac peaks were visualized using Integrated Genomic Viewer (IGV) in promoter regions for **(C)** *Smyd5*, **(D)** *4930481A15Rik*, and **(E)** *Cyp11a1.* CD8^+^ T cells were differentiated in IL-2 (green), IL-2/TCR (Tc1, blue), IL-2/IL-4 (orange), or IL-2/IL-4/TCR (Tc2, red). Upper panel: shown are both consensus peak calls in each group (shaded by number of replicates) and the read coverage (summed across replicate experiments). Shading of boxes represent number of replicates with a given called peak; the darkest shading represents peaks in all 3 independent experiments, whereas the lightest shading indicated peaks detected in a single experiment. Lower panel: sum of H3Ac peak wiggle tracks of fold enrichment over background. Results are depicted from 3 independent experiments using 1 mouse spleen per experiment to differentiate CD8^+^ T cells into Tc1 or Tc2 cells.

To further characterize the H3Ac landscape across the four differentiation conditions, the average number of identified and annotated peaks across replicates per condition are summarized in **Figure 3B** (see **Supplementary Table 4**). TCR re-stimulation markedly increased H3Ac levels in both IL-2- and IL-2/IL-4-treated cells, generating more than11,000 acetylation peaks genome-wide (**Figure 3B**), consistent with activation-induced chromatin remodeling. DiffBind identified a total of 11,622 consensus peaks across all conditions, of which 8,489 were located within ±1,000 bp of a TSS. Of these, 2,914 consensus peaks were associated with genes identified as differentially expressed in Tc1 cells, while 2,546 were detected in Tc2 cells, respectively. The corresponding numbers shown for each differentiation condition in each condition are not mutually exclusive, as individual consensus peaks may be detected across multiple differentiation states.

Examination of individual loci further illustrated condition-associated H3Ac remodeling. Altered H3Ac-associated genes in Tc1 cells compared to cells differentiated in IL-2 alone included *Smyd5* (**Figure 3C**; see **Supplementary Figure 7A**), *Adamts14*, and *Mettl2* ranking as the top candidates based on their log2 fold change value (log2FC > 1.56, P < 7.33E-07). *Smyd5*, which encodes a histone methyltransferase, was also the most highly altered H3Ac peak in Tc2 cells (log2FC = 1.16, P = 3.93E-07; **Figure 3C**; see **Supplementary Figure 7A**), followed by *Il-24* and *Cyp11a1* (log2FC > 1.08, P < 2.35E-06; **Figure 3E**; see **Supplementary Figure 7C**). *Il-4* also ranked among the top five H3Ac-associated genes in Tc2 cells (log2FC = 0.97, P = 1.52E-05). Notably, the differential H3Ac peaks were predominantly associated with increased acetylation. Only 4930481A15Rik (log2FC = -0.69, P = 0.01), a long noncoding RNA, showed a decrease in H3Ac in Tc1 compared with IL-2 treated cells (**Figure 3D**; see **Supplementary Figure 7B**). Thus, although some prominent H3Ac-associated genes were shared between differentiation states, the Tc2-associated H3Ac landscape was enriched for genes linked to type 2 immune responses, whereas the top Tc1-associated genes represented a broader range of cellular and immune regulatory functions.

### 3.4 TCR re-stimulation leads to coordinated H3Ac-related transcriptional changes in Tc1 and Tc2 cells

To assess the relationship between transcriptional changes and epigenetic remodeling during CD8^+^ T cell differentiation, we integrated RNA-seq-derived DEGs (P_adjusted_ < 0.05) with H3Ac ChIP-seq peaks (P_adjusted_ < 0.10) located within ±1,000 bp of the TSS. This genome-wide approach revealed stage-specific H3Ac-associated DEGs without preselection based on pathway membership. Compared with IL-2 treated cells, Tc1 differentiation was associated with 862 DEGs accompanied by increased H3Ac, whereas only one DEG showed decreased H3Ac (**Figures 4A, C**). In Tc2 cells, 172 DEGs were associated with increased H3Ac, with no DEGs showing decreased histone acetylation (**Figures 4A, D**). In contrast, no significant H3Ac changes were associated with DEGs in IL-2/IL-4-treated cells in the absence of TCR re-stimulation (**Figure 4A**; see **Supplementary Figure 8**). Scatter plot visualization of the H3Ac-associated DEGs further highlighted candidate genes associated within the two differentiation states (**Figures 4C, D**). In Tc1 cells compared to cells differentiated in IL-2 alone, *Ece1, Prf1*, and *Irf8* were among the most prominent DEGs regulated by histone acetylation (**Figure 4C**), whereas *Cyp11a1, Il-4*, and *Il-24* were the most-differentially H3Ac-assocated candidate DEGs in Tc2 cells, respectively (**Figure 4D**). Thus, TCR re-stimulation was associated with a substantially greater number of transcriptional changes accompanied by promoter-proximal H3Ac remodeling, particularly during Tc1 differentiation.

**Figure 4:**
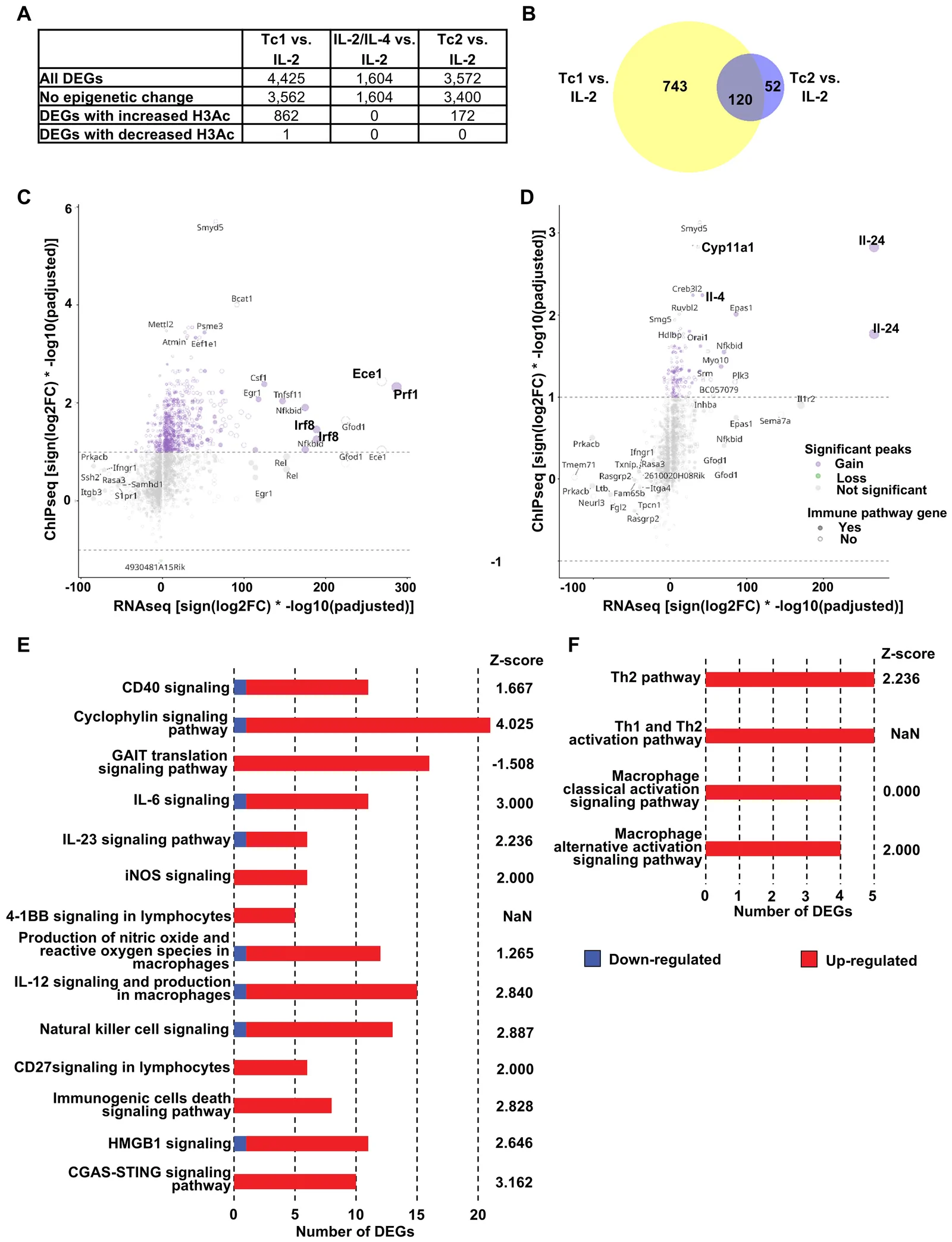
Integration of global transcriptional and epigenetic data identifies condition-specific H3Ac-associated differentially-expressed genes (DEGs) following TCR stimulation in CD8^+^ T cells. Integrated RNA-seq (fold change > |1.5|, P_adjusted_ < 0.05) and H3Ac ChIP-seq (P_adjusted_ < 0.10) data were analyzed in CD8^+^ T cells differentiated in IL-2 or IL-2/IL-4 in the presence or absence of TCR re-stimulation, with all comparisons normalized to the IL-2 baseline condition. Data represent 3 independent experiments. **(A)** Number of total DEGs identified by RNA-seq data associated with no epigenetic change, increased H3Ac, or decreased H3Ac relative to IL-2 alone. **(B)** Venn diagram showing overlapping and condition-specific cellular immune response DEGs linked with coordinated epigenetic and transcriptional changes in Tc1 and Tc2 cells after normalization to the baseline (IL-2 alone). Scatter plots showing H3Ac-associated DEGs **(C)** in Tc1 cells or **(D)** Tc2 cells compared to cells cultured in IL-2 alone. The axes depict values for sign(log2 fold change)*–log10 P_adjusted_-values comparing RNA-seq data (x-axis) and ChIP-seq data (y-axis) for each gene to facilitate visualization of both the respective direction of change and the magnitude of significance. Differentially-expressed cellular immune response pathways were identified by IPA^®^ (BH P < 0.05) using significant H3Ac-associated DEGs derived from the Venn diagram shown in panel B for unique to **(E)** Tc1 cells or **(F)** Tc2 cells. Blue bars represent the number of down-regulated DEGs and red the number of up-regulated DEGs within each pathway.

Despite a substantial overlap, the majority of H3Ac-associated DEGs were condition-specific, indicating distinct epigenetically coupled transcriptional programs during Tc1 and Tc2 differentiation. Comparison of H3Ac-associated DEGs between Tc1 and Tc2 cells relative to their baseline identified 743 genes unique to Tc1 cells, 52 genes unique to Tc2 cells, and 120 genes shared between both differentiation states (**Figure 4B**). A heatmap of the H3Ac-associated DEGs unique to Tc1 or Tc2 further highlighted distinct epigenetically coupled with transcriptional programs associated with differential patterns across the two CD8^+^ T cell differentiation states (**Figure 5**).

**Figure 5:**
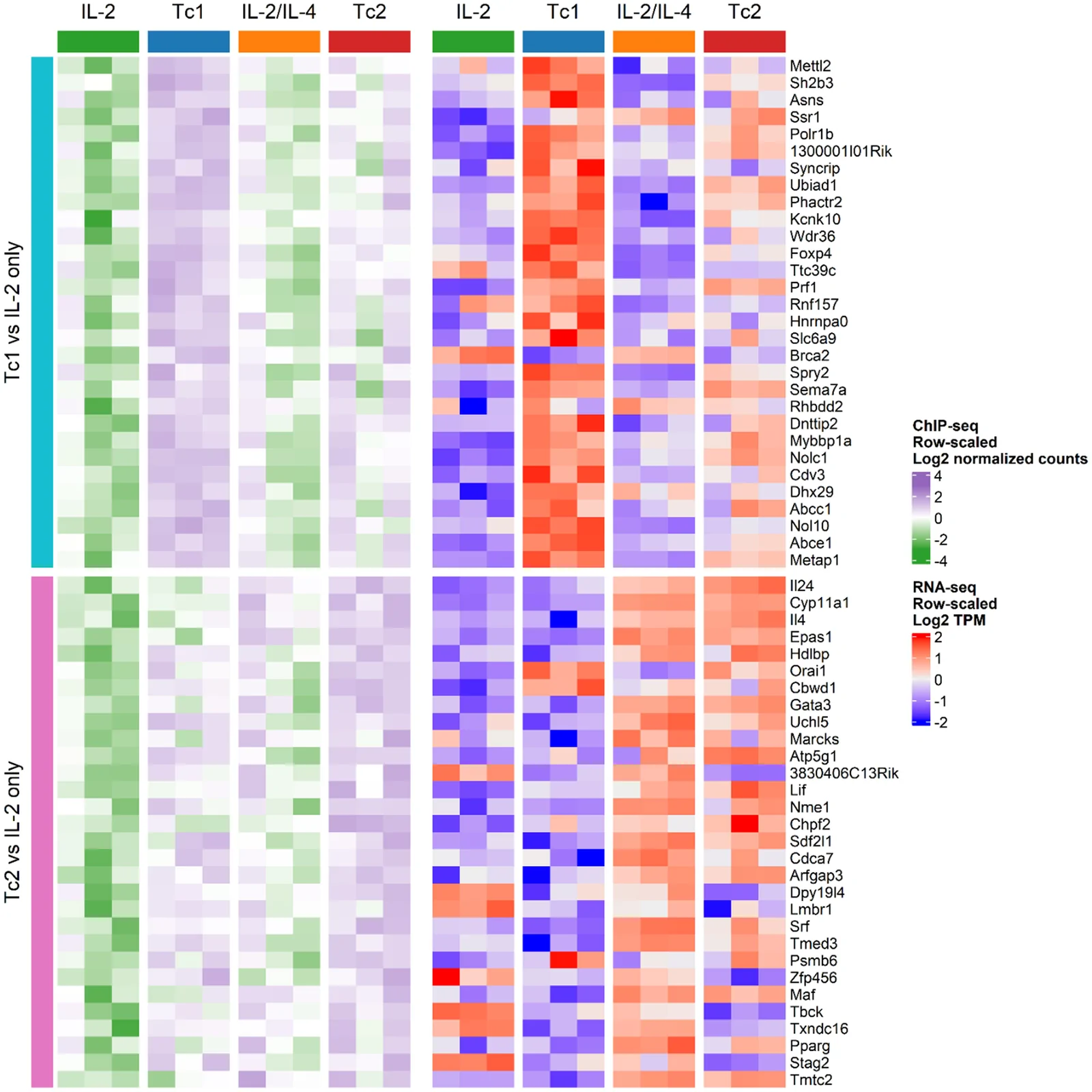
Heatmap of integrated ChIP-seq and RNA-seq analyses. Heatmap depict the ChIP-seq and RNA-seq normalized counts for select H3Ac-associated DEGs. Shown are the data for genes associated with the top 30 peaks by log2 fold change that were unique to the Tc1 vs IL-2 differential H3Ac peaks or unique to the Tc2 vs IL-2 differential H3Ac peaks from **Figure 4B**. Data shown are row-scaled log2 normalized counts for each assay across all replicates in all differentiation condition.

Next, pathway analyses of unique H3Ac-associated DEGs (**Figure 4B**) revealed functional programs linked to condition-specific epigenetic remodeling. In Tc1 cells, 14 cellular immune response pathways reached significance after BH correction, including CD40, IL-6, IL-23, iNOS, IL-12, 4-1BB, CD27, natural killer cell, HMGB1, and cGAS-STING signaling pathways (**Figure 4E**). The DEGs contributing to these pathways were predominantly upregulated, with only one gene (*Pik3cd*) showing downregulation, consistent with broad activation of inflammatory and effector-associated programs in Tc1 cells. In contrast, four cellular immune response pathways reached significance in Tc2 cells, including the Th2 pathway, Th1 and Th2 activation, as well as classical and alternative macrophage activation signaling (**Figure 4F**). Notably, the Tc2-specific H3Ac-associated gene set included *Gata3, Il-24, Il-4*, and *Ccr8*, highlighting a transcriptional program associated with type 2 immune differentiation. Upstream regulator analyses further identified *Il-33* (z-score=2.729, p-value of overlap=3.88E-07), *Gata3* (z-score=1.968, p-value of overlap=1.07E-06), and the *Stat6* dimer complex (z-score=1.980, p-value of overlap=1.25E-06) among the top three regulators associated with the Tc2-specific transcriptional program. Graphical network summaries of IPA^®^ pathway interactions further illustrated the distinct organization of signaling modules across conditions, supporting differential prioritization of pathways as well as regulatory elements in Tc1 and Tc2 cells (see **Supplementary Figures 9A, B**). These data further supported the notion that TCR re-stimulation within distinct cytokine environments drives epigenetically-coupled transcriptional programs, with Tc1 cells exhibiting broader inflammatory pathway engagement and Tc2 cells showing more lineage-associated type 2 immune specialization.

### 3.5 Pharmacologic histone acetylation alters Tc differentiation and lung allergic responses in mice

The functional impact of histone acetylation during Tc2 differentiation was assessed using the histone acetyltransferase inhibitor C646 during *in vitro* differentiation. C646-treated CD8^+^ T cells cultured in IL-2/IL-4 followed by TCR re-stimulation exhibited elevated IL-13 expression and reduced IFNγ production compared with untreated controls (**Figures 6A–D**). To determine if altered histone acetylation during Tc2 differentiation affects lung allergic immune responses, C646-treated CD8^+^ T cells cultured in IL-2/IL-4 were adoptively transferred into allergen-sensitized CD8-deficient mice prior to secondary allergen challenge (**Figure 6E**) (6, 7, 18). Mice receiving C646-treated cells developed significantly enhanced airway hyperresponsiveness (AHR) compared with CD8-deficient mice receiving CD8^+^ T cells differentiated in the absence of C646 (**Figure 6F**). Total cell numbers and eosinophils in the lung were increased, respectively (**Figure 6G**). The combination of *in vitro* and *in vivo* findings indicates that pharmacologic perturbation of histone acetylation during CD8^+^ T cell differentiation modifies cytokine production and enhances the capacity of Tc2 cells to promote allergic airway inflammation.

**Figure 6:**
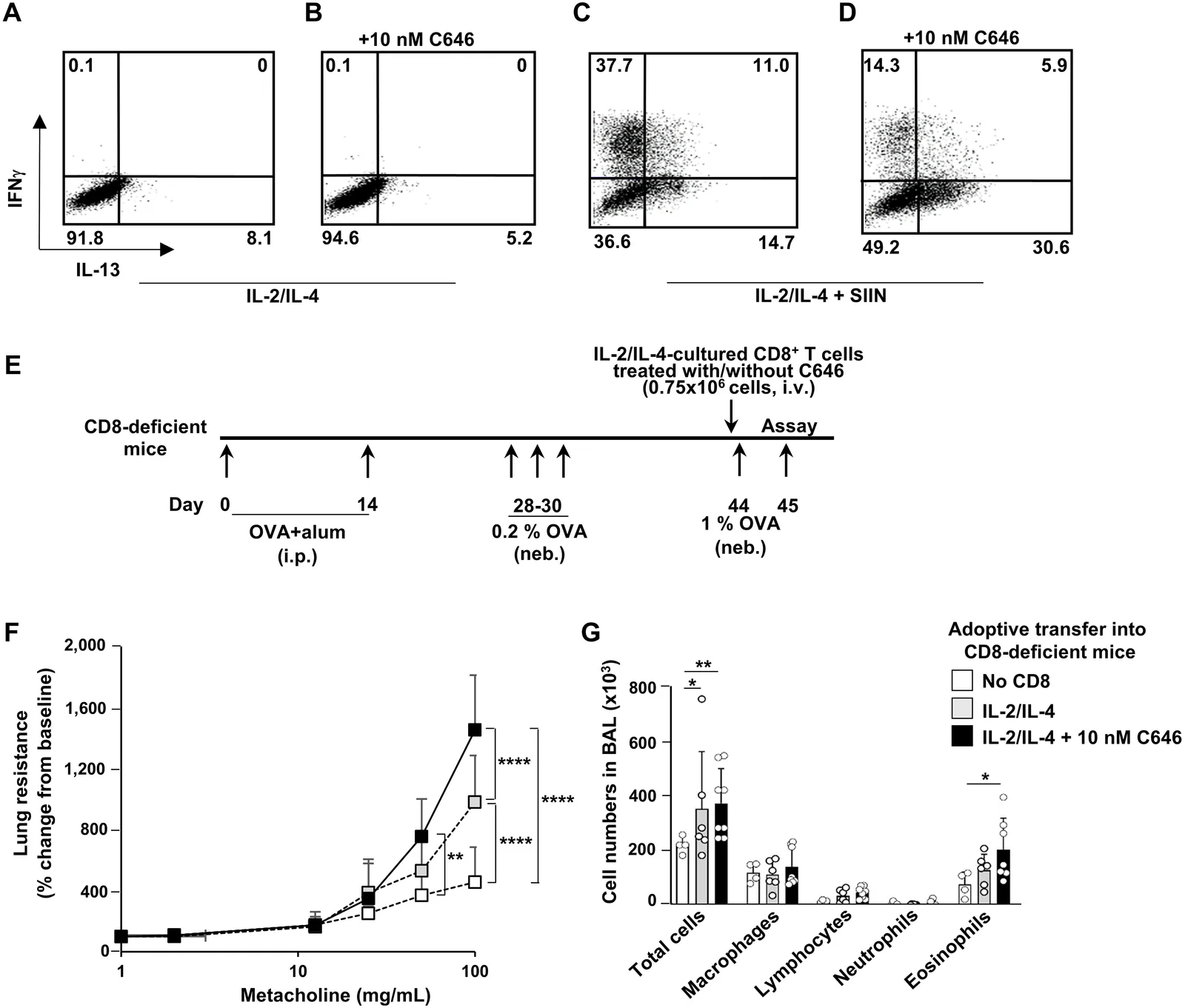
IFNγ and IL-13 cytokine expression is shifted in response to treatment with the histone acetyltransferase inhibitor C646 during *in vitro* differentiation of mouse CD8^+^ T cells which enhances airway hyperresponsiveness (AHR). **(A-D)** Flow cytometry analysis of IFNγ and IL-13 cytokine expression in CD8^+^ T cells differentiated *in vitro* in an IL-4-rich environment with or without TCR re-stimulation in the presence or absence of the epigenetic drug C646 (10 nM, p300 histone acetyltransferase inhibitor). **(E)** Secondary allergen model of experimental asthma using male and female CD8-deficient mice; CD8^+^ T cells were differentiated with IL-2/IL-4 with or without pre-treatment of 10 nM C646 before adoptive transfer into sensitized and challenged mice CD8-deficient mice followed by a secondary allergen challenge (day 44). **(F)** Changes of AHR in CD8 T cell-deficient recipients after adoptive transfer of IL-2/IL-4-differentiated CD8^+^ T cells in the presence or absence of 10 nM C646 from 2 independent experiments. No CD8^+^ T cells: n = 5; Tc2 cells: n = 7; Tc2 cells + 10 nM C646: n = 7. In each of the 2 independent experiments (total n = 9), 1 recipient mouse receiving C646-pretreated Tc2 cells had to be excluded for AHR analyses due to excessive mucus production. Data is presented as mean ± SD. **(G)** Composition in the bronchoalveolar lavage (BAL) fluid of CD8-deficient mice after adoptive transfer of IL-2/IL-4-differentiated CD8^+^ T cells in the presence or absence of 10 nM C646 from 2 independent experiments. No CD8^+^ T cells: n = 4; Tc2 cells: n = 6; Tc2 cells + 10 nM C646: n = 7. In each of the groups, 1 sample could not be analyzed due to excessive red blood cells in the BAL. Data is presented as mean ± SD. *P < 0.05, **P < 0.01, ****P < 0.0001.

### 3.6 Transcriptomic profiling of CD8^+^ T cells from steroid-resistant asthmatics

To explore whether human CD8^+^ T cells from SR asthmatics exhibit transcriptional differences compared to healthy controls, we performed whole-transcriptome profiling of *ex vivo* anti-CD3/CD28 activated peripheral blood CD8^+^ T cells from a small cohort of SR asthmatics and healthy controls. Despite the small sample size, we identified 155 genes (P_adjusted_ < 0.05) that were differentially expressed between groups, comprised of 120 up-regulated and 35 down-regulated transcripts (**Figure 7A**; see **Supplementary Figure 10**). The most statistically significant DEGs were *BCAS4*, *EIF5AL1*, and *HNRNPA1L2* (P_adjusted_ < 3.15E-36) but have not been directly linked to respiratory disease. Several immune-related genes were also differentially expressed, among them *IFITM3* (28, 29)*, MTRNR2L8* (30, 31), and *RSPO1* (32, 33). Importantly, *IL-13* expression was significantly increased in SR asthmatics compared to controls (P_adjusted_ = 0.0207), consistent with enhanced type 2–associated transcriptional skewing. Pathway analysis using IPA^®^ identified 12 significantly enriched pathways distinguishing SR asthmatics from controls, such as CXCR4 signaling, granzyme A Th1 and Th2 cell activation pathways, and the Th2 pathway (P < 0.05; **Figure 7B**; **Table 1**). The GAIT translation signaling and CXCR4 signaling pathway remained significant after BH correction.

**Figure 7:**
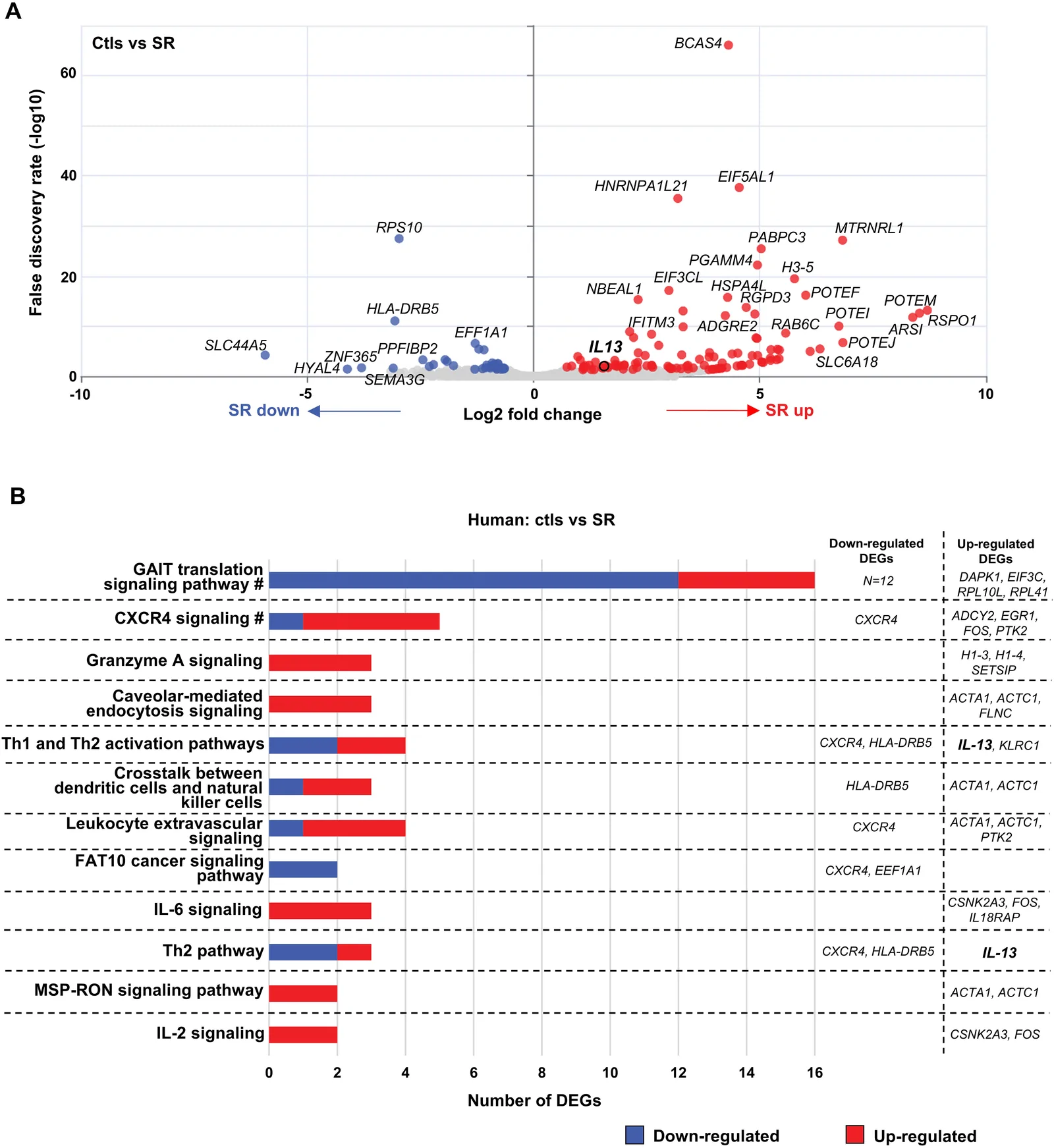
Global profiles of differentially-expressed genes (DEGs) and cellular immune response pathways in peripheral CD8^+^ T cells from human steroid-resistant (SR) asthmatics vs. healthy controls. **(A)** Volcano plot showing DEGs in peripheral CD8^+^ T cells from human healthy controls (n = 2) vs. SR asthmatics (n = 3) after 8 days in culture. The x-axis represents log2 fold change and the y-axis represents -log10 P_adjusted_-value. Red circles indicate DEGs with a fold change > |1.5| and a P_adjusted_ < 0.05. **(B)** Differentially-expressed cellular immune response pathways based on DEGs (fold change > |1.5| and a P_adjusted_ < 0.05) were identified by IPA^®^ in controls vs. SR asthmatics. Only pathways with a significance of P < 0.05 in IPA^®^ are shown and pathways sustaining Benjamini Hochberg (BH) correction are marked (#). Blue bars represent the number of down-regulated genes and red the number of up-regulated genes within the respective pathway.

**Table 1:** Overlapping cellular immune response pathways enriched for DEGs (fold change > |1.5|, P_adjusted_ < 0.05) were identified integrating IPA^®^ pathway enrichment results from human (healthy controls vs. steroid-resistant asthmatics) and mouse (Tc2 vs. IL-2) CD8^+^ T cells.

Overlapping cellular immune response pathways	Human DEGs (ctl vs SR)	P-value	Mouse DEGs (Tc2 vs IL-2)	BH P-value
CXCR4 signaling	*ADCY2, CXCR4*, ***EGR1***, ***FOS***, ***PTK2***	0.0022	*Adcy3, Adcy5, Addy7, Akt3, Cd4*, ***Egr1****, Elk1, Elmo2, Fgfr1*, ***Fos****, Gnai1, Gngi2, Gng2, Itpr1, Mapk3, Myl10, Pak6, Pik3c2a, Pik3r1, Pik3r5, Prkcb, Prkce, Prkcq, Prkcz, Prkd3*, ***Ptk2****, Rads2, Rhobtb2, Rhod, Rhof, Rhoh, Rhoq, Rnd1, Rnd2, Rnd3, Rock2, Rras, Rras2*	0.0017
Granzyme A signaling	*HIST1H1E, HIST1H1D, SETSIP*	0.0074	*Coxfa4, Fn1, Gzma, H1-2, Hmgb2, Lmn1, Mt-nd1, Mt-nd2, Mt-nd4, Mt-nd5, Mt-n6, Ndufab1, Ndufb6, Nme1, Prf1, Serpinb6b*	0.0437
Caveolar-mediated endocytosis signaling	*ACTA1, ACTC1, FLNC*	0.0087	*Abl1, Cavin1, Copa, Cope, Copg1, Egfr, Flnb, Hla-a, Insr, Itga1, Itga3, Itga4, Itga5, Itga6, Itgax, Itgb1, Itgb7, Itsn1, Rab5b*	0.0019
Th1 and Th2 activation pathway	***CXCR4***, ***HLA-DRB5***, ***IL-13****, KLRC1*	0.0151	*Acvr1, Acvr1b, Bhlhe41, Ccr1*, ***Ccr4***, *Ccr5*, *Ccr8, Cd274, Cd4, Cd40lg, Cd80, Cd8a, Cxcr3, Dll1, Gata3, Ghi1, H2-ab1, H2-dmb1, H2-eb1, H2-eb2, Havcr2, Hla-a, Hla-dma, Hla-dmb, Hla-doa, Hla-dqa1, Hla-dqb1*, ***HLA-drb5****, Icoslg, Ifnαr1, Ifnγ, Ifnγr1, Il-3, Il-4, Il-5, Il-6, Il-10*, ***Il-13****, Il-24, Il-1rl1, Il-2ra, Il-10ra, Il-10rb, Il-12rb1, Il-18r1, Jag1, Jag2, Jak1, Jak2, Lgals9, Maf, Nfatc1, Nfatc3, Nfkβ1, Notch1, Pik3c2a, Pik3r1, Pik3r5, Prkcq, S1pr1, Socs3, Stat5a, Tbx21, Tgfβ1, Tgfβr1, Tgfβr2, Tnfrsf4, Tnfsf11*	6.31E-15
Crosstalk between dendritic cells and natural killer cells	*ACTA1 ACTC1*, ***HLA-DRB5***	0.0158	*Camk2b, Camk2d, Ccr7, Cd226, Cd40lg, Cd80, Cd83, Csf2, Csf2rb, Csf2rb, Fas, H2-eb1, Hla-a*, ***Hla-drb5****, Ifnγ, Il-3, Il-4, Il-6, Il-15ra, Kldr1, Ltb, Nfkβ1, Nfkβ2, Prf1, Rel, Tlr7, Tlr9, Tnf, Tyrobp*	1.12E-04
Leukocyte extravasation signaling	*ACTA1, ACTC1, CXCR4*, ***PTK2***	0.0468	*Abl1, Actn4, Arhgap4, Arhgap9, Btk, Crkl, Ctnnb1, Ctnnd1, Cttn, Cybb, Edil3, Fgfr1, Gnai1, Itga3, Itga4, Itgb1, Map3k4, Mapk11, Mmp9, Mmp10, Mpm12, Mmp13, Mmp14, Ncf1, Ncf2, Pecam1, Pik3c2a, Pik3r1, Pik3r5, Plcg2, Prkcb, Prkce, Prkcq, Prkcz, Prkd3*, ***Ptk2****, Rapgef3, Rapgef4, Rassf5, Rhoh, Rock2, Selplg, Sipa1, Tec, Timp2, Txk, Vcl*	7.94E-05
FAT10 cancer signaling pathway	*EEF1A1, CXCR4*	0.0347	*Acvr1, Acvr1b, Akt3, Ctnnb1, Hoxb9, Ifnγ, Il-6, Nfkb1, Nfkb2, Nfkib, Nfkid, Rel, Tgfb1, Tgfbr1, Tgfbr2, Tnf, Tnfrsf11b*	0.0005
IL-6 signaling	*CSNK2A3*, ***FOS, IL-18RAP***	0.0032	*Abcb1, Akt3, Elk1, Elp1*, ***Fos***, ***Il-18rap****, Il-1a, Il-1r1, Il-1r2, Il-1rap, Il-1rl1, Il-6, Jak2, Lbp, Map2k3, Mapk11, Map3, Nfkb1, Nfkb2, Nfkbib, Nfkbid, Pik3c2a, Pik3r1, Pik3r5, Rasd2, Rel, Rras, Rras2, Socs3, Sos2, Srf, Tab1, Tnf, Tnfrsf11b, Vegfa*	4.47E-05
Th2 pathway	***CXCR4****, HLA-DRB5*, ***IL-13***	0.0407	*Acvr1, Acv1b, Bhlhe41, Cccr1*, ***Ccr4****, Ccr8, Cd4, Cd80, Dll1, Gata3, Gfl1, H2-ab1, H2-dmb1, H2-eb1, H2-eb2, Hla-a, Hla-dma, Hla-dmb, Hla-doa, Hla-dqa1, Icoslg, Infγ, Il-3, Il-4, Il-5, ILl10*, ***Il-13****, Il-24, Il-31, Il1rl1, Il-2ra, Il-12rb1, Jag1, Jag2, Jak1, Jak2, Maf, Nfkb1, Notch1, Pik3c2a, Pik3r1, Pik3r5, Prkcq, S1pr1, Socs3, Stat5a, Tbx21, Tgfb1, Tgfbr1, Tgfbr2, Tnfrsf4*	6.31E-11
MSP-RON signaling pathway	*ACTA1, ACTC1*	0.0437	*Ccr2, Csf1, Csf2rb, Csf2rb2, Ifnγ, Il-3, Jak2, Mst1r, Nos2, Pic3c2a, Pic3r1, Pik3r5, Prkcz, Rps6ka2, Tnf*	0.0219
IL-2 signaling	*CSNK2A3, FOS*	0.0457	*Akt3, Elk1*, ***Fos*,** *Il-2rs, Jak1, Mapk3, Pik3c2a, Pik3r1, Pik3r5, Rasd2, Rras, Rras2, Sos2, Stat5a*	0.0490

For each overlapping pathway, IPA^®^ enrichment significance [human: P < 0.05, mouse; Benjamini-Hochberg (BH) P < 0.05) and associated DEGs are shown separately for human and mouse analyses. DEGs present in both species within a given pathway are highlighted in bold to indicate cross-species overlap at the gene level.Bold values indicate overlapping DEGs between human and mouse datasets.

Cross-species integrative analysis was performed by comparing cellular immune response pathways enriched for DEGs (fold change > |1.5|, P_adjusted_ < 0.05) in human CD8^+^ T cells from SR asthmatics versus controls and in mouse Tc2 versus IL-2-treated CD8^+^ T cells. Pathway enrichment analyses by IPA^®^ identified 12 significantly enriched immune pathways in human CD8^+^ T cells from SR asthmatics (P < 0.05) and 60 in mouse Tc2 cells (BH P < 0.05). Among these, 11 pathways were mutually identified in both species (**Table 1**). Notably, several genes, including *IL-13*, *EGR1*, and *FOS* were among the shared DEGs in both species (**Table 1**). Together, these initial findings suggest mouse Tc2 cells recapitulate key immune pathways observed in human SR asthmatics, supporting the relevance of this model further defining conserved transcriptional programs associated with pro-allergic CD8^+^ T cell responses.

## 4 Discussion

Despite the introduction of several new biologics, severe asthma continues to represent a significant clinical challenge. IL-13–producing CD8^+^ T cells have been implicated in severe and SR asthma (6–8, 10, 15), while the molecular architecture underlying their lineage commitment has remained poorly defined. This study demonstrates that differentiation of CD8^+^ T cells to Tc1 or Tc2 cells reflects transcriptional and epigenetic remodeling, with distinct programs emerging according to their cytokine and TCR stimulation environment. Cross-species integration of genome-wide transcriptional analyses of mouse and human CD8^+^ T cells identified shared immune pathways associated with a Tc2 phenotype. These findings refine current concepts of CD8^+^ T cell plasticity and provide a mechanistic framework for how pathogenic type 2 responses may become established in severe asthma.

The therapeutic landscape of severe asthma has been transformed by biologic agents targeting type 2 inflammatory pathways, including IL-5 or its receptor, IL-4Rα, and thymic stromal lymphopoietin (TSLP) (34–37). Nevertheless, type 2 inflammation can persist despite modulation of individual cytokine pathways, underscoring the complexity of disease regulation. Our transcriptional analyses revealed distinct functional programs associated with Tc1 and Tc2 differentiation. Pathway-level analyses of DEGs indicating that Tc1-associated programs are enriched for inflammatory and immune activation pathways including antiviral signaling networks. In contrast, Tc2 cells exhibited a type 2-associated transcriptional program, including prominent expression of *Il-4*, *Il-13*, *Cyp11a1*, as well as *Il-24*. Notably, IL-24 was among the most strongly induced genes following IL-4 exposure. IL-24 is a member of the IL-10 family and is considered a type 2 cytokine contributing to inflammatory diseases such as allergic rhinitis (38) and atopic dermatitis (39). Our findings suggest that although Tc1 and Tc2 cells share components of an activation-associated transcriptional response, unique functional programs according to their differentiation environment are acquired. IL-4 appears to shape this trajectory early during differentiation, prior to full effector cell activation, extending previous candidate gene studies examining expression levels of Tc1 and Tc2 transcription factors (18). If Tc2 differentiation is reinforced by transcriptional activation, effector function may be maintained even when upstream cytokine signals are therapeutically attenuated. This perspective extends current models of type 2 inflammation by highlighting durable cellular programming as an additional layer that may contribute to persistent inflammation in severe asthma.

Epigenetic regulation has been increasingly recognized as a determinant of CD8^+^ T cell differentiation and memory formation (40, 41). Histone acetylation and chromatin accessibility at promoter and enhancer regions facilitate lineage-specific gene expression programs (40, 42, 43). Our data extend this framework to Tc1 and Tc2 differentiation, demonstrating dynamic and condition-specific H3Ac remodeling during CD8^+^ T cell differentiation. TCR re-stimulation resulted in a broad increase in H3Ac peaks in both CD8^+^ T cell subsets mainly in promoter regions. These observations are consistent with models in which promoter priming precedes robust transcriptional activation (40) and suggest that chromatin remodeling contributes to stabilizing lineage identity in an atopic environment. However, integration of H3Ac and transcriptional data revealed distinct patterns of epigenetically associated gene regulation. Tc1 cells exhibited a broader H3Ac-associated transcriptional program involving inflammatory and immune activation, whereas Tc2 cells showed a more focused set of H3Ac-associated DEGs linked to type 2 immune functions, enriched for genes within Th2 and Th1/Th2 activation pathways. *Il-24, Cyp11a1*, and *Il-4* were among the prominent Tc2-specific H3Ac-associated genes, providing an epigenetic correlate of the type 2 transcriptional program identified above. In contrast, Tc1-specific H3Ac-associated genes included the cytotoxic effector gene *Prf1* (44, 45), the metalloprotease *Ece1* (46), and the type 1-associated transcription factor *Irf8* (47), reflecting a more diverse immune activation program. Building on previous evidence that histone modifications at the *Il-13* and *Gata3* loci contribute to CD8^+^ T cell effector and memory states (18), suggests that histone acetylation contributes to the differential epigenetic programming of Tc1 and Tc2 cells, with Tc2 differentiation characterized by selective remodeling of loci associated with type 2 effector function. Together, these observations position histone acetylation as a structural regulator of CD8^+^ type 2 lineage stability within an atopic inflammatory context.

An emerging body of work proposes that Tc2 differentiation may not be exclusively dependent on IL-4 but could also be shaped by epithelial-derived alarmins such as IL-33, particularly in the lung (10). IL-33 activates a range of immune cells, including ILC2s (3) and mast cells (48), which can modulate the cytokine environment to favor type 2 immunity. While IL-33 receptor (ST2/IL1RL1) expression has been detected on subsets of CD8^+^ T cells (49), it remains unclear whether IL-33 acts directly on these cells or through intermediary cell types to enhance Tc2 polarization. Our experimental model was intentionally designed to examine intrinsic molecular mechanisms governing CD8^+^ T cell plasticity under defined cytokine conditions and therefore did not address the potential cellular crosstalk mediated by IL-33 *in vivo*. Nonetheless, transcriptomic analyses revealed broad remodeling of IL-33-associated signaling networks during Tc2 and Tc1 differentiation. In parallel, IL-33 was identified as an upstream regulator of the Tc2-specific H3Ac-associated DEG program, suggesting a potential link between IL-33-related signaling and the epigenetically coupled transcriptional program characteristic of Tc2 cells. Future studies leveraging co-culture models or conditional deletion of IL-33 or ST2 in specific cell types could clarify whether IL-33 contributes to Tc2 differentiation through direct signaling or via modulation of the broader immune landscape. Understanding these interactions may provide important insight into the complex network sustaining type 2 inflammation in asthma.

The functional consequences of epigenetic modulation further support a causal role for chromatin remodeling in directing CD8^+^ T cell fate. Pharmacological inhibition of histone acetyltransferase activity during differentiation altered cytokine output, and enhanced allergic immune responses following adoptive transfer. This demonstrated that perturbation of epigenetic programming can reshape effector function *in vivo*. These findings contrast with studies reporting reduced airway function, in part due to altered recruitment of p300 to the *Ormdl3* promoter (50). Whereas systemic model administration, as used in this model, may influences multiple immune and structural cells, our approach selectively modified CD8^+^ T cells *ex vivo* prior to their transfer into recipient mice. This highlights the complexity of targeting epigenetic regulators therapeutically and suggests that the effects of such interventions may depend on the timing, the specific cell type, and biological context.

The relevance of these mechanisms to human disease is supported by the overlap between mouse Tc2 transcriptional signatures and gene expression patterns observed in CD8^+^ T cells from SR asthmatics. Previous work demonstrated increased frequencies of activated and IL-13–producing CD8^+^ T cells in severe asthma (16), as well as relative corticosteroid insensitivity (10). The enrichment of Th1/Th2 pathways, together with human-mouse overlap of immune pathways and genes such as *IL-13*, *EGR-1*, and *FOS*, which are known to contribute to asthma pathogenesis (7, 15, 51–56). Our cross-species integration indicates that conserved regulatory networks may underlie pathogenic CD8^+^ T cell responses. Although our study does not directly assess chromatin remodeling in samples from asthmatics, the shared transcriptional architecture suggests that epigenetic mechanisms may contribute to the persistence or stabilization of Tc2 phenotypes in severe disease.

While the study provides mechanistic insight into Tc2 differentiation, several limitations warrant consideration. The human cohort was small, limiting the statistical power and generalizability of the transcriptomic comparisons. Therefore, the cross-species findings should be considered supportive and hypothesis-generating. The *in vitro* differentiation system, while highly controlled, cannot fully recapitulate the complex and dynamic cellular and tissue microenvironment in the asthmatic lung. While pharmacologic inhibition of histone acetyltransferases provided functional evidence linking acetylation to Tc2 function *in vivo*, the inhibitor C646 is not exclusively specific to H3 acetylation, and systemic effects as well as off-target consequences cannot be excluded. Despite these limitations, the combination of mouse mechanistic studies on a genome level and cross-species transcriptomic integration provides a framework for understanding how CD8^+^ T cell plasticity may contribute to persistent type 2 inflammation in severe asthma.

## Data Availability

The datasets presented in this study can be found in online repositories. The names of the repository/repositories and accession number(s) can be found below: https://www.ncbi.nlm.nih.gov/geo/, GSE109840 https://www.ncbi.nlm.nih.gov/geo/, GSE100624 https://www.ncbi.nlm.nih.gov/geo/, GSE109156.
