# Supplementary material for: Coordinated transcriptional and epigenetic regulation of type 2 CD8+ T cells in asthma
Source: Front Immunol. 2026 Oct 8;17:1837973. doi: 10.3389/fimmu.2026.1837973 (PMC13687325; doi:10.3389/fimmu.2026.1837973)
Supplement: Supplementary file 1 [file DataSheet1.docx]

Supplementary Material

# Supplementary Figures and Tables

## Supplementary Figures

**Supplementary Figure 1: Step-wise *in vitro* conversion of mouse CD8^+^ T cells to Tc1 (IFN**ɣ**^+^) or Tc2 (IL-13^+^) cells.** Staged differentiation protocol for *in vitro* CD8^+^ T cell conversion from IFNɣ- to IL-13-producing CD8^+^ T cells cultured in the presence of IL-2 or IL-2/IL-4 with or without re-stimulation of the T cell receptor (TCR) using SIINFEKL. Functional outcomes are measured on day 4 with or without TCR re-stimulation.

**Supplementary Figure 2: Hierarchical clustering analyses of RNA-seq data (fold change > |1.5|, P_adjusted_ < 0.05) of mouse CD8^+^ T cells differentiated in IL-2, IL2/TCR (Tc1), IL-2/IL-4 or IL2/IL-4/TCR (Tc2).** Results are depicted from 3 independent experiments using 1 mouse spleen per experiment to differentiate CD8^+^ T cells into Tc1 or Tc2 cells. TCR = T cell receptor.

**Supplementary Figure 3:** **Heatmap of the most differentially expressed genes (DEGs) in mouse CD8^+^ T cells differentiated in IL-2, IL2/TCR (Tc1), IL-2/IL-4 or IL2/IL-4/TCR (Tc2).** DEGs identified from the direct comparison of Tc2 cells to Tc1 cells [log2 FC ± 0.58 (equivalent to a fold change |1.5|), P_adjusted_ < 0.05] were ranked according to the highest positive or negative log2 fold change values. Heatmaps show the row scaled log2 transcripts per million (TPM) values of the top 30 up- and down-regulated DEGs across all experimental conditions. Results are derived from 3 independent experiments using 1 mouse spleen per experiment for differentiation of CD8^+^ T cells into Tc1 or Tc2 cells. TCR = T cell receptor.

**Supplementary Figure 4:** **Relative gene expression differences of master regulators within canonical Tc1 (*Irf8*, *Tbx21*, *IFNγ*) and Tc2 (*Il-4*, *Irf4*, *Cyp11a1*, *Gata3*, *Il-13)* genes during CD8^+^ T-cell differentiation in response to TCR re-stimulation (SIINFEKL) with or without exposure to IL-4.** **(A)** Increased log2-fold changes are shown in shades of red and decreased values in shades of blue. Log2 fold changes ±0.58 (corresponding to a fold change > |1.5|) and a P_adjusted_ < 0.05 were considered significant. Non-significant comparisons are marked (#). **(B)** Gene expression of master regulators reported as transcripts per million (TPM). Results are depicted from 3 independent experiments.

**Supplementary Figure 5. Unique immune cellular immune response pathways in (A) Tc1 and (B) Tc2 cells compared to cells cultured in IL-2 alone.** Differentially-expressed cellular immune response pathways were identified by Ingenuity Pathway Analysis® (IPA®, fold change > |1.5|, *P*_adjusted_ < 0.05).

**Supplementary Figure 6: Pan-H3 histone acetylation (H3Ac) during CD8^+^ T cell conversion.** H3Ac peaks were plotted relative to distance from the transcription start site (TSS) genome wide for each treatment. CD8^+^ T cells were differentiated in IL-2 = black, IL-2/TCR (Tc1) = red, IL-2/IL-4 = green or IL-2/IL-4/TCR (Tc2) = blue. Results are depicted from 3 independent experiments.

**Supplementary Figure 7: Pan-H3 histone acetylation (H3Ac) associated differentially expressed genes (DEGs) during CD8^+^ T cell differentiation.** H3Ac peaks were visualized using Integrated Genomics Viewer (IGV) within the promoter regions of **(A)** *Smyd5*, **(B)** *4930481A15Rik,* and **(C)** *Cyp11a1.* CD8^+^ T cells were differentiated in IL-2 (green), IL-2/TCR (Tc1, blue)**,** IL-2/IL-4 (orange), or IL-2/IL-4/TCR (Tc2, red)**.** Panel 1: Location of the differential consensus peaks within ± 1,000 bp of the transcription start site of the DEG. Panel 2: Shading of boxes represent number of replicates with a given called peak; the darkest shading represents peaks detected in all 3 independent experiments, whereas the lightest shading indicates peaks detected in a single experiment. Panel 3: H3Ac peak wiggle tracks of fold enrichment over background for each biological replicate and differentiation condition. Boxes represent the presence of individual called peaks for each experiment and differentiation condition. Panel 4: Sum of H3Ac peak wiggle tracks of fold enrichment over background. Results are depicted from 3 independent experiments.

**Supplementary Figure 8: Scatter plot showing H3Ac-associated DEGs (C) in IL-2/IL-4 differentiated cells compared to cells cultured in IL-2 alone.** The axes depict values for sign(log2 FC)*–log10 P_adjusted_-values comparing RNA-seq data (x-axis) and ChIP-seq data (y-axis) for each gene to facilitate visualization of both the respective direction of change and the magnitude of significance. Select gene names are annotated.

**Supplementary Figure 9: Graphical summaries of canonical pathways for Pan-H3 histone acetylation (H3Ac) associated differentially expressed genes (DEGs) unique to Tc1 and Tc2 cells.** Ingenuity Pathway Analysis® (IPA®) were performed using H3Ac-associated DEGs identified unique to **(A)** Tc1 or **(B)** Tc2 cells compared to cells cultured in IL-2 alone.

**Supplementary Figure 10:** **Heatmap of differentially expressed genes (DEGs) in peripheral CD8^+^ T cells from human healthy controls vs. steroid-resistant (SR) asthmatics.** DEGs (fold change |1.5|), P_adjusted_ < 0.05) identified between human healthy controls (n = 2) and SR asthmatics (n = 3) after 8 days in culture were ranked according to the log2 fold change. Heatmaps display log2 transcripts per million (TPM) values for all 155 DEGs across individual samples.

## 1.1 Supplementary Tables

**Supplementary Table 1:** Ingenuity Pathway Analysis® (IPA®) for down- and up-regulated differentially-expressed genes (DEGs) of Tc1 cells compared to cells cultured in IL-2 alone (fold change > |1.5|, P_adjusted_ < 0.05). Ranking of canonical and cellular immune response pathways are displayed based on their overall ranking with a Benjamini-Hochberg (BH) P < 0.05. Unique canonical and cellular immune response pathways of Tc1 compared Tc2 cells are marked (X).

| **Ranking # canonical pathways** | **Ranking # immune pathways** | **Ingenuity canonical pathways** | **BH p-value** | **Z-score** | **Unique pathways**  **in Tc1** |
| --- | --- | --- | --- | --- | --- |
| 1 |  | Mitotic prometaphase | 7.94E-22 | -6.395 |  |
| 2 |  | RHO GTPase cycle | 1.00E-21 | -0.239 |  |
| 3 |  | rRNA modification in the nucleus and cytosol | 5.01E-20 | 6.856 |  |
| 4 |  | Cell cycle checkpoints | 6.31E-19 | -4.841 |  |
| 5 |  | Chronic myeloid leukemia signaling | 2.51E-15 | -1.320 |  |
| 6 |  | Senescence pathway | 2.51E-15 | -1.106 |  |
| 7 |  | Mitotic metaphase and anaphase | 5.01E-15 | -3.162 |  |
| 8 |  | Pancreatic adenocarcinoma signaling | 1.00E-14 | 1.333 |  |
| 9 |  | Cardiac hypertrophy signaling (enhanced) | 1.00E-14 | -0.447 |  |
| 10 |  | Small cell lung cancer signaling | 3.16E-14 | 1.400 |  |
| 11 |  | Mitotic G2-G2/M phases | 5.01E-14 | -2.907 |  |
| 12 |  | Protein kinase A signaling | 6.31E-14 | -2.475 |  |
| 13 |  | Cachexia signaling pathway | 1.26E-13 | 1.976 |  |
| 14 |  | IL-27 signaling pathway | 1.00E-12 | -1.753 |  |
| 15 |  | RHO GTPases activate formins | 3.16E-12 | -4.817 |  |
| 16 |  | Molecular mechanisms of cancer | 3.16E-12 | -1.583 |  |
| 17 |  | Major pathway of rRNA processing in the nucleolus and cytosol | 3.98E-12 | 9.168 |  |
| 18 |  | Hereditary breast cancer signaling | 5.01E-12 | -0.802 |  |
| 19 | 1 | Cyclophilin signaling pathway | 7.94E-12 | 1.309 |  |
| 20 |  | Tuberculosis latent signaling pathway | 7.94E-12 | 1.067 |  |
| 21 |  | Endocannabinoid cancer inhibition pathway | 7.94E-12 | 0.674 |  |
| 22 |  | Hepatic fibrosis signaling pathway | 1.26E-11 | 1.144 |  |
| 23 |  | Role of tissue factor in cancer | 2.00E-11 | 1.053 |  |
| 24 |  | NAFLD signaling pathway | 3.16E-11 | 3.989 |  |
| 25 |  | Mitotic G1 phase and G1/S transition | 3.16E-11 | 0.277 |  |
| 26 |  | Glycation signaling pathway | 3.98E-11 | 2.265 |  |
| 27 | 2 | HMGB1 signaling | 3.98E-11 | 0.447 |  |
| 28 |  | CLEAR signaling pathway | 5.01E-11 | -2.830 |  |
| 29 |  | Prostate cancer signaling | 5.01E-11 | -0.866 |  |
| 30 | 3 | Role of pattern recognition receptors in recognition of bacteria and viruses | 6.31E-11 | -1.414 |  |
| 31 |  | Autophagy | 1.00E-10 | -2.294 |  |
| 32 |  | RAR activation | 1.15E-10 | -4.017 |  |
| 33 |  | Cell cycle control of chromosomal replication | 1.17E-10 | -4.017 | X |
| 34 | 4 | CD40 signaling | 1.17E-10 | -0.180 |  |
| 35 |  | Role of CHK proteins in cell cycle checkpoint control | 1.38E-10 | 0.426 |  |
| 36 |  | PI3K/AKT signaling | 2.19E-10 | 1.769 |  |
| 37 |  | Coronavirus pathogenesis pathway | 2.29E-10 | 2.869 |  |
| 38 |  | Role of macrophages, fibroblasts and endothelial cells in rheumatoid arthritis | 2.57E-10 | 2.424 |  |
| 39 |  | Erythropoietin signaling pathway | 4.07E-10 | -3.048 |  |
| 40 |  | Kinetochore metaphase signaling pathway | 4.17E-10 | -3.000 |  |
| 41 |  | Myelination signaling pathway | 4.79E-10 | -1.320 |  |
| 42 |  | Colorectal cancer metastasis signaling | 4.79E-10 | -0.120 |  |
| 43 |  | Role of BRCA1 in DNA damage response | 4.90E-10 | -1.890 |  |
| 44 |  | ERK/MAPK signaling | 5.50E-10 | -1.638 |  |
| 45 |  | RAC signaling | 5.89E-10 | -0.156 |  |
| 46 |  | Ceramide signaling | 6.03E-10 | 1.333 |  |
| 47 |  | Superpathway of inositol phosphate compounds | 1.58E-09 | -0.535 |  |
| 48 |  | Hepatic cholestasis | 1.66E-09 | 1.780 |  |
| 49 |  | Inhibition of ARE-mediated mRNA degradation pathway | 1.95E-09 | 0.295 |  |
| 50 | 5 | Th1 and Th2 activation pathway | 2.34E-09 | NaN |  |
| 51 | 6 | PD-1, PD-L1 cancer immunotherapy pathway | 2.40E-09 | 1.121 |  |
| 52 |  | RNA m6A methylation signaling pathway | 2.40E-09 | 1.100 |  |
| 53 |  | Class I MHC mediated antigen processing and presentation | 2.95E-09 | 0.577 |  |
| 54 |  | Histone modification signaling pathway | 3.63E-09 | -2.278 |  |
| 55 |  | Renin-angiotensin signaling | 3.72E-09 | -0.822 |  |
| 56 |  | Warburg effect signaling pathway | 3.89E-09 | 2.598 |  |
| 57 |  | Signaling by Rho family GTPases | 3.89E-09 | 0.378 |  |
| 58 | 7 | IL-8 signaling | 3.89E-09 | 0.000 |  |
| 59 | 8 | IL-12 signaling and production in macrophages | 4.17E-09 | 2.194 |  |
| 60 |  | HER-2 signaling in breast cancer | 4.57E-09 | 0.620 |  |
| 61 |  | CGRP in inflammation and nociception signaling pathway | 5.13E-09 | -3.159 |  |
| 62 |  | Tumor microenvironment pathway | 5.37E-09 | 3.098 |  |
| 63 |  | Sertoli cell-germ cell junction signaling pathway (enhanced) | 5.75E-09 | -0.346 |  |
| 64 |  | Stress granule signaling pathway | 6.17E-09 | -1.688 |  |
| 65 |  | Eicosanoid signaling | 7.24E-09 | -2.588 |  |
| 66 |  | p53 signaling | 7.24E-09 | 0.522 |  |
| 67 |  | Sertoli cell-sertoli cell junction signaling | 8.51E-09 | -0.688 |  |
| 68 |  | Cilium assembly | 9.77E-09 | -4.765 |  |
| 69 |  | Folate signaling pathway | 9.77E-09 | 2.117 |  |
| 70 | 9 | Role of PKR in interferon induction and antiviral response | 1.10E-08 | -0.885 |  |
| 71 |  | XBP1(S) activates chaperone genes | 1.20E-08 | 2.353 |  |
| 72 |  | Hepatitis B chronic liver pathogenesis signaling pathway | 1.20E-08 | 0.263 |  |
| 73 |  | Non-small cell lung cancer signaling | 1.23E-08 | -1.706 |  |
| 74 |  | Synthesis of DNA | 1.32E-08 | -3.063 |  |
| 75 |  | PTEN signaling | 1.32E-08 | 0.316 |  |
| 76 |  | HGF signaling | 1.35E-08 | -0.822 |  |
| 77 |  | RANK signaling in osteoclasts | 1.51E-08 | -0.365 |  |
| 78 |  | Relaxin signaling | 1.62E-08 | -2.959 |  |
| 79 |  | Regulation of the epithelial mesenchymal transition by growth factors pathway | 1.62E-08 | 1.921 |  |
| 80 |  | Lymphotoxin β receptor signaling | 1.62E-08 | -0.209 |  |
| 81 |  | Cyclins and cell cycle regulation | 1.62E-08 | 0.000 |  |
| 82 |  | Glioma signaling | 1.70E-08 | -1.177 |  |
| 83 |  | Epithelial membrane protein signaling pathway | 1.70E-08 | -0.911 |  |
| 84 |  | 3-phosphoinositide degradation | 1.70E-08 | -0.152 |  |
| 85 |  | 3-phosphoinositide biosynthesis | 1.70E-08 | -0.149 |  |
| 86 |  | Semaphorin neuronal repulsive signaling pathway | 2.04E-08 | 2.941 |  |
| 87 |  | Insulin secretion signaling pathway | 2.34E-08 | -1.324 |  |
| 88 |  | Protein ubiquitination pathway | 2.88E-08 | 3.862 |  |
| 89 |  | HIF1α signaling | 2.95E-08 | -0.739 |  |
| 90 |  | ILK signaling | 2.95E-08 | -0.535 |  |
| 91 |  | NOD1/2 signaling pathway | 3.16E-08 | 2.324 |  |
| 92 |  | Activation of the pre-replicative complex | 3.39E-08 | -4.583 | X |
| 93 |  | Proteasomal PSMD10 signaling pathway | 3.63E-08 | -0.781 |  |
| 94 |  | Autism signaling pathway | 4.07E-08 | 1.528 |  |
| 95 | 10 | NF-κB activation by viruses | 4.27E-08 | -0.354 |  |
| 96 |  | Nuclear cytoskeleton signaling pathway | 5.37E-08 | -2.425 |  |
| 97 |  | Sumoylation pathway | 5.37E-08 | 1.061 |  |
| 98 |  | mTOR signaling | 5.37E-08 | -0.140 |  |
| 99 |  | RAF/MAP kinase cascade | 5.62E-08 | 2.717 |  |
| 100 |  | NGF-stimulated transcription | 6.46E-08 | 2.985 |  |
| 101 |  | Ovarian cancer signaling | 8.13E-08 | -2.449 |  |
| 102 |  | Cell cycle: G1/S checkpoint regulation | 8.13E-08 | 0.000 |  |
| 103 |  | NER (nucleotide excision repair, enhanced pathway) | 8.32E-08 | -1.732 |  |
| 104 |  | Pulmonary hypertension signaling pathway | 9.12E-08 | 0.606 |  |
| 105 |  | MSP-RON signaling in cancer cells pathway | 9.33E-08 | -0.729 |  |
| 106 |  | Virus entry via endocytic pathways | 1.05E-07 | -3.244 |  |
| 107 |  | Ovarian folliculogenesis signaling pathway | 1.10E-07 | -2.255 |  |
| 108 | 11 | IL-33 signaling pathway | 1.10E-07 | 1.483 |  |
| 109 |  | Preeclampsia signaling pathway | 1.23E-07 | 0.272 |  |
| 110 |  | ATM signaling | 1.26E-07 | -0.928 |  |
| 111 |  | LPS-stimulated MAPK signaling | 1.32E-07 | -0.539 |  |
| 112 | 12 | Production of nitric oxide and reactive oxygen species in macrophages | 1.45E-07 | 0.135 |  |
| 113 | 13 | Caveolar-mediated endocytosis signaling | 1.51E-07 | -2.236 |  |
| 114 |  | VEGF signaling | 1.58E-07 | -1.061 |  |
| 115 |  | Neutrophil degranulation | 1.70E-07 | -0.636 |  |
| 116 |  | Osteoarthritis pathway | 1.74E-07 | 2.774 |  |
| 117 |  | BER (base excision repair) pathway | 1.74E-07 | -2.132 | X |
| 118 |  | Pulmonary healing signaling pathway | 1.74E-07 | 1.921 |  |
| 119 |  | Gap junction signaling | 1.74E-07 | -1.207 |  |
| 120 | 14 | IL-9 signaling | 2.04E-07 | -0.258 |  |
| 121 |  | Asparagine N-linked glycosylation | 2.09E-07 | 1.769 |  |
| 122 |  | PPARα/RXRα activation | 2.29E-07 | -2.359 |  |
| 123 |  | P2Y purinergic receptor signaling pathway | 2.57E-07 | -2.722 |  |
| 124 |  | Sheddase signaling pathway | 2.57E-07 | 0.882 |  |
| 125 |  | Tuberculosis active signaling pathway | 2.63E-07 | -1.673 |  |
| 126 | 15 | Interleukin-4 and interleukin-13 signaling | 2.88E-07 | 2.655 |  |
| 127 |  | MicroRNA biogenesis signaling pathway | 2.88E-07 | 0.535 |  |
| 128 |  | Ribonucleotide reductase signaling pathway | 2.95E-07 | -1.483 |  |
| 129 |  | Pulmonary fibrosis idiopathic signaling pathway | 3.09E-07 | 2.804 |  |
| 130 |  | Acute myeloid leukemia signaling | 3.47E-07 | -1.177 |  |
| 131 |  | Germ cell-sertoli cell junction signaling | 3.89E-07 | NaN |  |
| 132 |  | Ferroptosis signaling pathway | 4.07E-07 | 0.447 |  |
| 133 | 16 | IL-17 signaling | 4.27E-07 | 2.474 |  |
| 134 | 17 | CD27 signaling in lymphocytes | 4.37E-07 | 2.236 |  |
| 135 |  | D-myo-inositol-5-phosphate metabolism | 4.37E-07 | -0.316 |  |
| 136 |  | Apelin pancreas signaling pathway | 4.57E-07 | -1.964 | X |
| 137 | 18 | IL-23 signaling pathway | 4.57E-07 | 1.091 |  |
| 138 |  | Transcriptional regulation by RUNX3 | 4.68E-07 | 2.795 |  |
| 139 |  | IGF-1 signaling | 4.68E-07 | -0.845 |  |
| 140 |  | ID1 signaling pathway | 4.68E-07 | -0.258 |  |
| 141 |  | Thrombin signaling | 4.90E-07 | -1.155 |  |
| 142 |  | GADD45 signaling | 5.25E-07 | -1.569 |  |
| 143 |  | Type II diabetes mellitus signaling | 6.61E-07 | 0.365 |  |
| 144 |  | JAK/STAT signaling | 7.24E-07 | -0.730 |  |
| 145 |  | Interleukin-2 family signaling | 7.24E-07 | 0.626 |  |
| 146 |  | D-myo-inositol (1,4,5,6)-tetrakisphosphate biosynthesis | 7.24E-07 | 0.507 |  |
| 147 |  | D-myo-inositol (3,4,5,6)-tetrakisphosphate biosynthesis | 7.24E-07 | 0.507 |  |
| 148 |  | Docosahexaenoic acid (DHA) signaling | 7.76E-07 | -2.077 |  |
| 149 |  | Mitochondrial division signaling pathway | 7.94E-07 | -1.236 |  |
| 150 |  | Wound healing signaling pathway | 1.02E-06 | 2.730 |  |
| 151 |  | Mitotic roles of polo-like kinase | 1.02E-06 | -1.291 |  |
| 152 |  | Telomerase signaling | 1.05E-06 | -1.300 |  |
| 153 |  | Growth hormone signaling | 1.10E-06 | -0.378 |  |
| 154 |  | Turbulent (oscillatory, disturbed) flow shear stress activates signaling by PIEZO1 and integrins in endothelial cells | 1.12E-06 | 2.357 |  |
| 155 |  | Pancreatic secretion signaling pathway | 1.12E-06 | -1.969 |  |
| 156 | 19 | IL-3 signaling | 1.26E-06 | -1.257 |  |
| 157 |  | Regulation of TP53 activity through phosphorylation | 1.38E-06 | -1.521 | X |
| 158 | 20 | GM-CSF signaling | 1.41E-06 | -0.784 |  |
| 159 |  | Lysosome positioning signaling pathway | 1.48E-06 | -0.566 |  |
| 160 |  | Neddylation | 1.51E-06 | 1.195 |  |
| 161 |  | Neuregulin signaling | 1.62E-06 | -0.192 |  |
| 162 |  | Estrogen-dependent breast cancer signaling | 1.70E-06 | -1.279 |  |
| 163 |  | TP53 regulates transcription of cell cycle genes | 1.70E-06 | -1.043 |  |
| 164 |  | CDK5 signaling | 1.74E-06 | -1.976 |  |
| 165 |  | CDX gastrointestinal cancer signaling pathway | 1.74E-06 | -1.069 |  |
| 166 |  | Role of chondrocytes in rheumatoid arthritis signaling pathway | 1.78E-06 | 2.654 |  |
| 167 |  | DNA methylation and transcriptional repression signaling | 1.78E-06 | 0.378 |  |
| 168 |  | Melanocyte development and pigmentation signaling | 2.34E-06 | -2.959 |  |
| 169 |  | B cell activating factor signaling | 2.34E-06 | 2.496 |  |
| 170 | 21 | Immunogenic cell death signaling pathway | 2.34E-06 | 1.616 |  |
| 171 |  | PDGF signaling | 2.34E-06 | -0.174 |  |
| 172 | 22 | Th2 pathway | 2.34E-06 | 0.156 |  |
| 173 |  | Glioblastoma multiforme signaling | 2.45E-06 | -0.309 |  |
| 174 |  | MYC mediated apoptosis signaling | 2.51E-06 | -1.706 |  |
| 175 |  | Gαq signaling | 2.63E-06 | -1.406 |  |
| 176 | 23 | CXCR4 signaling | 2.63E-06 | -1.043 |  |
| 177 | 24 | Macropinocytosis signaling | 2.75E-06 | -1.528 |  |
| 178 | 25 | IL-6 signaling | 2.82E-06 | 1.761 |  |
| 179 | 26 | Th1 pathway | 2.95E-06 | -0.316 |  |
| 180 |  | Role of IL-17A in arthritis | 3.47E-06 | NaN |  |
| 181 | 27 | FAT10 cancer signaling pathway | 3.72E-06 | 1.886 |  |
| 182 |  | eNOS signaling | 3.80E-06 | -3.244 |  |
| 183 |  | STAT3 pathway | 3.80E-06 | 0.522 |  |
| 184 |  | Regulation of cellular mechanics by calpain protease | 3.89E-06 | -2.065 |  |
| 185 | 28 | Natural killer cell signaling | 3.89E-06 | 0.405 |  |
| 186 |  | GNRH signaling | 3.98E-06 | -2.309 |  |
| 187 | 29 | Activation of IRF by cytosolic pattern recognition receptors | 4.27E-06 | 0.200 |  |
| 188 |  | Integrin to cytoskeleton signaling pathway | 4.37E-06 | -2.023 |  |
| 189 |  | Opioid signaling pathway | 4.37E-06 | -1.750 |  |
| 190 |  | tRNA charging | 4.79E-06 | 3.300 | X |
| 191 |  | p75 NTR receptor-mediated signaling | 4.79E-06 | -1.571 |  |
| 192 |  | Cell cycle regulation by BTG family proteins | 4.79E-06 | -1.265 |  |
| 193 |  | Epithelial adherens junction signaling | 4.79E-06 | 1.155 |  |
| 194 |  | G-Protein coupled receptor signaling | 4.79E-06 | -1.058 |  |
| 195 |  | Sleep REM signaling pathway | 4.79E-06 | 0.200 |  |
| 196 |  | Activin inhibin signaling pathway | 5.01E-06 | -0.640 |  |
| 197 |  | Cilia biogenesis signaling pathway | 5.25E-06 | -2.517 |  |
| 198 |  | Regulation of eIF4 and p70S6K signaling | 5.25E-06 | -1.961 |  |
| 199 |  | DYRK1A signaling pathway | 5.25E-06 | 0.555 |  |
| 200 |  | Endocannabinoid developing neuron pathway | 5.50E-06 | -1.414 |  |
| 201 |  | Signaling by VEGF | 5.75E-06 | 0.493 |  |
| 202 | 30 | fMLP signaling in neutrophils | 6.17E-06 | -2.795 |  |
| 203 |  | TNFs bind their physiological receptors | 6.17E-06 | 2.500 |  |
| 204 | 31 | IL-10 signaling | 6.17E-06 | -2.188 |  |
| 205 | 32 | MSP-RON signaling in macrophages pathway | 6.17E-06 | -1.808 |  |
| 206 |  | April mediated signaling | 6.17E-06 | 1.500 |  |
| 207 |  | Regulation of mitotic cell cycle | 6.17E-06 | -0.707 |  |
| 208 |  | Estrogen-mediated S-phase entry | 6.17E-06 | 0.258 |  |
| 209 |  | Axonal guidance signaling | 6.61E-06 | NaN |  |
| 210 |  | RHOGDI signaling | 6.92E-06 | -0.603 |  |
| 211 |  | UFMylation signaling pathway | 7.08E-06 | 0.784 |  |
| 212 |  | IL-17A signaling in fibroblasts | 8.32E-06 | 3.286 |  |
| 213 |  | PI metabolism | 8.32E-06 | -2.694 |  |
| 214 |  | PEDF signaling | 8.32E-06 | -1.633 |  |
| 215 |  | Reelin signaling in neurons | 8.51E-06 | 0.333 |  |
| 216 |  | Adrenomedullin signaling pathway | 9.12E-06 | -1.540 |  |
| 217 |  | cAMP-mediated signaling | 9.33E-06 | -3.101 |  |
| 218 |  | TNFR2 signaling | 9.55E-06 | 2.887 |  |
| 219 |  | Role of JAK1 and JAK3 in γc cytokine signaling | 1.02E-05 | -0.392 |  |
| 220 |  | Glycosaminoglycan metabolism | 1.07E-05 | -2.343 |  |
| 221 |  | Angiopoietin signaling | 1.07E-05 | -0.728 |  |
| 222 |  | Sirtuin signaling pathway | 1.15E-05 | -1.941 |  |
| 223 |  | Apelin endothelial signaling pathway | 1.15E-05 | -1.000 |  |
| 224 |  | Sphingosine-1-phosphate signaling | 1.23E-05 | -0.169 |  |
| 225 |  | UVA-induced MAPK signaling | 1.26E-05 | -2.236 |  |
| 226 |  | Huntington's disease signaling | 1.26E-05 | -2.121 |  |
| 227 |  | IL-1 signaling | 1.26E-05 | 0.894 |  |
| 228 |  | Role of osteoblasts, osteoclasts and chondrocytes in rheumatoid arthritis | 1.29E-05 | NaN |  |
| 229 |  | Insulin receptor signaling | 1.29E-05 | 0.480 |  |
| 230 |  | Actin cytoskeleton signaling | 1.41E-05 | -1.155 |  |
| 231 | 33 | CGAS-STING signaling pathway | 1.62E-05 | 3.086 |  |
| 232 |  | Irritable bowel syndrome signaling pathway | 1.62E-05 | 1.270 |  |
| 233 |  | TNFR1 signaling | 1.86E-05 | 1.414 |  |
| 234 |  | Glioma invasiveness signaling | 1.86E-05 | -1.177 |  |
| 235 |  | NGF signaling | 1.86E-05 | -1.029 |  |
| 236 | 34 | IL-7 signaling pathway | 1.86E-05 | -0.626 |  |
| 237 |  | Interleukin-1 family signaling | 1.91E-05 | 4.217 |  |
| 238 | 35 | Leukocyte extravasation signaling | 1.95E-05 | -0.566 |  |
| 239 |  | Resolution of abasic sites (AP sites) | 2.04E-05 | -2.357 | X |
| 240 |  | Interleukin-3, interleukin-5 and GM-CSF signaling | 2.04E-05 | 0.626 |  |
| 241 |  | UBE2C in cell cycle progression signaling pathway | 2.09E-05 | -2.121 |  |
| 242 |  | Prolactin signaling | 2.09E-05 | -0.928 |  |
| 243 |  | Pathogen induced cytokine storm signaling pathway | 2.14E-05 | 2.372 |  |
| 244 |  | KEAP1-NFE2L2 pathway | 2.14E-05 | 0.590 |  |
| 245 |  | DNA double-strand break repair by homologous recombination | 2.29E-05 | -2.646 |  |
| 246 |  | Thrombopoietin signaling | 2.29E-05 | -1.225 |  |
| 247 |  | AMPK signaling | 2.34E-05 | -3.355 |  |
| 248 |  | Multiple sclerosis signaling pathway | 2.34E-05 | 0.927 |  |
| 249 |  | PAK signaling | 2.34E-05 | -0.730 |  |
| 250 |  | TCR signaling | 2.45E-05 | 2.530 |  |
| 251 |  | Aryl hydrocarbon receptor signaling | 2.63E-05 | -0.480 |  |
| 252 |  | Death receptor signaling | 2.63E-05 | -0.378 |  |
| 253 |  | Paxillin signaling | 2.88E-05 | -1.177 |  |
| 254 |  | FLT3 signaling | 3.02E-05 | 0.471 |  |
| 255 |  | TNFR2 non-canonical NF-kB pathway | 3.09E-05 | 3.138 |  |
| 256 |  | Peroxisomal lipid metabolism | 3.16E-05 | -3.357 | X |
| 257 |  | Ephrin receptor signaling | 3.16E-05 | -1.622 |  |
| 258 |  | NAD signaling pathway | 3.31E-05 | -1.897 |  |
| 259 | 36 | Macrophage alternative activation signaling pathway | 3.39E-05 | 1.089 |  |
| 260 |  | Nitric oxide signaling in the cardiovascular system | 3.55E-05 | -2.197 |  |
| 261 |  | UVB-induced MAPK signaling | 3.55E-05 | -1.091 |  |
| 262 |  | Fc epsilon RI signaling | 3.55E-05 | -1.061 |  |
| 263 |  | Role of osteoclasts in rheumatoid arthritis signaling pathway | 3.63E-05 | 1.732 |  |
| 264 |  | FLT3 signaling in hematopoietic progenitor cells | 3.80E-05 | -1.177 |  |
| 265 |  | BEX2 signaling pathway | 3.80E-05 | 0.577 |  |
| 266 |  | WNT/SHH axonal guidance signaling pathway | 3.89E-05 | 1.508 |  |
| 267 |  | Leptin signaling in obesity | 3.89E-05 | -1.069 |  |
| 268 |  | Signaling by NOTCH4 | 3.89E-05 | 0.457 |  |
| 269 |  | Pyridoxal 5'-phosphate salvage pathway | 3.98E-05 | -0.426 |  |
| 270 |  | Induction of apoptosis by HIV1 | 3.98E-05 | 0.209 |  |
| 271 |  | Cereblon and thalidomide signaling pathway | 4.17E-05 | 0.429 |  |
| 272 |  | PPAR signaling | 4.37E-05 | -3.053 |  |
| 273 |  | Necroptosis signaling pathway | 4.37E-05 | 0.603 |  |
| 274 |  | Myocardin signaling pathway | 4.57E-05 | -0.822 |  |
| 275 |  | Intrinsic pathway for apoptosis | 4.79E-05 | 0.000 |  |
| 276 |  | Aldosterone signaling in epithelial cells | 4.90E-05 | -2.600 |  |
| 277 |  | Salvage pathways of pyrimidine ribonucleotides | 4.90E-05 | -0.378 |  |
| 278 | 37 | IL-2 signaling | 5.13E-05 | -1.091 |  |
| 279 |  | Protein sorting signaling pathway | 5.25E-05 | -1.021 |  |
| 280 |  | Nucleotide excision repair | 5.25E-05 | 0.507 |  |
| 281 |  | Unfolded protein response | 5.50E-05 | 3.578 |  |
| 282 |  | Hedgehog 'on' state | 5.50E-05 | 0.152 |  |
| 283 |  | Hepatic fibrosis/hepatic stellate cell activation | 5.75E-05 | NaN |  |
| 284 |  | S phase | 6.17E-05 | 0.870 |  |
| 285 | 38 | 4-1BB signaling in T lymphocytes | 6.46E-05 | 2.333 |  |
| 286 |  | Transcriptional regulation by E2F6 | 6.46E-05 | 2.000 | X |
| 287 |  | Fatty acid β-oxidation I | 6.46E-05 | -1.941 | X |
| 288 | 39 | Macrophage classical activation signaling pathway | 6.46E-05 | 1.896 |  |
| 289 |  | Serotonin receptor signaling | 6.92E-05 | -2.376 |  |
| 290 |  | HOTAIR regulatory pathway | 6.92E-05 | 0.149 |  |
| 291 |  | 14-3-3-mediated signaling | 7.24E-05 | -0.539 |  |
| 292 |  | Neuropathic pain signaling in dorsal horn neurons | 7.41E-05 | -3.182 |  |
| 293 |  | Adrenergic receptor signaling pathway (enhanced) | 7.76E-05 | -4.341 |  |
| 294 |  | Role of JAK family kinases in IL-6-type cytokine signaling | 8.13E-05 | 2.746 |  |
| 295 |  | PIP3 activates AKT signaling | 9.12E-05 | -0.762 |  |
| 296 |  | Tight junction signaling | 9.33E-05 | NaN |  |
| 297 |  | mRNA 3 prime end processing signaling pathway | 9.33E-05 | 0.408 | X |
| 298 |  | BAG2 signaling pathway | 9.77E-05 | 0.775 |  |
| 299 |  | Metabolism of water-soluble vitamins and cofactors | 1.02E-04 | -1.134 | X |
| 300 |  | SUMOylation of DNA replication proteins | 1.02E-04 | -0.229 | X |
| 301 |  | Beta-catenin independent WNT signaling | 1.05E-04 | -0.405 |  |
| 302 |  | ISG15 antiviral mechanism | 1.12E-04 | 1.961 |  |
| 303 |  | FOXO-mediated transcription of cell death genes | 1.12E-04 | -0.632 | X |
| 304 |  | Kinesins | 1.17E-04 | -4.379 |  |
| 305 | 40 | IL-15 production | 1.17E-04 | 1.183 |  |
| 306 |  | Cardiac hypertrophy signaling | 1.29E-04 | -2.782 |  |
| 307 |  | HDR through homologous recombination (HRR) or single strand annealing (SSA) | 1.38E-04 | -4.003 | X |
| 308 |  | C-type lectin receptors (CLRs) | 1.41E-04 | 2.469 |  |
| 309 |  | Degradation of beta-catenin by the destruction complex | 1.41E-04 | 1.095 |  |
| 310 |  | Role of p14/p19ARF in tumor suppression | 1.45E-04 | 0.832 | X |
| 311 |  | Cerebral malformation signaling pathway | 1.45E-04 | 0.539 |  |
| 312 |  | PTEN regulation | 1.48E-04 | 2.592 |  |
| 313 |  | Co-stimulation by ICOS | 1.48E-04 | -2.121 | X |
| 314 |  | Glycogen degradation II | 1.48E-04 | -1.414 |  |
| 315 |  | Acetylcholine receptor signaling pathway | 1.51E-04 | -2.828 |  |
| 316 |  | Semaphorin signaling in neurons | 1.55E-04 | NaN |  |
| 317 |  | Sleep NREM signaling pathway | 1.66E-04 | 1.768 | X |
| 318 |  | Apoptosis signaling | 1.66E-04 | 1.095 |  |
| 319 |  | Unfolded protein response (UPR) | 1.78E-04 | 1.291 |  |
| 320 |  | MAPK targets/nuclear events mediated by MAP kinases | 1.78E-04 | 1.291 |  |
| 321 |  | Glycerophospholipid biosynthesis | 1.78E-04 | 0.649 | X |
| 322 |  | Clathrin-mediated endocytosis | 1.78E-04 | 0.000 |  |
| 323 |  | Role of NFAT in cardiac hypertrophy | 1.82E-04 | -3.857 |  |
| 324 |  | NAP1L1 transcription regulation signaling pathway | 1.86E-04 | -1.732 | X |
| 325 |  | Glutaminergic receptor signaling pathway (enhanced) | 1.91E-04 | -1.627 |  |
| 326 |  | IL-17A signaling in airway cells | 1.91E-04 | -1.528 |  |
| 327 |  | Cytosolic sensors of pathogen-associated DNA | 1.95E-04 | 1.460 |  |
| 328 |  | Semaphorin interactions | 1.95E-04 | 1.460 |  |
| 329 |  | Deubiquitination | 2.19E-04 | 2.151 |  |
| 330 |  | Macroautophagy | 2.45E-04 | -2.041 | X |
| 331 |  | Estrogen receptor signaling | 2.57E-04 | -1.767 |  |
| 332 | 41 | iNOS signaling | 2.63E-04 | 1.291 |  |
| 333 |  | Role of JAK1, JAK2 and TYK2 in interferon signaling | 2.69E-04 | -1.633 |  |
| 334 |  | ATF4 activates genes in response to endoplasmic reticulum stress | 2.69E-04 | 0.832 | X |
| 335 |  | Hypoxia signaling in the cardiovascular system | 2.82E-04 | 1.508 |  |
| 336 |  | Signaling by NOTCH1 | 2.82E-04 | 1.177 |  |
| 337 |  | SUMOylation of DNA damage response and repair proteins | 2.82E-04 | -0.784 | X |
| 338 |  | Xenobiotic metabolism signaling | 2.88E-04 | NaN |  |
| 339 | 42 | Clathrin-mediated endocytosis signaling | 2.88E-04 | NaN | X |
| 340 |  | ISGylation signaling pathway | 2.88E-04 | -0.870 |  |
| 341 |  | Fanconi anemia pathway | 2.95E-04 | -1.500 | X |
| 342 |  | Thioredoxin signaling pathway | 2.95E-04 | 1.000 |  |
| 343 |  | FXR/RXR activation | 3.16E-04 | -3.111 |  |
| 344 |  | Endothelin-1 signaling | 3.16E-04 | -2.359 |  |
| 345 |  | Role of osteoblasts in rheumatoid arthritis signaling pathway | 3.24E-04 | 4.202 |  |
| 346 |  | α-adrenergic signaling | 3.39E-04 | -2.828 |  |
| 347 |  | Cellular response to heat stress | 3.39E-04 | 2.335 | X |
| 348 |  | DNA damage-induced 14-3-3σ signaling | 3.39E-04 | -1.500 | X |
| 349 |  | Superpathway of serine and glycine biosynthesis I | 3.39E-04 | 1.342 | X |
| 350 |  | Melanoma signaling | 3.47E-04 | -1.886 | X |
| 351 |  | Branched-chain amino acid catabolism | 3.55E-04 | -1.508 | X |
| 352 |  | Oxytocin signaling pathway | 3.63E-04 | -1.477 |  |
| 353 |  | Neuroinflammation signaling pathway | 3.80E-04 | -0.630 |  |
| 354 |  | Gα12/13 signaling | 3.98E-04 | -1.616 |  |
| 355 |  | MAPK6/MAPK4 signaling | 4.07E-04 | 2.043 |  |
| 356 |  | Pyroptosis signaling pathway | 4.07E-04 | 1.800 |  |
| 357 |  | Ribavirin ADME | 4.07E-04 | -1.460 |  |
| 358 |  | MAP kinase activation | 4.17E-04 | 2.000 |  |
| 359 |  | Breast cancer regulation by stathmin1 | 4.17E-04 | -0.642 |  |
| 360 |  | Co-inhibition by BTLA | 4.27E-04 | -1.279 | X |
| 361 |  | Interferon gamma signaling | 4.47E-04 | -1.095 |  |
| 362 |  | Signaling by NTRK1 (TRKA) | 5.25E-04 | 0.784 |  |
| 363 |  | DDX58/IFIH1-mediated induction of interferon-alpha/beta | 5.25E-04 | 0.392 | X |
| 364 |  | Regulation of mRNA stability by proteins that bind AU-rich elements | 5.37E-04 | 3.780 |  |
| 365 |  | Opioid signaling | 5.37E-04 | -3.402 | X |
| 366 |  | ERB2-ERBB3 signaling | 5.50E-04 | -0.894 | X |
| 367 |  | Metabolism of polyamines | 5.62E-04 | 3.710 |  |
| 368 |  | Antiproliferative role of somatostatin receptor 2 | 5.62E-04 | -1.789 | X |
| 369 |  | Netrin signaling | 5.62E-04 | -1.180 |  |
| 370 |  | Endometrial cancer signaling | 5.62E-04 | -0.447 |  |
| 371 |  | Deadenylation-dependent mRNA decay | 5.75E-04 | 2.683 |  |
| 372 |  | Somitogenesis | 5.75E-04 | 2.236 |  |
| 373 |  | CNTF signaling | 5.75E-04 | -1.342 | X |
| 374 |  | NLR signaling pathways | 5.75E-04 | 0.447 |  |
| 375 |  | Response of EIF2AK1 (HRI) to heme deficiency | 5.89E-04 | 2.496 |  |
| 376 |  | TBC/RABGAPs | 5.89E-04 | -2.183 |  |
| 377 |  | DNA damage bypass | 5.89E-04 | -1.886 |  |
| 378 |  | ABC-family proteins mediated transport | 5.89E-04 | 1.715 |  |
| 379 |  | Energy dependent regulation of mTOR by LKB1-AMPK | 5.89E-04 | -0.832 | X |
| 380 |  | HEY1 signaling pathway | 6.03E-04 | -0.926 |  |
| 381 |  | MHC class II antigen presentation | 6.46E-04 | -2.667 |  |
| 382 |  | Mismatch repair in eukaryotes | 6.46E-04 | -1.897 | X |
| 383 |  | S-adenosylmethionine regulatory signaling pathway | 6.46E-04 | 1.732 |  |
| 384 |  | γ-linolenate biosynthesis II (animals) | 6.46E-04 | -1.000 | X |
| 385 |  | Thyroid cancer signaling | 6.76E-04 | -2.200 |  |
| 386 |  | VDR/RXR activation | 6.76E-04 | 0.943 |  |
| 387 |  | NIK-->noncanonical NF-kB signaling | 6.92E-04 | 3.710 |  |
| 388 |  | Cholesterol biosynthesis I | 6.92E-04 | -2.121 | X |
| 389 |  | Cholesterol biosynthesis II (via 24,25-dihydrolanosterol) | 6.92E-04 | -2.121 | X |
| 390 |  | Cholesterol biosynthesis III (via desmosterol) | 6.92E-04 | -2.121 | X |
| 391 | 43 | Granzyme B signaling | 6.92E-04 | -1.890 |  |
| 392 |  | Glycogen degradation III | 6.92E-04 | -1.414 |  |
| 393 |  | Bladder cancer signaling | 6.92E-04 | -0.333 |  |
| 394 |  | Sperm motility | 7.24E-04 | -1.569 |  |
| 395 |  | ERK5 signaling | 7.24E-04 | 1.279 |  |
| 396 |  | Apelin cardiomyocyte signaling pathway | 7.41E-04 | -2.414 |  |
| 397 |  | RNA polymerase III transcription | 7.41E-04 | 2.000 | X |
| 398 |  | Hedgehog 'off' state | 7.59E-04 | -0.522 |  |
| 399 |  | Stearate biosynthesis I (animals) | 7.76E-04 | 0.277 | X |
| 400 |  | Synaptic long term potentiation | 8.32E-04 | -3.536 |  |
| 401 |  | Superpathway of cholesterol biosynthesis | 8.32E-04 | -2.309 | X |
| 402 |  | NRF2-mediated oxidative stress response | 8.51E-04 | 1.000 |  |
| 403 |  | RAF-independent MAPK1/3 activation | 8.71E-04 | 0.905 |  |
| 404 |  | ERBB signaling | 9.12E-04 | -0.962 |  |
| 405 |  | Sphingolipid metabolism | 9.33E-04 | -1.257 |  |
| 406 |  | NFE2L2 regulating MDR associated enzymes | 9.33E-04 | 0.000 |  |
| 407 |  | TWEAK signaling | 9.77E-04 | -0.277 |  |
| 408 |  | Androgen signaling | 1.02E-03 | -2.985 | X |
| 409 |  | Role of PI3K/AKT signaling in the pathogenesis of influenza | 1.02E-03 | -2.065 |  |
| 410 |  | Transcriptional regulation by RUNX1 | 1.02E-03 | 1.155 |  |
| 411 |  | GPER1 signaling | 1.05E-03 | -3.286 | X |
| 412 |  | Cholecystokinin/gastrin-mediated signaling | 1.05E-03 | 0.707 |  |
| 413 |  | Gαs signaling | 1.12E-03 | -1.732 | X |
| 414 |  | Glucocorticoid receptor signaling | 1.15E-03 | NaN |  |
| 415 |  | Dopamine receptor signaling | 1.17E-03 | -3.162 |  |
| 416 |  | Metabolism of folate and pterines | 1.17E-03 | 1.414 |  |
| 417 |  | Maternal to zygotic transition signaling pathway | 1.17E-03 | 1.151 | X |
| 418 |  | FOXO-mediated transcription of cell cycle genes | 1.17E-03 | -1.000 | X |
| 419 |  | Polyamine regulation in colon cancer | 1.17E-03 | 0.943 | X |
| 420 |  | SUMOylation of DNA methylation proteins | 1.17E-03 | -0.333 | X |
| 421 |  | Nuclear RNA decay | 1.26E-03 | 2.673 | X |
| 422 |  | DNA double-strand break repair by non-homologous end joining | 1.26E-03 | -1.414 | X |
| 423 |  | Cell cycle: G2/M DNA damage checkpoint regulation | 1.26E-03 | 1.291 | X |
| 424 |  | Glucose metabolism | 1.29E-03 | 1.569 | X |
| 425 |  | VEGF family ligand-receptor interactions | 1.29E-03 | -1.400 |  |
| 426 | 44 | Role of RIG1-like receptors in antiviral innate immunity | 1.29E-03 | 1.265 | X |
| 427 | 45 | Toll-like receptor signaling | 1.29E-03 | 1.155 |  |
| 428 |  | Signaling by SCF-KIT | 1.32E-03 | 1.000 |  |
| 429 |  | Peroxisomal protein import | 1.35E-03 | -3.273 | X |
| 430 |  | Actin nucleation by ARP-WASP complex | 1.41E-03 | 0.243 |  |
| 431 |  | EGF signaling | 1.51E-03 | -1.414 |  |
| 432 |  | MTOR signaling | 1.51E-03 | 1.155 | X |
| 433 |  | Sulfur amino acid metabolism | 1.51E-03 | 0.577 | X |
| 434 |  | Intra-golgi and retrograde golgi-to-ER traffic | 1.55E-03 | -2.263 |  |
| 435 |  | Neurotrophin/TRK signaling | 1.55E-03 | -1.877 |  |
| 436 |  | Renal cell carcinoma signaling | 1.55E-03 | -1.213 |  |
| 437 |  | XIST epigenetic regulation signaling pathway | 1.58E-03 | 2.214 |  |
| 438 |  | Endoplasmic reticulum stress pathway | 1.58E-03 | 1.897 |  |
| 439 |  | Cellular effects of Sildenafil (Viagra) | 1.62E-03 | -0.522 |  |
| 440 |  | Circadian rhythm signaling | 1.66E-03 | NaN |  |
| 441 |  | SUMOylation of RNA binding proteins | 1.66E-03 | 1.213 | X |
| 442 |  | RHOA signaling | 1.66E-03 | -0.365 | X |
| 443 |  | TP53 regulates transcription of cell death genes | 1.70E-03 | -1.500 |  |
| 444 |  | Transcriptional regulation by the AP-2 (TFAP2) family of transcription factors | 1.70E-03 | 1.387 | X |
| 445 |  | Chemokine signaling | 1.70E-03 | -0.626 | X |
| 446 |  | Cohesin chromatin regulation pathway | 1.74E-03 | -2.066 | X |
| 447 |  | Human embryonic stem cell pluripotency | 1.74E-03 | 1.571 |  |
| 448 |  | Hematoma resolution signaling pathway | 1.95E-03 | -2.994 |  |
| 449 | 46 | Fcγ receptor-mediated phagocytosis in macrophages and monocytes | 1.95E-03 | -1.732 | X |
| 450 |  | TR/RXR activation | 1.95E-03 | -0.845 | X |
| 451 |  | Acute phase response signaling | 2.00E-03 | 2.214 |  |
| 452 |  | Ephrin A signaling | 2.00E-03 | -0.493 |  |
| 453 |  | Melatonin signaling | 2.04E-03 | -1.964 |  |
| 454 |  | Mitochondrial protein degradation | 2.04E-03 | 1.890 |  |
| 455 | 47 | PFKFB4 signaling pathway | 2.09E-03 | -2.000 | X |
| 456 |  | Nicotinate metabolism | 2.09E-03 | -0.577 | X |
| 457 | 48 | MSP-RON signaling pathway | 2.19E-03 | NaN |  |
| 458 |  | Leucine degradation I | 2.19E-03 | -1.633 | X |
| 459 |  | Lipophagy | 2.19E-03 | -0.816 | X |
| 460 |  | Mitochondrial fatty acid beta-oxidation | 2.29E-03 | -2.673 | X |
| 461 |  | Regulation of actin-based motility by Rho | 2.29E-03 | 0.000 |  |
| 462 |  | Antioxidant action of vitamin C | 2.40E-03 | -1.606 |  |
| 463 |  | Abacavir ADME | 2.45E-03 | -2.400 | X |
| 464 |  | L1CAM interactions | 2.45E-03 | -0.522 |  |
| 465 |  | Parkinson's signaling pathway | 2.51E-03 | 2.109 |  |
| 466 |  | Egg-sperm fusion signaling pathway | 2.57E-03 | -2.556 | X |
| 467 |  | Regulation of the epithelial-mesenchymal transition pathway | 2.75E-03 | NaN |  |
| 468 | 49 | p38 MAPK signaling | 2.82E-03 | 1.732 |  |
| 469 |  | M-decay: degradation of maternal mRNAs by maternally stored factors | 2.82E-03 | 1.500 | X |
| 470 |  | Role of IL-17F in allergic inflammatory airway diseases | 2.82E-03 | 0.832 |  |
| 471 |  | Transcriptional regulatory network in embryonic stem cells | 2.88E-03 | 0.632 |  |
| 472 |  | Modulation of host responses by IFN-stimulated genes | 2.95E-03 | 1.000 | X |
| 473 |  | Mitochondrial L-carnitine shuttle pathway | 2.95E-03 | -0.707 | X |
| 474 |  | Transcriptional regulation by RUNX2 | 3.39E-03 | 0.728 |  |
| 475 |  | NFE2L2 regulating anti-oxidant/detoxification enzymes | 3.55E-03 | 1.897 | X |
| 476 |  | PKR-mediated signaling | 3.55E-03 | 0.392 | X |
| 477 |  | Rap1 signaling | 3.63E-03 | -2.828 |  |
| 478 |  | Mitotic prophase | 3.72E-03 | -0.333 |  |
| 479 |  | Cholesterol biosynthesis | 3.89E-03 | -2.714 | X |
| 480 |  | Effects of PIP2 hydrolysis | 3.89E-03 | -0.905 | X |
| 481 |  | Chromatin modifying enzymes | 3.89E-03 | -0.128 | X |
| 482 |  | ABRA signaling pathway | 3.98E-03 | 1.800 |  |
| 483 |  | Role of JAK2 in hormone-like cytokine signaling | 3.98E-03 | 1.213 | X |
| 484 |  | GPVI-mediated activation cascade | 3.98E-03 | -0.277 | X |
| 485 |  | Signaling by CSF1 (M-CSF) in myeloid cells | 3.98E-03 | 0.000 | X |
| 486 |  | Response to elevated platelet cytosolic Ca2+ | 4.17E-03 | 1.372 |  |
| 487 | 50 | TREM1 signaling | 4.17E-03 | 1.147 |  |
| 488 | 51 | Crosstalk between dendritic cells and natural killer cells | 4.27E-03 | 2.400 |  |
| 489 |  | Gamma carboxylation, hypusinylation, hydroxylation, and arylsulfatase activation | 4.37E-03 | 2.524 | X |
| 490 |  | ATF6 (ATF6-alpha) activates chaperone genes | 4.37E-03 | 2.449 |  |
| 491 |  | Transcriptional regulation of white adipocyte differentiation | 4.37E-03 | 1.225 | X |
| 492 |  | Valine degradation I | 4.47E-03 | -0.707 | X |
| 493 |  | Signaling by Hippo | 4.47E-03 | 0.333 | X |
| 494 |  | GP6 signaling pathway | 4.68E-03 | -1.134 | X |
| 495 |  | Extra-nuclear estrogen signaling | 4.68E-03 | -0.853 |  |
| 496 |  | Dopamine-DARPP32 feedback in cAMP signaling | 4.90E-03 | -2.333 |  |
| 497 |  | Regulation of RUNX1 expression and activity | 5.01E-03 | 1.265 | X |
| 498 | 52 | Tumoricidal function of hepatic natural killer cells | 5.01E-03 | 1.134 |  |
| 499 |  | Integrin cell surface interactions | 5.13E-03 | -0.816 |  |
| 500 |  | TP53 regulates metabolic genes | 5.13E-03 | 0.408 |  |
| 501 |  | Degradation of CRY and PER proteins | 5.25E-03 | 3.441 |  |
| 502 |  | Telomere maintenance | 5.25E-03 | -2.121 |  |
| 503 |  | Centrosomal KIAA0586 signaling pathway | 5.37E-03 | 1.567 | X |
| 504 |  | RAB GEFs exchange GTP for GDP on RABs | 5.37E-03 | -0.200 | X |
| 505 |  | ERBB4 signaling | 5.75E-03 | -2.357 | X |
| 506 |  | Cargo recognition for clathrin-mediated endocytosis | 5.75E-03 | 0.756 | X |
| 507 |  | MITF-M-dependent gene expression | 5.89E-03 | -3.157 | X |
| 508 |  | Corticotropin releasing hormone signaling | 5.89E-03 | -1.512 |  |
| 509 |  | tRNA splicing | 6.03E-03 | -2.496 | X |
| 510 |  | Interleukin-10 signaling | 6.03E-03 | 2.324 |  |
| 511 |  | DAG and IP3 signaling | 6.46E-03 | -2.673 | X |
| 512 |  | 2-oxobutanoate degradation I | 6.46E-03 | -1.000 | X |
| 513 |  | Interferon alpha/beta signaling | 6.46E-03 | -0.853 |  |
| 514 |  | dTMP de novo biosynthesis | 6.46E-03 | 0.000 | X |
| 515 |  | Vitamin K signaling pathway | 6.76E-03 | -1.789 |  |
| 516 |  | Signaling by NOTCH3 | 6.92E-03 | 0.500 |  |
| 517 |  | Signaling by TGFBR3 | 6.92E-03 | 0.000 |  |
| 518 | 53 | MIF regulation of innate immunity | 7.59E-03 | 2.496 |  |
| 519 |  | Transport of inorganic cations/anions and amino acids/oligopeptides | 7.59E-03 | 1.134 |  |
| 520 |  | Role of OCT4 in mammalian embryonic stem cell pluripotency | 7.59E-03 | 0.447 | X |
| 521 |  | Amino acids regulate mTORC1 | 7.76E-03 | -0.243 | X |
| 522 |  | NFE2L2 regulating tumorigenic genes | 7.94E-03 | 1.633 |  |
| 523 |  | Metabolism of non-coding RNA | 8.32E-03 | 3.300 | X |
| 524 |  | Signaling by the B cell receptor (BCR) | 8.32E-03 | 2.967 |  |
| 525 | 54 | CCR3 signaling in eosinophils | 8.32E-03 | -1.964 | X |
| 526 |  | Miro GTPase cycle | 8.32E-03 | 1.342 | X |
| 527 |  | Regulation of NFE2L2 gene expression | 8.32E-03 | 1.342 | X |
| 528 |  | UVC-induced MAPK signaling | 8.32E-03 | -1.291 |  |
| 529 |  | Sphingomyelin metabolism | 8.32E-03 | -1.000 |  |
| 530 |  | Transcriptional activity of SMAD2/SMAD3:SMAD4 heterotrimer | 8.32E-03 | 1.000 |  |
| 531 |  | Meiosis initiation signaling pathway | 8.32E-03 | -0.784 |  |
| 532 |  | Mitochondrial unfolded protein response (UPRmt) | 8.32E-03 | 0.707 |  |
| 533 |  | WNK renal signaling pathway | 8.51E-03 | -2.132 | X |
| 534 |  | Mouse embryonic stem cell pluripotency | 8.51E-03 | -1.400 |  |
| 535 |  | Regulation of TP53 expression and degradation | 8.51E-03 | -1.387 |  |
| 536 |  | Mitochondrial protein import | 8.91E-03 | 3.441 |  |
| 537 |  | Serine biosynthesis | 9.12E-03 | NaN | X |
| 538 |  | 5-aminoimidazole ribonucleotide biosynthesis I | 9.12E-03 | NaN |  |
| 539 | 55 | FAT10 signaling pathway | 9.12E-03 | 1.342 |  |
| 540 |  | Signaling by CSF3 (G-CSF) | 9.12E-03 | 0.905 | X |
| 541 |  | Cardiac β-adrenergic signaling | 9.33E-03 | -3.024 | X |
| 542 |  | Glycogen metabolism | 9.33E-03 | -2.530 | X |
| 543 |  | Regulation of lipid metabolism by PPARalpha | 9.55E-03 | 1.095 | X |
| 544 |  | EIF2 signaling | 9.55E-03 | -0.522 |  |
| 545 |  | Triacylglycerol biosynthesis | 9.77E-03 | 1.732 |  |
| 546 |  | Activation of NMDA receptors and postsynaptic events | 1.05E-02 | -1.877 | X |
| 547 |  | Fatty acid activation | 1.05E-02 | 0.000 | X |
| 548 |  | Dilated cardiomyopathy signaling pathway | 1.12E-02 | -2.268 | X |
| 549 |  | Oncogene induced senescence | 1.15E-02 | 0.577 |  |
| 550 |  | Gαi signaling | 1.15E-02 | -0.378 | X |
| 551 |  | FGF signaling | 1.20E-02 | -1.528 |  |
| 552 |  | RHO GTPases activate CIT | 1.20E-02 | -1.414 | X |
| 553 |  | Sonic hedgehog signaling | 1.20E-02 | 1.414 | X |
| 554 |  | Platelet homeostasis | 1.20E-02 | -0.626 |  |
| 555 |  | Differential regulation of cytokine production in intestinal epithelial cells by IL-17A and IL-17F | 1.23E-02 | 3.000 |  |
| 556 |  | Fc epsilon receptor (FCERI) signaling | 1.23E-02 | 1.896 |  |
| 557 |  | NAD salvage pathway II | 1.23E-02 | -1.890 | X |
| 558 |  | SPINK1 general cancer pathway | 1.23E-02 | -1.886 |  |
| 559 |  | Syndecan interactions | 1.23E-02 | 0.000 |  |
| 560 |  | Assembly of RNA polymerase I complex | 1.29E-02 | 2.449 |  |
| 561 |  | HDR through MMEJ (alt-NHEJ) | 1.29E-02 | -1.633 | X |
| 562 |  | Z-decay: degradation of maternal mRNAs by zygotically expressed factors | 1.29E-02 | 1.633 |  |
| 563 |  | Signaling by leptin | 1.29E-02 | 0.816 | X |
| 564 |  | Cellular response to hypoxia | 1.32E-02 | 2.400 |  |
| 565 |  | Cancer drug resistance by drug efflux | 1.32E-02 | -2.324 |  |
| 566 |  | Co-stimulation by CD28 | 1.35E-02 | -1.291 | X |
| 567 |  | DHCR24 signaling pathway | 1.41E-02 | -1.372 |  |
| 568 |  | Xenobiotic metabolism general signaling pathway | 1.45E-02 | -1.808 |  |
| 569 |  | Signaling by TGF-beta receptor complex | 1.45E-02 | 0.500 |  |
| 570 |  | Regulation of RUNX2 expression and activity | 1.48E-02 | 3.578 |  |
| 571 |  | OAS antiviral response | 1.48E-02 | -0.447 | X |
| 572 |  | UDP-N-acetyl-D-glucosamine biosynthesis II | 1.51E-02 | 2.000 | X |
| 573 |  | Glycine cleavage complex | 1.51E-02 | 1.000 | X |
| 574 |  | Signal regulatory protein family interactions | 1.51E-02 | 0.378 | X |
| 575 |  | Pyrimidine ribonucleotides interconversion | 1.62E-02 | -1.667 | X |
| 576 |  | Vitamin-C transport | 1.66E-02 | 1.890 | X |
| 577 |  | Airway pathology in chronic obstructive pulmonary disease | 1.74E-02 | NaN |  |
| 578 | 56 | Airway Inflammation in asthma | 1.95E-02 | 1.342 |  |
| 579 |  | Oleate biosynthesis II (animals) | 2.00E-02 | -2.236 | X |
| 580 |  | Activation of gene expression by SREBF (SREBP) | 2.00E-02 | -1.941 | X |
| 581 |  | Trans-golgi network vesicle budding | 2.00E-02 | 1.342 |  |
| 582 |  | Formation of WDR5-containing histone-modifying complexes | 2.00E-02 | -0.832 | X |
| 583 |  | G alpha (12/13) signaling events | 2.04E-02 | -1.528 |  |
| 584 |  | Apelin adipocyte signaling pathway | 2.04E-02 | 0.471 | X |
| 585 |  | WNT/β-catenin signaling | 2.04E-02 | 0.354 |  |
| 586 |  | RAN signaling | 2.19E-02 | 2.646 | X |
| 587 |  | Metabolism of cofactors | 2.19E-02 | 0.333 | X |
| 588 |  | Growth hormone receptor signaling | 2.19E-02 | 0.333 | X |
| 589 | 57 | MIF-mediated glucocorticoid regulation | 2.24E-02 | 2.530 |  |
| 590 |  | Plasma lipoprotein assembly, remodeling, and clearance | 2.29E-02 | -0.894 |  |
| 591 |  | Proteasome assembly | 2.34E-02 | 2.840 |  |
| 592 |  | Amyloid processing | 2.34E-02 | -1.633 | X |
| 593 |  | Amyotrophic lateral sclerosis signaling | 2.45E-02 | -1.400 |  |
| 594 |  | Smooth muscle contraction | 2.45E-02 | -0.277 | X |
| 595 |  | Prostanoid biosynthesis | 2.51E-02 | NaN | X |
| 596 |  | RUNX1 and FOXP3 control the development of regulatory T lymphocytes (Tregs) | 2.51E-02 | -0.447 | X |
| 597 |  | mRNA editing | 2.51E-02 | 0.447 | X |
| 598 |  | Mitochondrial dysfunction | 2.57E-02 | -1.364 |  |
| 599 |  | Translocation of SLC2A4 (GLUT4) to the plasma membrane | 2.63E-02 | -2.524 | X |
| 600 |  | HIPPO signaling | 2.63E-02 | -0.688 | X |
| 601 |  | Inositol phosphate metabolism | 2.63E-02 | -0.535 | X |
| 602 |  | Neuropeptides in migraine signaling pathway | 2.69E-02 | -1.480 |  |
| 603 |  | Neurotransmitter uptake and metabolism in glial cells | 2.82E-02 | NaN | X |
| 604 |  | Branched-chain α-keto acid dehydrogenase complex | 2.82E-02 | NaN | X |
| 605 |  | Heme biosynthesis from uroporphyrinogen-III I | 2.82E-02 | NaN | X |
| 606 |  | Methylmalonyl pathway | 2.82E-02 | NaN | X |
| 607 |  | Proline biosynthesis I | 2.82E-02 | NaN |  |
| 608 |  | Orexin signaling pathway | 2.88E-02 | -3.753 |  |
| 609 |  | TYSND1 cleaves peroxisomal proteins | 2.88E-02 | -2.000 | X |
| 610 |  | Pyrimidine ribonucleotides de novo biosynthesis | 2.88E-02 | -1.667 | X |
| 611 |  | TAK1-dependent IKK and NF-kappa-B activation | 2.88E-02 | 0.832 |  |
| 612 |  | Colanic acid building blocks biosynthesis | 2.88E-02 | -0.816 | X |
| 613 |  | Carnitine shuttle | 2.88E-02 | 0.000 | X |
| 614 |  | Aryl hydrocarbon receptor signaling | 2.88E-02 | 0.000 | X |
| 615 |  | Epithelial-mesenchymal transition (EMT) during gastrulation | 2.88E-02 | 0.000 | X |
| 616 |  | Inositol pyrophosphates biosynthesis | 2.88E-02 | 0.000 | X |
| 617 |  | Differential regulation of cytokine production in macrophages and T helper cells by IL-17A and IL-17F | 2.95E-02 | 2.646 |  |
| 618 |  | Superpathway of methionine degradation | 2.95E-02 | 0.905 | X |
| 619 |  | Formation of paraxial mesoderm | 2.95E-02 | -0.707 |  |
| 620 |  | SUMOylation of chromatin organization proteins | 2.95E-02 | 0.688 | X |
| 621 |  | Transcriptional regulation by NPAS4 | 2.95E-02 | 0.302 |  |
| 622 |  | Phosphorylated BMAL1:CLOCK (ARNTL:CLOCK) activates expression of core clock genes | 2.95E-02 | 0.000 |  |
| 623 |  | Synaptogenesis signaling pathway | 3.09E-02 | -1.838 |  |
| 624 |  | Regulation of apoptosis | 3.16E-02 | 2.840 |  |
| 625 |  | Signaling by ALK | 3.24E-02 | -0.632 | X |
| 626 |  | Mitophagy | 3.24E-02 | 0.577 | X |
| 627 |  | Signaling by MET | 3.31E-02 | 1.789 |  |
| 628 |  | SUMOylation of transcription cofactors | 3.39E-02 | -0.277 | X |
| 629 |  | Integrin signaling | 3.55E-02 | -1.667 |  |
| 630 |  | NF1 RAS signaling pathway | 3.55E-02 | -0.990 |  |
| 631 |  | Signaling by NOTCH2 | 3.55E-02 | 0.905 |  |
| 632 |  | DNA replication pre-initiation | 3.55E-02 | -0.707 | X |
| 633 |  | Tryptophan degradation III (eukaryotic) | 3.80E-02 | -2.449 | X |
| 634 |  | NAD phosphorylation and dephosphorylation | 3.80E-02 | -2.236 | X |
| 635 | 58 | S100 family signaling pathway | 3.80E-02 | -1.794 |  |
| 636 |  | Apelin cardiac fibroblast signaling pathway | 3.80E-02 | -1.414 | X |
| 637 |  | FOXO-mediated transcription | 3.98E-02 | -1.890 | X |
| 638 |  | Adipogenesis pathway | 3.98E-02 | 1.461 |  |
| 639 |  | RIPK1-mediated regulated necrosis | 3.98E-02 | 1.265 | X |
| 640 |  | Synthesis of prostaglandins (PG) and thromboxanes (TX) | 3.98E-02 | -0.816 | X |
| 641 |  | Mitotic telophase/cytokinesis | 3.98E-02 | -0.816 |  |
| 642 |  | Interleukin-15 signaling | 3.98E-02 | 0.816 | X |
| 643 |  | RHO GTPases activate ROCKs | 3.98E-02 | 0.378 | X |
| 644 |  | Regulation of the epithelial mesenchymal transition in development pathway | 4.17E-02 | 1.807 |  |
| 645 |  | Nucleotide excision repair pathway | 4.27E-02 | NaN | X |
| 646 |  | GDNF family ligand-receptor interactions | 4.27E-02 | -2.183 | X |
| 647 |  | Fatty acyl-CoA biosynthesis | 4.27E-02 | 0.302 | X |
| 648 |  | Gene and protein expression by JAK-STAT signaling after IL-12 stimulation | 4.27E-02 | 0.302 | X |
| 649 |  | RNA polymerase II transcription | 4.37E-02 | 4.700 | X |
| 650 |  | TGF-β signaling | 4.47E-02 | 0.218 |  |
| 651 |  | BBSome signaling pathway | 4.57E-02 | -2.871 | X |
| 652 |  | ROBO SLIT signaling pathway | 4.57E-02 | -1.512 |  |
| 653 |  | Role of hypercytokinemia/hyperchemokinemia in the pathogenesis of influenza | 4.57E-02 | 0.447 |  |
| 654 |  | UDP-D-xylose and UDP-D-glucuronate biosynthesis | 4.68E-02 | NaN | X |
| 655 |  | Sulfate activation for sulfonation | 4.68E-02 | NaN | X |
| 656 |  | Glycerol-3-phosphate shuttle | 4.68E-02 | NaN | X |
| 657 |  | Glycine biosynthesis I | 4.68E-02 | NaN | X |
| 658 |  | Integration of energy metabolism | 4.68E-02 | -2.600 | X |
| 659 |  | GPCR-mediated integration of enteroendocrine signaling exemplified by an L cell | 4.68E-02 | -1.000 | X |
| 660 |  | Inhibition of angiogenesis by TSP1 | 4.68E-02 | 0.816 |  |
| 661 |  | Interleukin-6 family signaling | 4.68E-02 | 0.707 |  |
| 662 |  | Apoptotic execution phase | 4.68E-02 | -0.535 |  |
| 663 |  | Factors involved in megakaryocyte development and platelet production | 4.68E-02 | -0.408 | X |
| 664 |  | Endocannabinoid neuronal synapse pathway | 4.68E-02 | -0.200 | X |
| 665 |  | Other interleukin signaling | 4.68E-02 | 0.000 |  |
| 666 | 59 | IL-22 signaling | 4.68E-02 | 0.000 | X |
| 667 |  | TNF signaling | 4.79E-02 | 0.775 | X |

**Supplementary Table 2:** Ingenuity Pathway Analysis® (IPA®) for down- and up-regulated differentially-expressed genes (DEGs) of Tc2 cells compared to cells cultured in IL-2 alone (fold change > |1.5|, P_adjusted_ < 0.05). Ranking of canonical and cellular immune response pathways are displayed based on their overall ranking with a Benjamini-Hochberg (BH) P < 0.05. Unique canonical and cellular immune response pathways of Tc1 compared Tc2 cells are marked (X).

| **Ranking # canonical pathways** | **Ranking # immune pathways** | **Ingenuity canonical pathways** | **BH**  **p-value** | **Z-score** | **Unique pathways**  **in Tc2** |
| --- | --- | --- | --- | --- | --- |
| 1 |  | RHO GTPase cycle | 1.00E-21 | -0.341 |  |
| 2 |  | Chronic myeloid leukemia signaling | 1.26E-15 | -0.965 |  |
| 3 |  | Cardiac hypertrophy signaling (enhanced) | 5.01E-15 | 0.466 |  |
| 4 | 1 | Th1 and Th2 activation pathway | 6.31E-15 | NaN |  |
| 5 |  | rRNA modification in the nucleus and cytosol | 5.01E-13 | 6.000 |  |
| 6 |  | IL-27 signaling pathway | 1.26E-12 | -0.429 |  |
| 7 |  | Cachexia signaling pathway | 2.51E-12 | 2.975 |  |
| 8 | 2 | Th2 pathway | 6.31E-11 | 2.160 |  |
| 9 |  | Protein kinase A signaling | 1.00E-10 | -1.937 |  |
| 10 |  | Molecular mechanisms of cancer | 1.07E-10 | 0.905 |  |
| 11 | 3 | IL-10 signaling | 2.19E-10 | -0.714 |  |
| 12 |  | NAFLD signaling pathway | 2.63E-10 | 3.447 |  |
| 13 |  | Role of tissue factor in cancer | 2.88E-10 | 1.664 |  |
| 14 |  | Hepatic fibrosis signaling pathway | 3.16E-10 | 0.000 |  |
| 15 |  | Glycation signaling pathway | 3.31E-10 | 2.077 |  |
| 16 |  | XBP1(S) activates chaperone genes | 3.72E-10 | 3.922 |  |
| 17 |  | Class I MHC mediated antigen processing and presentation | 5.75E-10 | 1.225 |  |
| 18 | 4 | Cyclophilin signaling pathway | 1.17E-09 | 2.151 |  |
| 19 |  | Pathogen induced cytokine storm signaling pathway | 1.23E-09 | 4.364 |  |
| 20 |  | Role of macrophages, fibroblasts and endothelial cells in rheumatoid arthritis | 1.41E-09 | 2.525 |  |
| 21 |  | Hepatic cholestasis | 2.09E-09 | 2.286 |  |
| 22 | 5 | Interleukin-4 and Interleukin-13 signaling | 2.14E-09 | 2.214 |  |
| 23 |  | Osteoarthritis pathway | 3.39E-09 | 2.832 |  |
| 24 | 6 | Role of pattern recognition receptors in recognition of bacteria and viruses | 3.55E-09 | -1.177 |  |
| 25 | 7 | PD-1, PD-L1 cancer immunotherapy pathway | 3.63E-09 | 0.000 |  |
| 26 |  | Erythropoietin signaling pathway | 6.61E-09 | -2.941 |  |
| 27 |  | Mitotic metaphase and anaphase | 6.61E-09 | -1.723 |  |
| 28 |  | Pancreatic adenocarcinoma signaling | 7.94E-09 | 1.000 |  |
| 29 | 8 | Th1 pathway | 7.94E-09 | 0.333 |  |
| 30 | 9 | Macrophage classical activation signaling pathway | 9.12E-09 | 2.598 |  |
| 31 |  | PI3K/AKT signaling | 1.10E-08 | 0.870 |  |
| 32 |  | CGRP in inflammation and nociception signaling pathway | 1.78E-08 | -2.064 |  |
| 33 |  | Multiple sclerosis signaling pathway | 2.63E-08 | 1.722 |  |
| 34 |  | CDX gastrointestinal cancer signaling pathway | 5.37E-08 | -0.816 |  |
| 35 |  | Colorectal cancer metastasis signaling | 5.37E-08 | 0.788 |  |
| 36 | 10 | Macrophage alternative activation signaling pathway | 6.61E-08 | 2.100 |  |
| 37 |  | Neuroinflammation signaling pathway | 7.41E-08 | 0.384 |  |
| 38 |  | STAT3 pathway | 7.59E-08 | 0.928 |  |
| 39 |  | Prostate cancer signaling | 8.71E-08 | -1.000 |  |
| 40 | 11 | HMGB1 signaling | 9.77E-08 | 1.219 |  |
| 41 |  | Pulmonary hypertension signaling pathway | 9.77E-08 | 0.325 |  |
| 42 |  | Role of osteoblasts, osteoclasts and chondrocytes in rheumatoid arthritis | 1.35E-07 | NaN |  |
| 43 |  | Irritable bowel syndrome signaling pathway | 1.35E-07 | 2.357 |  |
| 44 |  | Sheddase signaling pathway | 1.70E-07 | 1.871 |  |
| 45 |  | Insulin secretion signaling pathway | 1.70E-07 | 1.214 |  |
| 46 |  | Wound healing signaling pathway | 2.40E-07 | 3.150 |  |
| 47 |  | Eicosanoid signaling | 2.40E-07 | -0.492 |  |
| 48 |  | Small cell lung cancer signaling | 2.88E-07 | 1.500 |  |
| 49 |  | Myelination signaling pathway | 2.95E-07 | 0.570 |  |
| 50 |  | Hepatic fibrosis/hepatic stellate cell activation | 3.02E-07 | NaN |  |
| 51 |  | RAR activation | 3.02E-07 | -2.530 |  |
| 52 |  | Activin inhibin signaling pathway | 3.02E-07 | 1.457 |  |
| 53 | 12 | Production of nitric oxide and reactive oxygen species in macrophages | 3.02E-07 | -0.590 |  |
| 54 |  | Glycosaminoglycan metabolism | 3.24E-07 | 0.949 |  |
| 55 |  | RAF/MAP kinase cascade | 3.31E-07 | 3.939 |  |
| 56 |  | Pulmonary fibrosis idiopathic signaling pathway | 3.72E-07 | 2.524 |  |
| 57 |  | Proteasomal PSMD10 signaling pathway | 3.72E-07 | -0.507 |  |
| 58 |  | Senescence pathway | 3.72E-07 | 0.361 |  |
| 59 |  | Role of osteoclasts in rheumatoid arthritis signaling pathway | 4.47E-07 | 2.255 |  |
| 60 |  | Tuberculosis active signaling pathway | 5.89E-07 | -2.540 |  |
| 61 |  | Interleukin-1 family signaling | 6.03E-07 | 3.162 |  |
| 62 |  | Ovarian folliculogenesis signaling pathway | 6.76E-07 | -1.291 |  |
| 63 | 13 | IL-8 signaling | 6.76E-07 | 0.302 |  |
| 64 |  | NGF-stimulated transcription | 7.08E-07 | 2.982 |  |
| 65 |  | Sertoli cell-sertoli cell junction signaling | 7.59E-07 | 0.640 |  |
| 66 |  | Hepatitis B chronic liver pathogenesis signaling pathway | 8.13E-07 | 1.155 |  |
| 67 |  | Intra-golgi and retrograde golgi-to-ER traffic | 8.13E-07 | 0.816 |  |
| 68 |  | Coronavirus pathogenesis pathway | 8.51E-07 | 2.661 |  |
| 69 |  | HIF1α signaling | 8.91E-07 | 0.272 |  |
| 70 |  | Mitotic prometaphase | 9.33E-07 | -4.355 |  |
| 71 |  | Protein ubiquitination pathway | 9.33E-07 | 3.969 |  |
| 72 | 14 | IL-17 signaling | 9.33E-07 | 3.501 |  |
| 73 |  | Kinetochore metaphase signaling pathway | 1.02E-06 | -3.528 |  |
| 74 | 15 | Immunogenic cell death signaling pathway | 1.12E-06 | 2.043 |  |
| 75 |  | Tumor microenvironment pathway | 1.17E-06 | 3.464 |  |
| 76 |  | Role of osteoblasts in rheumatoid arthritis signaling pathway | 1.23E-06 | 4.180 |  |
| 77 |  | CLEAR signaling pathway | 1.23E-06 | -1.344 |  |
| 78 |  | RHO GTPases activate formins | 1.48E-06 | -3.904 |  |
| 79 |  | Sumoylation pathway | 1.51E-06 | -0.816 |  |
| 80 |  | Superpathway of inositol phosphate compounds | 1.62E-06 | -1.043 |  |
| 81 |  | Thrombin signaling | 1.78E-06 | -0.469 |  |
| 82 |  | Epithelial membrane protein signaling pathway | 2.09E-06 | -0.885 |  |
| 83 |  | p53 signaling | 2.29E-06 | 1.732 |  |
| 84 |  | Major pathway of rRNA processing in the nucleolus and cytosol | 2.34E-06 | 7.874 |  |
| 85 |  | Epithelial adherens junction signaling | 2.34E-06 | 1.677 |  |
| 86 |  | Neutrophil degranulation | 2.88E-06 | 1.910 |  |
| 87 |  | Pulmonary healing signaling pathway | 2.88E-06 | 1.697 |  |
| 88 |  | ILK signaling | 3.09E-06 | 0.469 |  |
| 89 |  | Cell cycle checkpoints | 3.24E-06 | -1.833 |  |
| 90 |  | ID1 signaling pathway | 3.31E-06 | 1.131 |  |
| 91 |  | Gap junction signaling | 4.07E-06 | -1.213 |  |
| 92 |  | Semaphorin interactions | 4.07E-06 | 0.816 |  |
| 93 |  | Glioblastoma multiforme signaling | 4.17E-06 | 0.667 |  |
| 94 |  | 3-phosphoinositide biosynthesis | 4.37E-06 | -0.822 |  |
| 95 |  | Sirtuin signaling pathway | 4.57E-06 | -2.412 |  |
| 96 |  | NLR signaling pathways | 4.68E-06 | 0.426 |  |
| 97 |  | Unfolded protein response | 4.90E-06 | 2.524 |  |
| 98 | 16 | IL-33 signaling pathway | 5.01E-06 | 2.592 |  |
| 99 |  | Ribonucleotide reductase signaling pathway | 5.62E-06 | -0.885 |  |
| 100 |  | Hematoma resolution signaling pathway | 6.03E-06 | -2.474 |  |
| 101 |  | Folate signaling pathway | 6.46E-06 | 2.400 |  |
| 102 |  | Sertoli cell-germ cell junction signaling pathway (enhanced) | 6.61E-06 | 0.927 |  |
| 103 |  | PPAR signaling | 6.76E-06 | -0.928 |  |
| 104 |  | Role of chondrocytes in rheumatoid arthritis signaling pathway | 7.41E-06 | 2.082 |  |
| 105 | 17 | IL-12 signaling and production in macrophages | 7.76E-06 | 1.313 |  |
| 106 |  | Stress granule signaling pathway | 7.94E-06 | -1.033 |  |
| 107 |  | NOD1/2 signaling pathway | 8.13E-06 | 2.480 |  |
| 108 |  | Aldosterone signaling in epithelial cells | 9.33E-06 | -2.236 |  |
| 109 |  | Asparagine N-linked glycosylation | 9.55E-06 | 3.124 |  |
| 110 | 18 | IL-3 signaling | 1.00E-05 | -1.877 |  |
| 111 |  | HGF signaling | 1.15E-05 | -1.300 |  |
| 112 |  | Axonal guidance signaling | 1.17E-05 | NaN |  |
| 113 |  | Huntington's disease signaling | 1.17E-05 | -1.347 |  |
| 114 |  | Estrogen receptor signaling | 1.20E-05 | 0.843 |  |
| 115 |  | D-myo-inositol-5-phosphate metabolism | 1.20E-05 | -0.507 |  |
| 116 |  | UFMylation signaling pathway | 1.38E-05 | 1.043 |  |
| 117 |  | Transcriptional regulation by RUNX3 | 1.45E-05 | 2.191 |  |
| 118 |  | ERK/MAPK signaling | 1.51E-05 | -0.590 |  |
| 119 |  | 3-phosphoinositide degradation | 1.51E-05 | -0.343 |  |
| 120 |  | Serotonin receptor signaling | 1.55E-05 | 0.530 |  |
| 121 |  | Glioma signaling | 1.66E-05 | -1.528 |  |
| 122 |  | Somitogenesis | 1.70E-05 | 3.273 |  |
| 123 |  | Germ cell-sertoli cell junction signaling | 1.74E-05 | NaN |  |
| 124 | 19 | TREM1 signaling | 1.82E-05 | 1.225 |  |
| 125 |  | PPARα/RXRα activation | 1.82E-05 | -1.029 |  |
| 126 |  | Integrin to cytoskeleton signaling pathway | 2.34E-05 | -1.067 |  |
| 127 | 20 | MSP-RON signaling in macrophages pathway | 2.51E-05 | -1.512 |  |
| 128 |  | Gαq signaling | 2.51E-05 | -1.333 |  |
| 129 |  | Neddylation | 2.51E-05 | 1.192 |  |
| 130 |  | Mitotic G2-G2/M phases | 2.51E-05 | 0.566 |  |
| 131 |  | Factors promoting cardiogenesis in vertebrates | 2.63E-05 | 0.493 | X |
| 132 |  | Endocannabinoid cancer inhibition pathway | 2.75E-05 | 0.845 |  |
| 133 |  | Interleukin-10 signaling | 2.88E-05 | 2.357 |  |
| 134 |  | Unfolded protein response (UPR) | 2.88E-05 | 1.807 |  |
| 135 |  | Autophagy | 3.02E-05 | -2.219 |  |
| 136 |  | HER-2 signaling in breast cancer | 3.09E-05 | -0.849 |  |
| 137 |  | Non-small cell lung cancer signaling | 3.09E-05 | -0.832 |  |
| 138 |  | Ceramide signaling | 3.09E-05 | 0.626 |  |
| 139 |  | D-myo-inositol (1,4,5,6)-tetrakisphosphate biosynthesis | 3.09E-05 | 0.000 |  |
| 140 |  | D-myo-inositol (3,4,5,6)-tetrakisphosphate biosynthesis | 3.09E-05 | 0.000 |  |
| 141 |  | Ovarian cancer signaling | 3.24E-05 | -1.155 |  |
| 142 |  | MSP-RON signaling in cancer cells pathway | 3.31E-05 | -0.174 |  |
| 143 | 21 | IL-15 production | 3.39E-05 | 1.567 |  |
| 144 |  | Post-translational protein phosphorylation | 3.63E-05 | 4.490 | X |
| 145 |  | Mitotic G1 phase and G1/S transition | 3.63E-05 | 1.333 |  |
| 146 |  | Signaling by VEGF | 3.63E-05 | 0.898 |  |
| 147 |  | MHC class II antigen presentation | 3.80E-05 | 0.169 |  |
| 148 |  | RAC signaling | 4.07E-05 | -0.186 |  |
| 149 |  | Human embryonic stem cell pluripotency | 4.17E-05 | 1.732 |  |
| 150 |  | Preeclampsia signaling pathway | 4.17E-05 | 1.093 |  |
| 151 | 22 | IL-6 signaling | 4.47E-05 | 1.300 |  |
| 152 |  | TNFR2 non-canonical NF-kB pathway | 4.57E-05 | 4.379 |  |
| 153 |  | Mitotic roles of polo-like kinase | 4.57E-05 | -0.577 |  |
| 154 |  | Metabolism of polyamines | 4.79E-05 | 4.583 |  |
| 155 |  | PTEN signaling | 4.79E-05 | 0.539 |  |
| 156 |  | Sphingosine-1-phosphate signaling | 4.79E-05 | 0.186 |  |
| 157 |  | P2Y purinergic receptor signaling pathway | 4.90E-05 | -1.826 |  |
| 158 |  | Warburg effect signaling pathway | 5.13E-05 | 3.430 |  |
| 159 |  | PI metabolism | 5.13E-05 | -2.353 |  |
| 160 |  | PEDF signaling | 5.13E-05 | -1.091 |  |
| 161 |  | Inhibition of ARE-mediated mRNA degradation pathway | 5.50E-05 | 0.756 |  |
| 162 |  | RNA m6A methylation signaling pathway | 5.62E-05 | 0.146 |  |
| 163 |  | Pancreatic secretion signaling pathway | 6.92E-05 | -0.700 |  |
| 164 |  | Nuclear cytoskeleton signaling pathway | 7.24E-05 | -1.414 |  |
| 165 |  | TCR signaling | 7.59E-05 | 2.401 |  |
| 166 |  | S phase | 7.76E-05 | 1.300 |  |
| 167 |  | Docosahexaenoic acid (DHA) signaling | 7.76E-05 | -0.137 |  |
| 168 |  | Kinesins | 7.94E-05 | -3.273 |  |
| 169 | 23 | Leukocyte extravasation signaling | 7.94E-05 | 0.316 |  |
| 170 |  | Autism signaling pathway | 8.32E-05 | 2.066 |  |
| 171 |  | Cholecystokinin/gastrin-mediated signaling | 8.32E-05 | 0.928 |  |
| 172 |  | HEY1 signaling pathway | 8.91E-05 | 0.160 |  |
| 173 |  | Acute myeloid leukemia signaling | 9.55E-05 | -2.668 |  |
| 174 |  | Estrogen-dependent breast cancer signaling | 9.55E-05 | -0.500 |  |
| 175 |  | Neuregulin signaling | 9.77E-05 | -0.688 |  |
| 176 |  | Necroptosis signaling pathway | 1.02E-04 | 0.164 |  |
| 177 |  | PTEN regulation | 1.05E-04 | 2.920 |  |
| 178 |  | Regulation of insulin-like growth factor (IGF) transport and uptake by IGFBPs | 1.07E-04 | 4.700 | X |
| 179 |  | Mitochondrial RNA degradation | 1.07E-04 | 3.464 | X |
| 180 | 24 | Neutrophil extracellular trap signaling pathway | 1.10E-04 | 2.224 | X |
| 181 | 25 | Crosstalk between dendritic cells and natural killer cells | 1.12E-04 | 2.837 |  |
| 182 | 26 | NF-κB activation by viruses | 1.12E-04 | -0.853 |  |
| 183 |  | Semaphorin signaling in neurons | 1.15E-04 | NaN |  |
| 184 |  | Mitochondrial dysfunction | 1.20E-04 | -3.787 |  |
| 185 |  | Signaling by Rho family GTPases | 1.26E-04 | 0.447 |  |
| 186 |  | Xenobiotic metabolism signaling | 1.35E-04 | NaN |  |
| 187 |  | Histone modification signaling pathway | 1.35E-04 | -3.615 |  |
| 188 | 27 | IL-23 signaling pathway | 1.35E-04 | 1.291 |  |
| 189 |  | Adrenergic receptor signaling pathway (enhanced) | 1.45E-04 | -3.086 |  |
| 190 |  | PIP3 activates AKT signaling | 1.48E-04 | -1.480 |  |
| 191 |  | Regulation of the epithelial-mesenchymal transition pathway | 1.58E-04 | NaN |  |
| 192 |  | Regulation of the epithelial mesenchymal transition by growth factors pathway | 1.58E-04 | 1.372 |  |
| 193 |  | IL-17A signaling in fibroblasts | 1.70E-04 | 2.449 |  |
| 194 |  | Semaphorin neuronal repulsive signaling pathway | 1.70E-04 | 1.859 |  |
| 195 |  | LPS-stimulated MAPK signaling | 1.70E-04 | -0.894 |  |
| 196 | 28 | Role of PKR in interferon induction and antiviral response | 1.70E-04 | -0.707 |  |
| 197 |  | Extra-nuclear estrogen signaling | 1.70E-04 | 0.209 |  |
| 198 |  | Interleukin-2 family signaling | 1.74E-04 | 1.213 |  |
| 199 |  | DYRK1A signaling pathway | 1.74E-04 | 0.156 |  |
| 200 |  | Renin-angiotensin signaling | 1.78E-04 | -0.816 |  |
| 201 |  | Proteasome assembly | 2.00E-04 | 4.243 |  |
| 202 |  | Regulation of mitotic cell cycle | 2.00E-04 | 1.400 |  |
| 203 |  | Cyclins and cell cycle regulation | 2.00E-04 | 0.243 |  |
| 204 |  | ABC-family proteins mediated transport | 2.19E-04 | 2.335 |  |
| 205 |  | Signaling by the B cell receptor (BCR) | 2.24E-04 | 3.904 |  |
| 206 |  | Degradation of beta-catenin by the destruction complex | 2.40E-04 | 1.961 |  |
| 207 |  | Telomerase signaling | 2.51E-04 | -0.447 |  |
| 208 |  | mTOR signaling | 2.57E-04 | -0.686 |  |
| 209 |  | Glucocorticoid receptor signaling | 2.69E-04 | NaN |  |
| 210 |  | Parkinson's signaling pathway | 2.69E-04 | 1.692 |  |
| 211 |  | RANK signaling in osteoclasts | 2.82E-04 | -0.218 |  |
| 212 |  | ISGylation signaling pathway | 2.95E-04 | 0.557 |  |
| 213 |  | HOTAIR regulatory pathway | 3.09E-04 | 0.649 |  |
| 214 |  | Cereblon and thalidomide signaling pathway | 3.16E-04 | 1.265 |  |
| 215 |  | Ferroptosis signaling pathway | 3.24E-04 | 1.768 |  |
| 216 |  | FXR/RXR activation | 3.47E-04 | -3.202 |  |
| 217 | 29 | CGAS-STING signaling pathway | 3.72E-04 | 4.004 |  |
| 218 |  | Cardiac hypertrophy signaling | 3.72E-04 | -1.605 |  |
| 219 |  | Hereditary breast cancer signaling | 3.72E-04 | -0.343 |  |
| 220 | 30 | IL-7 signaling pathway | 3.72E-04 | -0.243 |  |
| 221 | 31 | Natural killer cell signaling | 3.89E-04 | 0.617 |  |
| 222 |  | Reelin signaling in neurons | 4.27E-04 | 0.392 |  |
| 223 |  | Myocardin signaling pathway | 4.37E-04 | 1.095 |  |
| 224 |  | Endoplasmic reticulum stress pathway | 4.90E-04 | 1.265 |  |
| 225 | 32 | FAT10 cancer signaling pathway | 5.01E-04 | 0.535 |  |
| 226 |  | Sphingolipid metabolism | 5.01E-04 | 0.378 |  |
| 227 | 33 | CD27 signaling in lymphocytes | 5.25E-04 | 2.840 |  |
| 228 | 34 | Antigen presentation pathway | 5.25E-04 | 1.508 | X |
| 229 |  | Interleukin-3, interleukin-5 and GM-CSF signaling | 5.25E-04 | 1.414 |  |
| 230 | 35 | FAT10 signaling pathway | 5.25E-04 | 1.000 |  |
| 231 |  | BAG2 signaling pathway | 5.25E-04 | 0.000 |  |
| 232 |  | Circadian rhythm signaling | 5.37E-04 | NaN |  |
| 233 | 36 | p38 MAPK signaling | 5.37E-04 | 1.347 |  |
| 234 |  | VEGF signaling | 5.62E-04 | -0.655 |  |
| 235 |  | Protein sorting signaling pathway | 5.75E-04 | 2.722 |  |
| 236 |  | Regulation of eIF4 and p70S6K signaling | 5.89E-04 | -1.886 |  |
| 237 |  | Beta-catenin independent WNT signaling | 5.89E-04 | 1.342 |  |
| 238 |  | Role of hypercytokinemia/hyperchemokinemia in the pathogenesis of influenza | 5.89E-04 | 1.279 |  |
| 239 |  | Cellular response to hypoxia | 6.61E-04 | 4.264 |  |
| 240 |  | Deubiquitination | 6.61E-04 | 1.838 |  |
| 241 |  | Degradation of CRY and PER proteins | 6.76E-04 | 4.359 |  |
| 242 |  | MAPK6/MAPK4 signaling | 6.92E-04 | 3.400 |  |
| 243 |  | Response to elevated platelet cytosolic Ca2+ | 7.41E-04 | 2.828 |  |
| 244 |  | JAK/STAT signaling | 7.59E-04 | 0.000 |  |
| 245 |  | FLT3 signaling | 7.94E-04 | -1.069 |  |
| 246 |  | Regulation of RUNX2 expression and activity | 8.13E-04 | 4.146 |  |
| 247 |  | COPII-mediated vesicle transport | 8.13E-04 | 2.837 | X |
| 248 |  | Growth hormone signaling | 8.13E-04 | 0.229 |  |
| 249 |  | Relaxin signaling | 8.51E-04 | -1.964 |  |
| 250 |  | TP53 regulates transcription of cell death genes | 8.91E-04 | -0.775 |  |
| 251 |  | Hedgehog 'on' state | 9.12E-04 | 1.715 |  |
| 252 |  | Bone mineralization signaling pathway | 9.12E-04 | 0.870 | X |
| 253 |  | cAMP-mediated signaling | 9.33E-04 | -2.897 |  |
| 254 |  | NAD signaling pathway | 9.33E-04 | -1.095 |  |
| 255 | 37 | iNOS signaling | 9.33E-04 | 0.577 |  |
| 256 |  | Acetylcholine receptor signaling pathway | 9.77E-04 | -1.406 |  |
| 257 |  | Cell cycle: G1/S checkpoint regulation | 9.77E-04 | -0.728 |  |
| 258 |  | GNRH signaling | 9.77E-04 | -0.343 |  |
| 259 |  | Regulation of mRNA stability by proteins that bind AU-rich elements | 1.00E-03 | 4.491 |  |
| 260 |  | C-type lectin receptors (CLRs) | 1.00E-03 | 3.087 |  |
| 261 |  | Assembly of RNA polymerase I complex | 1.00E-03 | 1.890 |  |
| 262 |  | Sleep REM signaling pathway | 1.00E-03 | 1.414 |  |
| 263 |  | Apelin cardiomyocyte signaling pathway | 1.00E-03 | -1.400 |  |
| 264 |  | Opioid signaling pathway | 1.07E-03 | 0.447 |  |
| 265 | 38 | GM-CSF signaling | 1.20E-03 | -1.213 |  |
| 266 |  | Regulation of the epithelial mesenchymal transition in development pathway | 1.23E-03 | 3.300 |  |
| 267 |  | Virus entry via endocytic pathways | 1.23E-03 | -2.200 |  |
| 268 |  | Death receptor signaling | 1.29E-03 | -0.426 |  |
| 269 |  | Fc epsilon RI signaling | 1.41E-03 | -1.789 |  |
| 270 |  | Glioma invasiveness signaling | 1.45E-03 | -0.728 |  |
| 271 |  | Hedgehog ligand biogenesis | 1.48E-03 | 4.359 | X |
| 272 |  | PDGF signaling | 1.48E-03 | -1.279 |  |
| 273 |  | UVA-induced MAPK signaling | 1.48E-03 | -1.069 |  |
| 274 |  | NIK-->noncanonical NF-kB signaling | 1.51E-03 | 4.243 |  |
| 275 |  | G-Protein coupled receptor signaling | 1.51E-03 | 1.026 |  |
| 276 |  | Adipogenesis pathway | 1.55E-03 | 1.061 |  |
| 277 | 39 | Toll-like receptor signaling | 1.62E-03 | 1.000 |  |
| 278 | 40 | CXCR4 signaling | 1.66E-03 | -0.365 |  |
| 279 | 41 | Airway inflammation in asthma | 1.78E-03 | 2.828 |  |
| 280 |  | WNT/Ca2+ signaling pathway | 1.78E-03 | 1.500 | X |
| 281 |  | Role of NFAT in cardiac hypertrophy | 1.82E-03 | -2.271 |  |
| 282 |  | TNFR1 signaling | 1.82E-03 | 1.387 |  |
| 283 | 42 | Caveolar-mediated endocytosis signaling | 1.95E-03 | -0.832 |  |
| 284 |  | TNFs bind their physiological receptors | 2.00E-03 | 2.111 |  |
| 285 |  | Response of EIF2AK1 (HRI) to heme deficiency | 2.00E-03 | 1.508 |  |
| 286 |  | Regulation of cellular mechanics by calpain protease | 2.00E-03 | -1.069 |  |
| 287 |  | Glutaminergic receptor signaling pathway (enhanced) | 2.04E-03 | 0.258 |  |
| 288 |  | Aryl hydrocarbon receptor signaling | 2.04E-03 | -0.186 |  |
| 289 |  | Apoptosis signaling | 2.09E-03 | 1.043 |  |
| 290 |  | ATF6 (ATF6-alpha) activates chaperone genes | 2.19E-03 | 2.449 |  |
| 291 |  | NRF2-mediated oxidative stress response | 2.19E-03 | 0.180 |  |
| 292 |  | Antioxidant action of vitamin C | 2.24E-03 | -1.886 |  |
| 293 | 43 | S100 family signaling pathway | 2.24E-03 | 1.878 |  |
| 294 |  | KEAP1-NFE2L2 pathway | 2.24E-03 | 1.029 |  |
| 295 |  | Neuropeptides in migraine signaling pathway | 2.24E-03 | 0.000 |  |
| 296 |  | Sperm motility | 2.29E-03 | -0.655 |  |
| 297 |  | Transcriptional regulation by RUNX1 | 2.45E-03 | 1.265 |  |
| 298 |  | Neuropathic pain signaling in dorsal horn neurons | 2.57E-03 | -0.816 |  |
| 299 |  | Adrenomedullin signaling pathway | 2.57E-03 | 0.333 |  |
| 300 |  | Synaptic long term potentiation | 2.63E-03 | -1.400 |  |
| 301 |  | Regulation of apoptosis | 2.69E-03 | 4.000 |  |
| 302 |  | Acute phase response signaling | 2.69E-03 | 2.263 |  |
| 303 |  | TP53 regulates transcription of cell cycle genes | 2.69E-03 | -0.775 |  |
| 304 |  | Cerebral malformation signaling pathway | 2.69E-03 | 0.000 |  |
| 305 |  | Signaling by NOTCH4 | 2.75E-03 | 2.475 |  |
| 306 |  | Airway pathology in chronic obstructive pulmonary disease | 2.82E-03 | NaN |  |
| 307 |  | Mouse embryonic stem cell pluripotency | 2.95E-03 | -2.132 |  |
| 308 |  | Nucleotide biosynthesis | 2.95E-03 | 1.890 | X |
| 309 |  | Estrogen-mediated S-phase entry | 2.95E-03 | 0.000 |  |
| 310 | 44 | IL-9 signaling | 2.95E-03 | 0.000 |  |
| 311 |  | Role of JAK family kinases in IL-6-type cytokine signaling | 3.02E-03 | 3.130 |  |
| 312 |  | Insertion of tail-anchored proteins into the endoplasmic reticulum membrane | 3.16E-03 | 1.667 | X |
| 313 |  | Interferon gamma signaling | 3.16E-03 | -0.408 |  |
| 314 |  | Phosphorylated BMAL1:CLOCK (ARNTL:CLOCK) activates expression of core clock genes | 3.16E-03 | -0.333 |  |
| 315 | 45 | Macropinocytosis signaling | 3.24E-03 | -1.604 |  |
| 316 |  | MYC mediated apoptosis signaling | 3.31E-03 | -2.138 |  |
| 317 |  | Signaling by TGFBR3 | 3.31E-03 | -0.258 |  |
| 318 |  | ERBB signaling | 3.47E-03 | -0.655 |  |
| 319 |  | BEX2 signaling pathway | 3.55E-03 | -0.229 |  |
| 320 |  | p75 NTR receptor-mediated signaling | 3.80E-03 | -2.058 |  |
| 321 |  | Type II diabetes mellitus signaling | 3.80E-03 | -0.447 |  |
| 322 |  | Endothelin-1 signaling | 3.80E-03 | -0.169 |  |
| 323 |  | Role of JAK1, JAK2 and TYK2 in interferon signaling | 3.98E-03 | -0.447 |  |
| 324 |  | Transcriptional activity of SMAD2/SMAD3:SMAD4 heterotrimer | 4.07E-03 | -0.775 |  |
| 325 |  | Salvage pathways of pyrimidine ribonucleotides | 4.17E-03 | 1.091 |  |
| 326 |  | Proline biosynthesis II (from arginine) | 4.27E-03 | 2.000 | X |
| 327 |  | CDK5 signaling | 4.27E-03 | -0.943 |  |
| 328 | 46 | CD40 signaling | 4.27E-03 | 0.000 |  |
| 329 |  | Hypoxia signaling in the cardiovascular system | 4.37E-03 | 2.333 |  |
| 330 |  | RAF-independent MAPK1/3 activation | 4.37E-03 | 1.667 |  |
| 331 |  | Renal cell carcinoma signaling | 4.37E-03 | -1.155 |  |
| 332 |  | rRNA processing | 4.47E-03 | 3.317 | X |
| 333 |  | Mitochondrial division signaling pathway | 4.47E-03 | -0.343 |  |
| 334 |  | Toll-like receptor cascades | 4.47E-03 | 0.302 | X |
| 335 |  | XIST epigenetic regulation signaling pathway | 4.57E-03 | 0.870 |  |
| 336 |  | Oxytocin signaling pathway | 4.68E-03 | 0.555 |  |
| 337 |  | Dermatan sulfate biosynthesis | 4.68E-03 | 0.500 | X |
| 338 |  | NFE2L2 regulating MDR associated enzymes | 4.68E-03 | 0.447 |  |
| 339 |  | Dopamine-DARPP32 feedback in cAMP signaling | 4.79E-03 | -0.928 |  |
| 340 |  | Apoptotic execution phase | 4.79E-03 | -0.775 |  |
| 341 |  | WNT/β-catenin signaling | 4.79E-03 | 0.186 |  |
| 342 |  | Prolactin signaling | 5.13E-03 | -1.886 |  |
| 343 |  | UBE2C in cell cycle progression signaling pathway | 5.13E-03 | -1.706 |  |
| 344 |  | Integrin cell surface interactions | 5.37E-03 | 0.655 |  |
| 345 |  | Thioredoxin signaling pathway | 5.37E-03 | 0.229 |  |
| 346 |  | Role of CHK proteins in cell cycle checkpoint control | 5.50E-03 | 1.155 |  |
| 347 |  | Actin cytoskeleton signaling | 5.50E-03 | -0.667 |  |
| 348 |  | Lysosome positioning signaling pathway | 5.50E-03 | -0.522 |  |
| 349 |  | Tight junction signaling | 5.62E-03 | NaN |  |
| 350 |  | MAPK targets/nuclear events mediated by MAP kinases | 5.62E-03 | 0.905 |  |
| 351 |  | Actin nucleation by ARP-WASP complex | 5.62E-03 | -0.535 |  |
| 352 |  | Complex IV assembly | 5.75E-03 | 2.673 | X |
| 353 |  | Role of BRCA1 in DNA damage response | 5.75E-03 | -2.324 |  |
| 354 |  | G alpha (12/13) signaling events | 5.75E-03 | -1.789 |  |
| 355 |  | Signaling by EGFR | 5.75E-03 | 1.291 | X |
| 356 |  | Cell cycle regulation by BTG family proteins | 5.75E-03 | -0.816 |  |
| 357 | 47 | Tumoricidal function of hepatic natural killer cells | 5.75E-03 | 0.816 |  |
| 358 |  | TGF-β signaling | 5.75E-03 | 0.471 |  |
| 359 |  | Role of JAK1 and JAK3 in γc cytokine signaling | 5.75E-03 | 0.243 |  |
| 360 |  | B cell activating factor signaling | 5.89E-03 | 2.121 |  |
| 361 |  | Platelet homeostasis | 5.89E-03 | 1.091 |  |
| 362 |  | Signaling by SCF-KIT | 5.89E-03 | 0.832 |  |
| 363 |  | Hedgehog 'off' state | 6.03E-03 | 2.353 |  |
| 364 |  | Thrombopoietin signaling | 6.03E-03 | -2.324 |  |
| 365 |  | Cytosolic sensors of pathogen-associated DNA | 6.03E-03 | 1.698 |  |
| 366 | 48 | fMLP signaling in neutrophils | 6.03E-03 | -1.569 |  |
| 367 |  | RHOGDI signaling | 6.03E-03 | 1.257 |  |
| 368 |  | Melatonin signaling | 6.03E-03 | -0.943 |  |
| 369 |  | Purine nucleotides de novo biosynthesis II | 6.17E-03 | 1.633 | X |
| 370 |  | Mitochondrial protein degradation | 6.31E-03 | 3.962 |  |
| 371 |  | DNA methylation and transcriptional repression signaling | 6.31E-03 | 0.471 |  |
| 372 |  | Transcriptional regulatory network in embryonic stem cells | 6.31E-03 | 0.343 |  |
| 373 | 49 | Granzyme B signaling | 6.46E-03 | -1.342 |  |
| 374 |  | 5-aminoimidazole ribonucleotide biosynthesis I | 6.61E-03 | NaN |  |
| 375 |  | Insulin receptor signaling | 6.61E-03 | -0.784 |  |
| 376 |  | Ephrin receptor signaling | 6.61E-03 | -0.557 |  |
| 377 |  | Cellular effects of Sildenafil (Viagra) | 6.92E-03 | -1.347 |  |
| 378 |  | Plasma lipoprotein assembly, remodeling, and clearance | 6.92E-03 | 0.229 |  |
| 379 |  | FLT3 signaling in hematopoietic progenitor cells | 7.41E-03 | -1.500 |  |
| 380 |  | Endometrial cancer signaling | 7.41E-03 | -1.387 |  |
| 381 |  | ROBO SLIT signaling pathway | 7.41E-03 | -0.962 |  |
| 382 |  | Chondroitin sulfate biosynthesis | 7.41E-03 | 0.775 | X |
| 383 |  | Ephrin A signaling | 7.41E-03 | 0.557 |  |
| 384 |  | ABRA signaling pathway | 7.59E-03 | 1.964 |  |
| 385 |  | Intrinsic pathway for apoptosis | 7.76E-03 | -1.807 |  |
| 386 |  | Lymphotoxin β receptor signaling | 7.76E-03 | -1.155 |  |
| 387 |  | Angiopoietin signaling | 7.94E-03 | -0.577 |  |
| 388 |  | ATM signaling | 7.94E-03 | -0.229 |  |
| 389 |  | PAK signaling | 8.32E-03 | -0.655 |  |
| 390 |  | Respiratory electron transport | 8.51E-03 | 4.583 | X |
| 391 |  | Microautophagy signaling pathway | 8.51E-03 | 2.353 | X |
| 392 |  | Corticotropin releasing hormone signaling | 8.51E-03 | -0.600 |  |
| 393 |  | Oncogene induced senescence | 8.51E-03 | 0.302 |  |
| 394 |  | Triacylglycerol biosynthesis | 8.71E-03 | 2.530 |  |
| 395 |  | Synaptogenesis signaling pathway | 8.71E-03 | -0.566 |  |
| 396 |  | Extracellular matrix organization | 8.91E-03 | 3.266 | X |
| 397 |  | Transport of inorganic cations/anions and amino acids/oligopeptides | 8.91E-03 | 1.633 |  |
| 398 |  | Thyroid cancer signaling | 8.91E-03 | -1.606 |  |
| 399 |  | Leptin signaling in obesity | 8.91E-03 | -1.414 |  |
| 400 |  | MicroRNA biogenesis signaling pathway | 8.91E-03 | 1.333 |  |
| 401 |  | Signaling by NOTCH1 | 8.91E-03 | 0.688 |  |
| 402 |  | Chondroitin sulfate degradation (metazoa) | 9.12E-03 | 0.378 | X |
| 403 |  | Tuberculosis latent signaling pathway | 9.77E-03 | 1.528 |  |
| 404 |  | Signaling by MET | 1.02E-02 | 2.065 |  |
| 405 |  | Role of IL-17F in allergic inflammatory airway diseases | 1.02E-02 | 1.897 |  |
| 406 |  | Nitric oxide signaling in the cardiovascular system | 1.02E-02 | -1.225 |  |
| 407 |  | tRNA processing in the mitochondrion | 1.05E-02 | 3.464 | X |
| 408 |  | Signaling by NOTCH2 | 1.05E-02 | 0.905 |  |
| 409 |  | Vitamin K signaling pathway | 1.07E-02 | -1.213 |  |
| 410 |  | NER (nucleotide excision repair, enhanced pathway) | 1.10E-02 | 1.213 |  |
| 411 |  | 14-3-3-mediated signaling | 1.10E-02 | -1.043 |  |
| 412 |  | ISG15 antiviral mechanism | 1.12E-02 | 2.357 |  |
| 413 |  | NGF signaling | 1.12E-02 | -1.279 |  |
| 414 |  | Formation of paraxial mesoderm | 1.12E-02 | -0.707 |  |
| 415 |  | VEGF family ligand-receptor interactions | 1.20E-02 | -2.065 |  |
| 416 |  | Fc epsilon receptor (FCERI) signaling | 1.20E-02 | 1.897 |  |
| 417 |  | Gα12/13 signaling | 1.20E-02 | -1.043 |  |
| 418 |  | Role of IL-17A in arthritis | 1.26E-02 | NaN |  |
| 419 |  | Mitochondrial translation | 1.26E-02 | 4.899 | X |
| 420 |  | Syndecan interactions | 1.26E-02 | 1.667 |  |
| 421 |  | IL-1 signaling | 1.26E-02 | 1.387 |  |
| 422 |  | April mediated signaling | 1.26E-02 | 1.000 |  |
| 423 |  | Turbulent (oscillatory, disturbed) flow shear stress activates signaling by PIEZO1 and integrins in endothelial cells | 1.29E-02 | 0.632 |  |
| 424 |  | Mitochondrial unfolded protein response (UPRmt) | 1.29E-02 | -0.378 |  |
| 425 |  | Dermatan sulfate degradation (Metazoa) | 1.29E-02 | -0.378 | X |
| 426 |  | Calcium transport I | 1.32E-02 | 1.342 | X |
| 427 | 50 | Granulocyte adhesion and diapedesis | 1.35E-02 | NaN | X |
| 428 |  | Synthesis of DNA | 1.35E-02 | 2.353 |  |
| 429 |  | Melanocyte development and pigmentation signaling | 1.38E-02 | -2.357 |  |
| 430 |  | DNA double-strand break repair by homologous recombination | 1.38E-02 | -2.000 |  |
| 431 |  | NF1 RAS signaling pathway | 1.38E-02 | 0.853 |  |
| 432 |  | IGF-1 signaling | 1.38E-02 | -0.688 |  |
| 433 |  | Regulation of TP53 expression and degradation | 1.55E-02 | -1.508 |  |
| 434 |  | UVB-induced MAPK signaling | 1.55E-02 | -0.832 |  |
| 435 |  | Paxillin signaling | 1.55E-02 | 0.243 |  |
| 436 |  | Breast cancer regulation by stathmin1 | 1.55E-02 | 0.105 |  |
| 437 |  | TWEAK signaling | 1.55E-02 | 0.000 |  |
| 438 |  | Signaling by TGF-beta receptor complex | 1.55E-02 | 0.000 |  |
| 439 |  | Netrin signaling | 1.58E-02 | -0.686 |  |
| 440 |  | Sialic acid metabolism | 1.58E-02 | 0.632 | X |
| 441 |  | Cytoprotection by HMOX1 | 1.66E-02 | 2.324 | X |
| 442 |  | Bladder cancer signaling | 1.66E-02 | -2.000 |  |
| 443 |  | GADD45 signaling | 1.66E-02 | -1.807 |  |
| 444 |  | Regulation of actin-based motility by Rho | 1.70E-02 | -0.243 |  |
| 445 |  | Arginine degradation VI (arginase 2 pathway) | 1.78E-02 | 2.000 | X |
| 446 | 51 | Activation of IRF by cytosolic pattern recognition receptors | 1.78E-02 | 0.775 |  |
| 447 |  | Pyridoxal 5'-phosphate salvage pathway | 1.78E-02 | 0.500 |  |
| 448 |  | CREB signaling in neurons | 1.78E-02 | -0.107 | X |
| 449 |  | Regulation of CDH19 expression and function | 1.78E-02 | 0.000 | X |
| 450 |  | Glycoaminoglycan-protein linkage region biosynthesis | 1.78E-02 | 0.000 | X |
| 451 | 52 | Role of cytokines in mediating communication between immune cells | 1.82E-02 | NaN | X |
| 452 |  | VDR/RXR activation | 1.86E-02 | 2.333 |  |
| 453 |  | Interleukin-6 family signaling | 1.86E-02 | 1.414 |  |
| 454 |  | MAP kinase activation | 1.86E-02 | 0.905 |  |
| 455 |  | Other interleukin signaling | 1.86E-02 | 0.000 |  |
| 456 |  | RHO GTPases activate NADPH oxidases | 1.86E-02 | 0.000 | X |
| 457 |  | WNT/SHH axonal guidance signaling pathway | 1.91E-02 | 2.502 |  |
| 458 |  | Mitotic telophase/cytokinesis | 1.91E-02 | -1.633 |  |
| 459 | 53 | 4-1BB signaling in T lymphocytes | 1.91E-02 | 1.633 |  |
| 460 |  | Myogenesis | 1.91E-02 | -1.000 | X |
| 461 |  | AMPK signaling | 1.91E-02 | -0.378 |  |
| 462 |  | SUMOylation of intracellular receptors | 1.91E-02 | -0.333 | X |
| 463 |  | Proline biosynthesis I | 1.95E-02 | NaN |  |
| 464 |  | Signaling by NOTCH3 | 1.95E-02 | 1.387 |  |
| 465 |  | Cilium assembly | 1.95E-02 | -1.298 |  |
| 466 |  | Ribavirin ADME | 1.95E-02 | -0.500 |  |
| 467 |  | Phosphorylation of CLOCK, acetylation of BMAL1 (ARNTL) at target gene promoters | 2.00E-02 | 1.342 | X |
| 468 |  | Glycogen degradation II | 2.00E-02 | -0.447 |  |
| 469 |  | NFE2L2 regulating tumorigenic genes | 2.00E-02 | 0.447 |  |
| 470 |  | TP53 regulates metabolic genes | 2.09E-02 | 0.229 |  |
| 471 | 54 | MSP-RON signaling pathway | 2.19E-02 | NaN |  |
| 472 |  | UVC-induced MAPK signaling | 2.24E-02 | -1.508 |  |
| 473 |  | IL-17A signaling in airway cells | 2.24E-02 | -1.069 |  |
| 474 |  | Purine nucleotides degradation II (aerobic) | 2.24E-02 | -0.447 | X |
| 475 |  | Transcriptional regulation by NPAS4 | 2.29E-02 | 1.897 |  |
| 476 |  | Clathrin-mediated endocytosis | 2.29E-02 | 1.177 |  |
| 477 |  | ERK5 signaling | 2.29E-02 | 1.069 |  |
| 478 |  | eNOS signaling | 2.34E-02 | -3.273 |  |
| 479 |  | L1CAM interactions | 2.34E-02 | -0.200 |  |
| 480 | 55 | MIF regulation of innate immunity | 2.40E-02 | 2.111 |  |
| 481 |  | Transcriptional regulation by MECP2 | 2.45E-02 | 0.775 | X |
| 482 |  | Degradation of the extracellular matrix | 2.57E-02 | 2.828 | X |
| 483 |  | Rap1 signaling | 2.57E-02 | -2.449 |  |
| 484 |  | DHCR24 signaling pathway | 2.57E-02 | -1.134 |  |
| 485 |  | Signaling by NTRK1 (TRKA) | 2.57E-02 | 0.471 |  |
| 486 |  | Cancer drug resistance by drug efflux | 2.75E-02 | -0.632 |  |
| 487 | 56 | Phagosome formation | 2.75E-02 | 0.000 | X |
| 488 |  | Sphingomyelin metabolism | 2.88E-02 | NaN |  |
| 489 |  | Hematopoiesis from multipotent stem cells | 2.88E-02 | NaN | X |
| 490 |  | UDP-N-acetyl-D-galactosamine biosynthesis II | 2.88E-02 | 2.236 | X |
| 491 |  | Z-decay: degradation of maternal mRNAs by zygotically expressed factors | 2.88E-02 | 1.342 |  |
| 492 |  | γ-glutamyl cycle | 2.88E-02 | 0.000 | X |
| 493 |  | Cilia biogenesis signaling pathway | 3.09E-02 | -1.333 |  |
| 494 |  | Apelin endothelial signaling pathway | 3.09E-02 | -0.853 |  |
| 495 |  | Mitochondrial protein import | 3.24E-02 | 3.357 |  |
| 496 |  | Interferon alpha/beta signaling | 3.24E-02 | -0.728 |  |
| 497 |  | DNA damage bypass | 3.31E-02 | 1.155 |  |
| 498 |  | Metabolism of folate and pterines | 3.47E-02 | 2.449 |  |
| 499 |  | Pyroptosis signaling pathway | 3.47E-02 | 1.414 |  |
| 500 |  | Adenosine nucleotides degradation II | 3.47E-02 | -1.000 | X |
| 501 |  | Mechanical load activates signaling by PIEZO1 and integrins in osteocytes | 3.47E-02 | 0.816 | X |
| 502 |  | BMP signaling pathway | 3.55E-02 | -0.832 | X |
| 503 |  | Nucleotide excision repair | 3.63E-02 | 1.706 |  |
| 504 |  | Dopamine receptor signaling | 3.63E-02 | -1.633 |  |
| 505 |  | Neurotrophin/TRK signaling | 3.63E-02 | -1.604 |  |
| 506 |  | Integrin signaling | 3.63E-02 | -1.414 |  |
| 507 |  | EIF2 signaling | 3.63E-02 | -0.784 |  |
| 508 |  | Endocannabinoid developing neuron pathway | 3.63E-02 | -0.688 |  |
| 509 |  | Oncostatin M signaling | 3.63E-02 | 0.000 | X |
| 510 |  | Induction of apoptosis by HIV1 | 3.63E-02 | 0.000 |  |
| 511 |  | Mitotic prophase | 3.72E-02 | -1.347 |  |
| 512 |  | S-methyl-5'-thioadenosine degradation II | 3.89E-02 | NaN | X |
| 513 |  | Putrescine biosynthesis III | 3.89E-02 | NaN | X |
| 514 | 57 | MIF-mediated glucocorticoid regulation | 3.89E-02 | 2.333 |  |
| 515 |  | S-adenosylmethionine regulatory signaling pathway | 3.89E-02 | 1.886 |  |
| 516 |  | Notch signaling | 3.89E-02 | 1.633 | X |
| 517 |  | Tetrahydrofolate salvage from 5,10-methenyltetrahydrofolate | 3.98E-02 | NaN | X |
| 518 |  | Folate polyglutamylation | 3.98E-02 | NaN | X |
| 519 |  | SPINK1 general cancer pathway | 3.98E-02 | -1.941 |  |
| 520 |  | Role of PI3K/AKT signaling in the pathogenesis of influenza | 3.98E-02 | -1.155 |  |
| 521 |  | ESR-mediated signaling | 3.98E-02 | 0.928 | X |
| 522 |  | Glycogen degradation III | 3.98E-02 | -0.447 |  |
| 523 |  | Oxidative phosphorylation | 4.07E-02 | 4.472 | X |
| 524 |  | TNFR2 signaling | 4.07E-02 | 2.449 |  |
| 525 |  | Role of NANOG in mammalian embryonic stem cell pluripotency | 4.07E-02 | -2.309 | X |
| 526 |  | TBC/RABGAPs | 4.07E-02 | -1.508 |  |
| 527 |  | Inhibition of angiogenesis by TSP1 | 4.07E-02 | -1.134 |  |
| 528 |  | TAK1-dependent IKK and NF-kappa-B activation | 4.07E-02 | 0.905 |  |
| 529 |  | LXR/RXR activation | 4.17E-02 | -1.279 | X |
| 530 |  | Amyotrophic lateral sclerosis signaling | 4.17E-02 | -0.943 |  |
| 531 |  | Meiosis initiation signaling pathway | 4.17E-02 | -0.894 |  |
| 532 |  | Differential regulation of cytokine production in intestinal epithelial cells by IL-17A and IL-17F | 4.37E-02 | 2.646 |  |
| 533 |  | Cardiac β-adrenergic signaling | 4.37E-02 | -2.558 |  |
| 534 |  | Differential regulation of cytokine production in macrophages and T helper cells by IL-17A and IL-17F | 4.37E-02 | 2.449 |  |
| 535 |  | Interleukin-1 processing | 4.37E-02 | 2.000 | X |
| 536 |  | Nucleotide salvage | 4.37E-02 | 1.890 | X |
| 537 | 58 | Granzyme A signaling | 4.37E-02 | -1.604 | X |
| 538 |  | Cellular response to mitochondrial stress | 4.37E-02 | 1.134 | X |
| 539 |  | FGF signaling | 4.37E-02 | -1.000 |  |
| 540 |  | Deadenylation-dependent mRNA decay | 4.37E-02 | 0.832 |  |
| 541 |  | EGF signaling | 4.37E-02 | -0.577 |  |
| 542 |  | CSDE1 signaling pathway | 4.37E-02 | 0.577 | X |
| 543 |  | Telomere maintenance | 4.37E-02 | 0.000 |  |
| 544 |  | Xenobiotic metabolism general signaling pathway | 4.57E-02 | -1.671 |  |
| 545 |  | Cargo concentration in the ER | 4.79E-02 | 3.000 | X |
| 546 |  | NR1H2 and NR1H3-mediated signaling | 4.79E-02 | -1.155 | X |
| 547 |  | Transcriptional regulation by RUNX2 | 4.79E-02 | 0.577 |  |
| 548 | 59 | Primary immunodeficiency signaling | 4.90E-02 | NaN | X |
| 549 | 60 | IL-2 signaling | 4.90E-02 | -1.732 |  |
| 550 |  | Trans-golgi network vesicle budding | 4.90E-02 | 1.000 |  |
| 551 |  | NCAM signaling for neurite out-growth | 4.90E-02 | 0.000 | X |

**Supplementary Table 3:** Ingenuity Pathway Analysis® (IPA®) for down- and up-regulated differentially-expressed genes (DEGs) of Tc2 cells compared to Tc1 (fold change > |1.5|, P_adjusted_ < 0.05). Ranking of canonical and cellular immune response pathways are displayed based on their overall ranking with a Benjamini-Hochberg (BH) P < 0.05.

| **Ranking # canonical pathways** | **Ranking**  **# immune pathways** | **Ingenuity canonical pathways** | **BH**  **p-value** | **Z-score** |
| --- | --- | --- | --- | --- |
| 1 |  | Multiple sclerosis signaling pathway | 1.58E-22 | -0.372 |
| 2 |  | Pathogen induced cytokine storm signaling pathway | 5.01E-20 | -0.333 |
| 3 | 1 | Macrophage classical activation signaling pathway | 2.51E-19 | -0.832 |
| 4 | 2 | Th1 and Th2 activation pathway | 1.00E-18 | NaN |
| 5 |  | Molecular mechanisms of cancer | 1.58E-18 | 1.589 |
| 6 |  | Cardiac hypertrophy signaling (enhanced) | 2.00E-18 | 0.108 |
| 7 |  | Glycation signaling pathway | 3.16E-18 | -2.394 |
| 8 |  | CDX gastrointestinal cancer signaling pathway | 3.16E-17 | 1.905 |
| 9 |  | IL-27 signaling pathway | 3.16E-17 | -0.447 |
| 10 |  | Tuberculosis active signaling pathway | 6.31E-17 | 1.500 |
| 11 | 3 | Interleukin-4 and interleukin-13 signaling | 1.58E-16 | 0.781 |
| 12 |  | Erythropoietin signaling pathway | 2.51E-16 | 0.714 |
| 13 | 4 | Th2 pathway | 3.16E-16 | 2.137 |
| 14 | 5 | Role of pattern recognition receptors in recognition of bacteria and viruses | 5.01E-16 | -0.853 |
| 15 |  | Role of osteoblasts in rheumatoid arthritis signaling pathway | 1.00E-15 | -0.651 |
| 16 |  | Wound healing signaling pathway | 1.58E-15 | -1.408 |
| 17 | 6 | HMGB1 signaling | 2.00E-15 | -1.061 |
| 18 |  | Tumor microenvironment pathway | 3.16E-15 | -0.429 |
| 20 |  | Neutrophil degranulation | 7.94E-15 | 3.250 |
| 19 |  | NAFLD signaling pathway | 7.94E-15 | -1.069 |
| 21 |  | Role of macrophages, fibroblasts and endothelial cells in rheumatoid arthritis | 1.00E-14 | -2.390 |
| 22 |  | ID1 signaling pathway | 3.16E-14 | 1.387 |
| 23 |  | Hematoma resolution signaling pathway | 3.16E-14 | 1.033 |
| 24 |  | Regulation of the epithelial mesenchymal transition by growth factors pathway | 6.31E-14 | -2.309 |
| 25 |  | Pulmonary hypertension signaling pathway | 7.94E-14 | 0.000 |
| 26 |  | Pulmonary fibrosis idiopathic signaling pathway | 1.26E-13 | -1.083 |
| 27 |  | Chronic myeloid leukemia signaling | 1.58E-13 | 0.775 |
| 28 |  | Gap junction signaling | 2.00E-13 | -1.250 |
| 29 | 7 | Th1 pathway | 2.51E-13 | 0.174 |
| 30 |  | Hepatic fibrosis signaling pathway | 6.31E-13 | -1.414 |
| 32 |  | RAR activation | 7.94E-13 | 1.359 |
| 31 | 8 | Macrophage alternative activation signaling pathway | 7.94E-13 | 1.043 |
| 34 |  | Role of tissue factor in cancer | 1.26E-12 | -1.000 |
| 33 |  | Senescence pathway | 1.26E-12 | 0.258 |
| 35 |  | Hepatic cholestasis | 2.00E-12 | -1.664 |
| 36 |  | Autism signaling pathway | 2.00E-12 | -0.640 |
| 37 | 9 | IL-10 signaling | 5.01E-12 | 1.947 |
| 38 |  | Eicosanoid signaling | 5.01E-12 | 0.267 |
| 39 |  | Tuberculosis latent signaling pathway | 6.31E-12 | 1.616 |
| 40 |  | Sheddase signaling pathway | 6.31E-12 | 0.429 |
| 41 | 10 | CGAS-STING signaling pathway | 7.94E-12 | -1.622 |
| 42 |  | Colorectal cancer metastasis signaling | 7.94E-12 | 0.295 |
| 43 |  | Pulmonary healing signaling pathway | 1.58E-11 | -0.885 |
| 44 | 11 | IL-17 signaling | 2.00E-11 | -2.064 |
| 46 |  | Hepatic fibrosis/hepatic stellate cell activation | 3.16E-11 | NaN |
| 45 | 12 | PD-1, PD-L1 cancer immunotherapy pathway | 3.16E-11 | -1.732 |
| 48 |  | Role of chondrocytes in rheumatoid arthritis signaling pathway | 1.23E-10 | -1.808 |
| 47 |  | Irritable bowel syndrome signaling pathway | 1.23E-10 | 0.000 |
| 49 |  | Parkinson's signaling pathway | 1.45E-10 | -1.722 |
| 50 |  | Cachexia signaling pathway | 1.86E-10 | -1.861 |
| 51 |  | CGRP in inflammation and nociception signaling pathway | 2.29E-10 | 1.480 |
| 52 | 13 | Crosstalk between dendritic cells and natural killer cells | 3.39E-10 | -1.342 |
| 53 |  | Neuroinflammation signaling pathway | 3.47E-10 | 0.849 |
| 54 |  | Role of osteoblasts, osteoclasts and chondrocytes in rheumatoid arthritis | 4.47E-10 | NaN |
| 55 |  | JAK/STAT signaling | 6.92E-10 | 0.000 |
| 56 |  | Myelination signaling pathway | 9.77E-10 | 0.397 |
| 57 | 14 | IL-12 signaling and production in macrophages | 1.17E-09 | -4.143 |
| 58 |  | Adrenergic receptor signaling pathway (enhanced) | 1.41E-09 | 1.852 |
| 59 |  | Response to elevated platelet cytosolic Ca2+ | 2.69E-09 | 0.686 |
| 60 |  | Adrenomedullin signaling pathway | 2.69E-09 | 0.493 |
| 61 |  | Ovarian folliculogenesis signaling pathway | 3.47E-09 | 1.443 |
| 62 |  | Endocannabinoid cancer inhibition pathway | 3.47E-09 | -0.522 |
| 63 |  | Protein kinase A signaling | 3.63E-09 | 0.566 |
| 64 |  | CLEAR signaling pathway | 4.79E-09 | 1.633 |
| 65 |  | Osteoarthritis pathway | 5.13E-09 | -0.316 |
| 66 |  | Human embryonic stem cell pluripotency | 5.25E-09 | -1.067 |
| 67 | 15 | IL-6 signaling | 5.37E-09 | -2.414 |
| 68 | 16 | IL-7 signaling pathway | 5.75E-09 | 0.218 |
| 69 |  | Sertoli cell-sertoli cell junction signaling | 5.75E-09 | 0.146 |
| 70 |  | FXR/RXR activation | 7.41E-09 | 1.761 |
| 71 |  | Epithelial membrane protein signaling pathway | 7.59E-09 | 0.324 |
| 73 |  | HER-2 signaling in breast cancer | 7.76E-09 | -2.236 |
| 72 |  | Hepatitis B chronic liver pathogenesis signaling pathway | 7.76E-09 | 0.469 |
| 74 |  | Role of osteoclasts in rheumatoid arthritis signaling pathway | 9.33E-09 | 0.140 |
| 75 |  | Glioma signaling | 1.66E-08 | -0.655 |
| 76 | 17 | S100 family signaling pathway | 1.91E-08 | 1.675 |
| 78 |  | Glioblastoma multiforme signaling | 2.09E-08 | 0.539 |
| 77 |  | Interleukin-10 signaling | 2.09E-08 | 0.000 |
| 79 |  | Axonal guidance signaling | 2.51E-08 | NaN |
| 80 |  | Cell cycle control of chromosomal replication | 2.57E-08 | 3.638 |
| 81 |  | Ceramide signaling | 2.63E-08 | -0.426 |
| 82 |  | Opioid signaling pathway | 3.02E-08 | 0.590 |
| 84 |  | Acute myeloid leukemia signaling | 3.24E-08 | -1.606 |
| 83 |  | Activin inhibin signaling pathway | 3.24E-08 | 0.152 |
| 85 |  | HEY1 signaling pathway | 3.72E-08 | 1.000 |
| 86 |  | Signaling by VEGF | 5.25E-08 | -1.134 |
| 87 |  | Renin-angiotensin signaling | 7.24E-08 | -0.816 |
| 88 |  | RNA m6A methylation signaling pathway | 9.33E-08 | -0.632 |
| 89 |  | Type II diabetes mellitus signaling | 9.55E-08 | -1.789 |
| 90 |  | PEDF signaling | 9.77E-08 | 0.000 |
| 91 |  | Breast cancer regulation by stathmin1 | 1.10E-07 | 1.222 |
| 93 |  | STAT3 pathway | 1.20E-07 | 0.688 |
| 92 |  | PI3K/AKT signaling | 1.20E-07 | -0.626 |
| 94 |  | Pancreatic adenocarcinoma signaling | 1.23E-07 | -1.279 |
| 95 |  | p53 signaling | 1.26E-07 | 1.091 |
| 96 | 18 | CD40 signaling | 1.29E-07 | -0.728 |
| 98 |  | RHO GTPase cycle | 1.48E-07 | 2.047 |
| 97 |  | NOD1/2 signaling pathway | 1.48E-07 | -1.298 |
| 99 |  | Regulation of the epithelial-mesenchymal transition pathway | 1.70E-07 | NaN |
| 100 |  | Glycosaminoglycan metabolism | 1.70E-07 | 1.826 |
| 101 |  | Fc epsilon RI signaling | 1.70E-07 | -0.426 |
| 102 | 19 | IL-23 signaling pathway | 1.86E-07 | -2.138 |
| 103 | 20 | IL-33 signaling pathway | 1.91E-07 | -0.822 |
| 104 |  | G-protein coupled receptor signaling | 1.95E-07 | 1.141 |
| 105 |  | Docosahexaenoic acid (DHA) signaling | 2.00E-07 | -0.149 |
| 106 |  | MSP-RON signaling in cancer cells pathway | 2.19E-07 | 0.000 |
| 107 |  | Ribonucleotide reductase signaling pathway | 2.57E-07 | 1.372 |
| 108 |  | NAP1L1 transcription regulation signaling pathway | 2.82E-07 | 0.426 |
| 109 |  | Hereditary breast cancer signaling | 3.16E-07 | -0.365 |
| 110 | 21 | IL-8 signaling | 3.63E-07 | 0.000 |
| 111 |  | PI metabolism | 3.98E-07 | 0.209 |
| 112 | 22 | NF-κB activation by viruses | 4.27E-07 | -0.447 |
| 113 |  | Serotonin receptor signaling | 4.37E-07 | 0.985 |
| 114 |  | Thrombin signaling | 4.47E-07 | 0.522 |
| 117 |  | Small cell lung cancer signaling | 4.57E-07 | -1.291 |
| 116 | 23 | fMLP signaling in neutrophils | 4.57E-07 | 0.600 |
| 115 |  | 14-3-3-mediated signaling | 4.57E-07 | -0.218 |
| 118 |  | PIP3 activates AKT signaling | 4.68E-07 | -0.707 |
| 119 |  | Sphingosine-1-phosphate signaling | 4.68E-07 | -0.392 |
| 120 |  | Glucocorticoid receptor signaling | 4.79E-07 | NaN |
| 121 |  | Ephrin A signaling | 4.90E-07 | 0.730 |
| 122 | 24 | TREM1 signaling | 5.01E-07 | -0.229 |
| 123 |  | P2Y purinergic receptor signaling pathway | 6.17E-07 | -0.200 |
| 124 | 25 | Granulocyte adhesion and diapedesis | 6.46E-07 | NaN |
| 125 |  | Airway pathology in chronic obstructive pulmonary disease | 6.76E-07 | NaN |
| 126 | 26 | Cyclophilin signaling pathway | 7.08E-07 | 0.000 |
| 127 |  | Ovarian cancer signaling | 7.59E-07 | 0.243 |
| 128 | 27 | IL-3 signaling | 7.94E-07 | -0.894 |
| 130 |  | Acute phase response signaling | 8.32E-07 | -0.522 |
| 129 |  | Acetylcholine receptor signaling pathway | 8.32E-07 | 0.000 |
| 131 |  | Warburg effect signaling pathway | 8.71E-07 | 0.577 |
| 132 |  | Proteasomal PSMD10 signaling pathway | 9.12E-07 | 0.378 |
| 133 |  | Prostate cancer signaling | 9.33E-07 | 0.000 |
| 134 | 28 | p38 MAPK signaling | 1.07E-06 | -0.816 |
| 135 |  | Apoptotic execution phase | 1.15E-06 | 1.213 |
| 136 |  | HIF1α signaling | 1.15E-06 | 0.801 |
| 137 | 29 | Natural killer cell signaling | 1.17E-06 | -1.029 |
| 138 | 30 | Phagosome formation | 1.55E-06 | 2.466 |
| 139 |  | RAF/MAP kinase cascade | 1.55E-06 | -0.149 |
| 141 |  | ILK signaling | 1.74E-06 | 0.378 |
| 140 |  | HOTAIR regulatory pathway | 1.74E-06 | 0.180 |
| 142 |  | Apoptosis signaling | 1.95E-06 | -0.209 |
| 143 |  | Aryl hydrocarbon receptor signaling | 2.00E-06 | 0.209 |
| 144 | 31 | Neutrophil extracellular trap signaling pathway | 2.09E-06 | 1.313 |
| 145 |  | PTEN signaling | 2.09E-06 | -0.600 |
| 146 |  | Growth hormone signaling | 2.29E-06 | 0.229 |
| 147 |  | CREB signaling in neurons | 2.63E-06 | 2.458 |
| 149 |  | PPAR signaling | 2.69E-06 | 2.132 |
| 148 |  | Apelin endothelial signaling pathway | 2.69E-06 | -0.626 |
| 150 |  | Thioredoxin signaling pathway | 2.82E-06 | -1.342 |
| 151 |  | NF1 RAS signaling pathway | 2.88E-06 | 1.162 |
| 152 |  | HGF signaling | 3.16E-06 | 0.626 |
| 153 |  | Sertoli cell-germ cell junction signaling pathway (enhanced) | 3.24E-06 | -0.781 |
| 154 |  | Interleukin-3, interleukin-5 and GM-CSF signaling | 3.39E-06 | -0.243 |
| 155 |  | Myocardin signaling pathway | 3.63E-06 | 0.392 |
| 157 |  | TNFs bind their physiological receptors | 3.80E-06 | -2.309 |
| 156 |  | Interconversion of nucleotide di- and triphosphates | 3.80E-06 | 2.309 |
| 158 |  | Prolactin signaling | 3.89E-06 | -0.447 |
| 159 |  | Activation of the pre-replicative complex | 5.62E-06 | 3.606 |
| 162 | 32 | MSP-RON signaling in macrophages pathway | 5.75E-06 | 1.342 |
| 160 |  | UVA-induced MAPK signaling | 5.75E-06 | 1.213 |
| 161 |  | Estrogen receptor signaling | 5.75E-06 | 0.832 |
| 163 |  | Glioma invasiveness signaling | 6.03E-06 | 0.943 |
| 164 |  | GP6 signaling pathway | 6.17E-06 | 0.200 |
| 165 | 33 | Production of nitric oxide and reactive oxygen species in macrophages | 6.46E-06 | -1.671 |
| 166 |  | Gαq signaling | 6.92E-06 | 0.392 |
| 167 |  | Adipogenesis pathway | 8.71E-06 | 0.378 |
| 168 |  | Autophagy | 8.91E-06 | 0.649 |
| 169 |  | Endothelin-1 signaling | 9.33E-06 | 2.121 |
| 170 |  | Apelin cardiomyocyte signaling pathway | 9.55E-06 | 0.426 |
| 171 |  | TR/RXR activation | 9.55E-06 | 0.392 |
| 172 |  | Pyroptosis signaling pathway | 1.12E-05 | -0.447 |
| 173 |  | Signaling by NTRK1 (TRKA) | 1.17E-05 | 0.000 |
| 174 |  | Lymphotoxin β receptor signaling | 1.23E-05 | -1.604 |
| 177 |  | MYC mediated apoptosis signaling | 1.66E-05 | 0.258 |
| 175 |  | Death receptor signaling | 1.66E-05 | -0.229 |
| 176 |  | Melanoma signaling | 1.66E-05 | 0.000 |
| 178 |  | MHC class II antigen presentation | 1.91E-05 | 3.530 |
| 179 |  | Angiopoietin signaling | 2.09E-05 | 0.577 |
| 180 | 34 | Leukocyte extravasation signaling | 2.14E-05 | 0.928 |
| 181 |  | Preeclampsia signaling pathway | 2.19E-05 | 0.000 |
| 182 |  | Cellular effects of Sildenafil (Viagra) | 2.24E-05 | -0.663 |
| 183 |  | Mismatch repair in eukaryotes | 2.34E-05 | 2.333 |
| 186 |  | Asparagine N-linked glycosylation | 2.45E-05 | 3.530 |
| 184 |  | Role of NFAT in cardiac hypertrophy | 2.45E-05 | 0.898 |
| 185 |  | LPS-stimulated MAPK signaling | 2.45E-05 | -0.500 |
| 187 |  | Xenobiotic metabolism signaling | 2.69E-05 | NaN |
| 188 |  | Role of NANOG in mammalian embryonic stem cell pluripotency | 2.75E-05 | 0.000 |
| 189 |  | Sleep REM signaling pathway | 3.16E-05 | 0.000 |
| 190 | 35 | IL-9 signaling | 3.31E-05 | -0.707 |
| 191 |  | Induction of apoptosis by HIV1 | 3.39E-05 | -0.775 |
| 193 |  | PPARα/RXRα activation | 3.55E-05 | 1.706 |
| 194 |  | IL-17A signaling in fibroblasts | 3.55E-05 | -1.606 |
| 192 |  | Cholecystokinin/gastrin-mediated signaling | 3.55E-05 | 0.626 |
| 195 |  | Interleukin-2 family signaling | 3.55E-05 | 0.000 |
| 197 |  | Mitochondrial division signaling pathway | 3.80E-05 | 1.095 |
| 196 |  | GADD45 signaling | 3.80E-05 | 0.000 |
| 198 |  | Antioxidant action of vitamin C | 3.98E-05 | 0.277 |
| 200 |  | Circadian rhythm signaling | 4.17E-05 | NaN |
| 199 |  | Mouse embryonic stem cell pluripotency | 4.17E-05 | 0.447 |
| 202 |  | GNRH signaling | 4.47E-05 | 1.732 |
| 201 |  | Non-small cell lung cancer signaling | 4.47E-05 | 0.500 |
| 203 |  | NAD signaling pathway | 4.47E-05 | 0.000 |
| 204 |  | DHCR24 signaling pathway | 4.57E-05 | 0.962 |
| 205 | 36 | CXCR4 signaling | 4.68E-05 | 1.000 |
| 206 |  | FLT3 signaling in hematopoietic progenitor cells | 4.79E-05 | -0.243 |
| 207 |  | Estrogen-dependent breast cancer signaling | 4.79E-05 | 0.000 |
| 208 |  | IL-17A signaling in airway cells | 4.79E-05 | 0.000 |
| 209 |  | Melatonin signaling | 4.90E-05 | -0.258 |
| 210 |  | 3-phosphoinositide degradation | 5.01E-05 | -1.347 |
| 211 |  | IGF-1 signaling | 5.50E-05 | 0.447 |
| 212 |  | Insulin secretion signaling pathway | 5.62E-05 | 1.333 |
| 214 | 37 | Macropinocytosis signaling | 5.75E-05 | -0.775 |
| 213 |  | NGF signaling | 5.75E-05 | 0.655 |
| 215 |  | Cerebral malformation signaling pathway | 5.75E-05 | 0.000 |
| 218 |  | LXR/RXR activation | 6.61E-05 | 2.236 |
| 219 | 38 | Systemic lupus erythematosus in B cell signaling pathway | 6.61E-05 | -1.342 |
| 217 | 39 | Role of PKR in interferon induction and antiviral response | 6.61E-05 | -0.853 |
| 216 |  | Oxytocin signaling pathway | 6.61E-05 | 0.617 |
| 220 |  | Sphingolipid metabolism | 7.24E-05 | 1.706 |
| 221 |  | Renal cell carcinoma signaling | 8.32E-05 | 0.277 |
| 222 |  | Cholesterol biosynthesis I | 8.51E-05 | 2.646 |
| 223 |  | Cholesterol biosynthesis II (via 24,25-dihydrolanosterol) | 8.51E-05 | 2.646 |
| 224 |  | Cholesterol biosynthesis III (via desmosterol) | 8.51E-05 | 2.646 |
| 225 | 40 | Fcγ receptor-mediated phagocytosis in macrophages and monocytes | 8.51E-05 | 0.000 |
| 226 | 41 | FAT10 cancer signaling pathway | 8.71E-05 | -2.111 |
| 227 |  | Synaptic long term potentiation | 9.77E-05 | 1.964 |
| 229 |  | ERK/MAPK signaling | 1.07E-04 | 1.257 |
| 228 |  | Ephrin receptor signaling | 1.07E-04 | 1.043 |
| 230 |  | Neuregulin signaling | 1.10E-04 | -0.243 |
| 231 |  | Integrin to cytoskeleton signaling pathway | 1.17E-04 | 1.095 |
| 232 |  | Role of IL-17F in allergic inflammatory airway diseases | 1.20E-04 | 0.000 |
| 233 |  | Sperm motility | 1.23E-04 | 1.147 |
| 234 |  | Virus entry via endocytic pathways | 1.26E-04 | 0.218 |
| 235 |  | Lung Ionic balance signaling pathway | 1.29E-04 | 2.250 |
| 236 |  | ERK5 signaling | 1.41E-04 | 0.728 |
| 237 |  | Bone mineralization signaling pathway | 1.48E-04 | -1.000 |
| 238 |  | Superpathway of inositol phosphate compounds | 1.51E-04 | -0.898 |
| 239 |  | Cereblon and thalidomide signaling pathway | 1.55E-04 | -0.928 |
| 241 |  | 3-phosphoinositide biosynthesis | 1.66E-04 | -1.400 |
| 240 |  | UVB-induced MAPK signaling | 1.66E-04 | 0.277 |
| 243 |  | DYRK1A signaling pathway | 1.70E-04 | -0.186 |
| 242 |  | BMP signaling pathway | 1.70E-04 | 0.000 |
| 244 | 42 | Tumoricidal function of hepatic natural killer cells | 1.78E-04 | 1.134 |
| 245 |  | RAC signaling | 1.91E-04 | 0.943 |
| 246 |  | Relaxin signaling | 1.95E-04 | -0.258 |
| 248 |  | Synthesis of DNA | 2.04E-04 | 3.962 |
| 247 |  | Role of JAK1 and JAK3 in γc cytokine signaling | 2.04E-04 | 0.258 |
| 249 |  | Superpathway of cholesterol biosynthesis | 2.09E-04 | 3.000 |
| 250 |  | Oxytocin in brain signaling pathway | 2.19E-04 | 1.976 |
| 251 |  | Mitochondrial dysfunction | 2.19E-04 | -1.508 |
| 252 |  | Telomerase signaling | 2.34E-04 | 0.000 |
| 253 | 43 | GM-CSF signaling | 2.40E-04 | -1.069 |
| 254 |  | CNTF signaling | 2.45E-04 | 0.000 |
| 257 |  | B Cell activating factor signaling | 2.57E-04 | -1.414 |
| 255 |  | Sirtuin signaling pathway | 2.57E-04 | -1.257 |
| 256 |  | ERBB signaling | 2.57E-04 | -0.471 |
| 258 |  | Thyroid cancer signaling | 2.63E-04 | 0.728 |
| 259 |  | Gα12/13 signaling | 2.75E-04 | 1.091 |
| 260 |  | Regulation of the epithelial mesenchymal transition in development pathway | 2.82E-04 | 0.535 |
| 261 |  | Synaptogenesis signaling pathway | 2.88E-04 | 0.973 |
| 262 |  | Neuropathic pain signaling in dorsal horn neurons | 3.02E-04 | 1.147 |
| 263 |  | BER (base excision repair) pathway | 3.24E-04 | 1.732 |
| 264 |  | PDGF signaling | 3.24E-04 | 0.000 |
| 265 | 44 | Immunogenic cell death signaling pathway | 3.39E-04 | 0.000 |
| 269 |  | Role of IL-17A in arthritis | 3.47E-04 | NaN |
| 267 |  | Signaling by PDGF | 3.47E-04 | -1.069 |
| 268 |  | Role of CHK proteins in cell cycle checkpoint control | 3.47E-04 | 0.707 |
| 266 |  | Transcriptional regulation by RUNX2 | 3.47E-04 | -0.277 |
| 270 |  | D-myo-inositol-5-phosphate metabolism | 3.55E-04 | -0.816 |
| 272 |  | XIST epigenetic regulation signaling pathway | 3.72E-04 | -1.569 |
| 271 |  | Germ cell differentiation signaling pathway | 3.72E-04 | 0.853 |
| 274 |  | Inhibition of ARE-mediated mRNA degradation pathway | 3.80E-04 | -2.746 |
| 273 |  | FOXO-mediated transcription of cell death genes | 3.80E-04 | -1.134 |
| 275 |  | Role of JAK family kinases in IL-6-type cytokine signaling | 4.07E-04 | -0.500 |
| 276 |  | Corticotropin releasing hormone signaling | 4.07E-04 | 0.229 |
| 278 |  | Xenobiotic metabolism general signaling pathway | 4.17E-04 | -0.408 |
| 277 |  | TNFR1 signaling | 4.17E-04 | 0.302 |
| 279 |  | NGF-stimulated transcription | 4.37E-04 | -2.111 |
| 280 |  | Cholesterol biosynthesis | 4.47E-04 | 3.000 |
| 282 |  | Sialic acid metabolism | 4.57E-04 | 1.897 |
| 284 |  | TNFR2 signaling | 4.57E-04 | -1.633 |
| 287 |  | BEX2 signaling pathway | 4.57E-04 | -1.604 |
| 285 |  | D-myo-inositol (1,4,5,6)-tetrakisphosphate biosynthesis | 4.57E-04 | -1.528 |
| 286 |  | D-myo-inositol (3,4,5,6)-tetrakisphosphate biosynthesis | 4.57E-04 | -1.528 |
| 283 | 45 | Airway inflammation in asthma | 4.57E-04 | 1.134 |
| 281 | 46 | IL-15 production | 4.57E-04 | 0.000 |
| 289 |  | DNA damage-induced 14-3-3σ signaling | 4.68E-04 | 2.111 |
| 288 |  | SPINK1 general cancer pathway | 4.68E-04 | 0.258 |
| 290 |  | Hematopoiesis from multipotent stem cells | 4.79E-04 | NaN |
| 291 |  | Semaphorin neuronal repulsive signaling pathway | 5.25E-04 | -0.408 |
| 292 |  | LPS/IL-1 mediated inhibition of RXR function | 5.50E-04 | -1.698 |
| 293 |  | Melanocyte development and pigmentation signaling | 5.50E-04 | 0.943 |
| 295 |  | cAMP-mediated signaling | 5.62E-04 | 1.061 |
| 294 |  | FOXO-mediated transcription of cell cycle genes | 5.62E-04 | -0.378 |
| 296 |  | Transcriptional regulation by E2F6 | 5.75E-04 | -2.530 |
| 297 |  | Fatty acid β-oxidation I | 5.75E-04 | 1.000 |
| 298 |  | Xenobiotic metabolism AHR signaling pathway | 6.03E-04 | -0.832 |
| 299 |  | GPCR-mediated integration of enteroendocrine signaling exemplified by an L cell | 6.76E-04 | 0.535 |
| 300 |  | ISGylation signaling pathway | 6.76E-04 | 0.000 |
| 303 | 47 | Agranulocyte adhesion and diapedesis | 6.92E-04 | NaN |
| 304 |  | Intrinsic pathway for apoptosis | 6.92E-04 | 1.941 |
| 301 |  | Sumoylation pathway | 6.92E-04 | -1.807 |
| 302 |  | Factors promoting cardiogenesis in vertebrates | 6.92E-04 | 0.426 |
| 305 |  | RANK signaling in osteoclasts | 6.92E-04 | -0.277 |
| 306 |  | RAF-independent MAPK1/3 activation | 7.08E-04 | 0.000 |
| 307 |  | Germ cell-sertoli cell junction signaling | 7.76E-04 | NaN |
| 310 |  | Platelet homeostasis | 7.76E-04 | 2.183 |
| 311 |  | VEGF family ligand-receptor interactions | 7.76E-04 | -0.500 |
| 308 |  | VDR/RXR activation | 7.76E-04 | -0.302 |
| 309 | 48 | IL-2 signaling | 7.76E-04 | 0.277 |
| 312 |  | Gastrin-CREB signaling pathway via PKC and MAPK | 7.94E-04 | -0.378 |
| 313 |  | Differential regulation of cytokine production in macrophages and T helper cells by IL-17A and IL-17F | 7.94E-04 | -0.378 |
| 315 | 49 | iNOS signaling | 8.32E-04 | -1.134 |
| 314 |  | Co-stimulation by CD28 | 8.32E-04 | -0.577 |
| 316 |  | Nitric oxide signaling in the cardiovascular system | 8.91E-04 | -0.229 |
| 317 |  | Transcriptional regulatory network in embryonic stem cells | 9.12E-04 | -0.784 |
| 318 |  | Thrombopoietin signaling | 9.12E-04 | -0.277 |
| 319 |  | ERB2-ERBB3 signaling | 9.12E-04 | 0.277 |
| 321 |  | Oncostatin M signaling | 9.77E-04 | 0.333 |
| 320 |  | Folate signaling pathway | 9.77E-04 | -0.277 |
| 322 |  | Signaling by TGFBR3 | 1.00E-03 | 0.577 |
| 323 |  | UBE2C in cell cycle progression signaling pathway | 1.00E-03 | -0.243 |
| 325 |  | Interleukin-1 family signaling | 1.02E-03 | -1.706 |
| 324 |  | Signaling by Rho family GTPases | 1.02E-03 | 1.177 |
| 326 |  | Nuclear cytoskeleton signaling pathway | 1.10E-03 | 1.616 |
| 327 |  | TGF-β signaling | 1.12E-03 | 0.000 |
| 328 |  | Cancer drug resistance by drug efflux | 1.15E-03 | 0.905 |
| 331 |  | Colanic acid building blocks biosynthesis | 1.17E-03 | 2.449 |
| 329 |  | Cilia biogenesis signaling pathway | 1.17E-03 | 1.826 |
| 330 |  | UVC-induced MAPK signaling | 1.17E-03 | 0.302 |
| 332 |  | L1CAM interactions | 1.20E-03 | 1.091 |
| 334 |  | Pyridoxal 5'-phosphate salvage pathway | 1.23E-03 | 1.604 |
| 333 | 50 | Activation of IRF by cytosolic pattern recognition receptors | 1.23E-03 | 0.000 |
| 336 |  | Orexin signaling pathway | 1.26E-03 | 1.414 |
| 335 |  | DNA methylation and transcriptional repression signaling | 1.26E-03 | 0.000 |
| 337 |  | IL-13 signaling pathway | 1.32E-03 | -0.894 |
| 338 |  | TWEAK signaling | 1.38E-03 | 1.414 |
| 340 |  | WNT/Ca2+ signaling pathway | 1.41E-03 | 0.302 |
| 339 |  | ERBB4 signaling | 1.41E-03 | -0.277 |
| 342 |  | Endometrial cancer signaling | 1.55E-03 | -0.577 |
| 343 |  | RIPK1-mediated regulated necrosis | 1.55E-03 | -0.333 |
| 341 |  | WNT/SHH axonal guidance signaling pathway | 1.55E-03 | 0.000 |
| 344 |  | Salvage pathways of pyrimidine ribonucleotides | 1.58E-03 | 1.698 |
| 345 |  | Coronavirus pathogenesis pathway | 1.62E-03 | -1.671 |
| 346 |  | Signaling by EGFR | 1.66E-03 | -1.155 |
| 347 |  | GDNF family ligand-receptor interactions | 1.66E-03 | 1.155 |
| 349 | 51 | Clathrin-mediated endocytosis signaling | 1.70E-03 | NaN |
| 348 |  | Glutaminergic receptor signaling pathway (enhanced) | 1.70E-03 | 0.949 |
| 350 |  | PAK signaling | 1.74E-03 | 0.728 |
| 351 |  | Aldosterone signaling in epithelial cells | 1.74E-03 | 0.229 |
| 353 |  | Protein sorting signaling pathway | 1.86E-03 | 1.800 |
| 355 | 52 | Toll-like receptor signaling | 1.86E-03 | -1.134 |
| 352 |  | α-adrenergic signaling | 1.86E-03 | 0.832 |
| 354 |  | TP53 regulates metabolic genes | 1.86E-03 | 0.000 |
| 356 |  | Regulation of lipid metabolism by PPARalpha | 1.91E-03 | -2.236 |
| 358 |  | MAPK targets/nuclear events mediated by MAP kinases | 1.91E-03 | 1.000 |
| 357 |  | Bladder cancer signaling | 1.91E-03 | 0.378 |
| 361 |  | Ferroptosis signaling pathway | 1.95E-03 | 1.964 |
| 363 |  | Zn homeostasis signaling pathway | 1.95E-03 | 1.854 |
| 362 |  | C-type lectin receptors (CLRs) | 1.95E-03 | -1.460 |
| 359 |  | Insulin receptor signaling | 1.95E-03 | -1.091 |
| 360 |  | Endocannabinoid developing neuron pathway | 1.95E-03 | 0.471 |
| 364 |  | Transcriptional regulation by VENTX | 2.00E-03 | 0.632 |
| 365 |  | Integrin signaling | 2.04E-03 | 1.414 |
| 367 |  | Clathrin-mediated endocytosis | 2.09E-03 | 0.655 |
| 366 |  | Leptin signaling in obesity | 2.09E-03 | -0.378 |
| 368 | 53 | Role of cytokines in mediating communication between immune cells | 2.24E-03 | NaN |
| 369 |  | Apelin adipocyte signaling pathway | 2.24E-03 | 0.577 |
| 370 |  | G Beta gamma signaling | 2.29E-03 | 0.688 |
| 373 | 54 | Primary immunodeficiency signaling | 2.34E-03 | NaN |
| 372 | 55 | 4-1BB signaling in T lymphocytes | 2.34E-03 | -1.342 |
| 374 |  | Signaling by MET | 2.34E-03 | 1.291 |
| 371 |  | Reelin signaling in neurons | 2.34E-03 | 0.218 |
| 376 |  | Mismatch repair | 2.45E-03 | 2.449 |
| 375 |  | Caspase activation via death receptors in the presence of ligand | 2.45E-03 | -1.633 |
| 377 |  | Co-stimulation by ICOS | 2.45E-03 | 0.447 |
| 378 |  | EGF signaling | 2.57E-03 | 0.905 |
| 379 |  | Cargo recognition for clathrin-mediated endocytosis | 2.63E-03 | 0.943 |
| 380 |  | Pancreatic secretion signaling pathway | 2.63E-03 | 0.354 |
| 382 |  | UFMylation signaling pathway | 2.69E-03 | 0.832 |
| 381 |  | Role of BRCA1 in DNA damage response | 2.69E-03 | 0.000 |
| 383 |  | TP53 regulates transcription of cell cycle genes | 2.75E-03 | 0.302 |
| 384 |  | Oncogene induced senescence | 2.82E-03 | 1.667 |
| 388 |  | Regulation of cellular mechanics by calpain protease | 2.88E-03 | 2.530 |
| 385 |  | April mediated signaling | 2.88E-03 | -1.890 |
| 386 | 56 | Antigen presentation pathway | 2.88E-03 | 1.633 |
| 387 |  | Paxillin signaling | 2.88E-03 | 1.604 |
| 389 |  | Dopamine-DARPP32 feedback in cAMP signaling | 2.95E-03 | 1.706 |
| 390 |  | Interferon gamma signaling | 3.02E-03 | 0.243 |
| 392 |  | SUMOylation of DNA methylation proteins | 3.39E-03 | -1.633 |
| 393 |  | Signaling by SCF-KIT | 3.39E-03 | -1.265 |
| 391 |  | ER-mitochondrial communication signaling pathway | 3.39E-03 | 0.535 |
| 394 |  | NFE2L2 regulating anti-oxidant/detoxification enzymes | 3.55E-03 | -2.646 |
| 398 |  | NER (nucleotide excision repair, enhanced pathway) | 3.55E-03 | 2.496 |
| 395 |  | Differential regulation of cytokine production in intestinal epithelial cells by IL-17A and IL-17F | 3.55E-03 | -1.134 |
| 396 |  | Amyotrophic lateral sclerosis signaling | 3.55E-03 | 1.000 |
| 397 |  | NRF2-mediated oxidative stress response | 3.55E-03 | -0.853 |
| 399 |  | TCR signaling | 3.89E-03 | -1.342 |
| 400 |  | Role of PI3K/AKT signaling in the pathogenesis of influenza | 3.98E-03 | -0.302 |
| 401 |  | Role of hypercytokinemia/hyperchemokinemia in the pathogenesis of influenza | 3.98E-03 | 0.000 |
| 403 | 57 | Phagosome maturation | 4.07E-03 | NaN |
| 402 |  | Regulation of CDH11 expression and function | 4.07E-03 | -0.707 |
| 404 |  | Detoxification of reactive oxygen species | 4.17E-03 | 1.000 |
| 406 |  | Intra-golgi and retrograde golgi-to-ER traffic | 4.37E-03 | 2.268 |
| 405 |  | Plasma lipoprotein assembly, remodeling, and clearance | 4.37E-03 | 1.604 |
| 407 |  | Regulation of TP53 activity through phosphorylation | 4.47E-03 | 3.000 |
| 410 | 58 | Inflammasome pathway | 4.47E-03 | -0.447 |
| 408 |  | Interleukin-6 family signaling | 4.47E-03 | 0.378 |
| 409 |  | Regulation of RUNX1 expression and activity | 4.47E-03 | 0.378 |
| 411 |  | Vitamin K signaling pathway | 4.47E-03 | 0.277 |
| 412 | 59 | CCR3 signaling in eosinophils | 4.57E-03 | 0.535 |
| 413 |  | N-acetylglucosamine degradation II | 4.79E-03 | NaN |
| 414 |  | Antiproliferative role of somatostatin receptor 2 | 4.90E-03 | 0.577 |
| 415 |  | Signaling by BMP | 4.90E-03 | 0.000 |
| 417 |  | FGF signaling | 5.25E-03 | 0.535 |
| 416 |  | Huntington's disease signaling | 5.25E-03 | -0.392 |
| 418 |  | Neddylation | 5.37E-03 | 0.707 |
| 419 |  | Neurotrophin/TRK signaling | 5.50E-03 | 0.832 |
| 420 |  | Apelin pancreas signaling pathway | 5.50E-03 | 0.707 |
| 421 |  | FLT3 signaling | 5.89E-03 | -1.667 |
| 422 |  | Toll-like receptor cascades | 6.03E-03 | 0.000 |
| 423 |  | γ-linolenate biosynthesis II (animals) | 6.03E-03 | 0.000 |
| 424 |  | Mitotic G1 phase and G1/S transition | 6.46E-03 | 0.943 |
| 426 |  | NCAM signaling for neurite out-growth | 6.61E-03 | 1.732 |
| 428 |  | Signaling by PTK6 | 6.61E-03 | 0.905 |
| 427 |  | Transcriptional regulation by MECP2 | 6.61E-03 | 0.577 |
| 425 |  | VEGF signaling | 6.61E-03 | 0.000 |
| 429 |  | CDK5 signaling | 6.76E-03 | 0.832 |
| 430 |  | IL-17A signaling in gastric cells | 7.08E-03 | 0.000 |
| 432 |  | XBP1(S) activates chaperone genes | 7.41E-03 | 3.162 |
| 433 |  | DNA damage bypass | 7.41E-03 | 2.530 |
| 431 |  | IL-1 signaling | 7.41E-03 | -1.414 |
| 434 |  | Inositol phosphate metabolism | 7.41E-03 | -0.632 |
| 436 | 60 | CD27 signaling in lymphocytes | 7.59E-03 | -2.121 |
| 435 |  | CSDE1 signaling pathway | 7.59E-03 | 1.000 |
| 437 |  | Iron homeostasis signaling pathway | 7.94E-03 | NaN |
| 438 |  | Factors involved in megakaryocyte development and platelet production | 7.94E-03 | 0.243 |
| 439 |  | Signaling by the B cell receptor (BCR) | 7.94E-03 | 0.000 |
| 440 |  | Atherosclerosis signaling | 8.13E-03 | -0.728 |
| 441 |  | Interleukin-1 processing | 8.32E-03 | -1.000 |
| 444 |  | Role of JAK1, JAK2 and TYK2 in interferon signaling | 8.71E-03 | NaN |
| 442 |  | TNF signaling | 8.71E-03 | -1.508 |
| 445 |  | Lysosome positioning signaling pathway | 8.71E-03 | -0.426 |
| 443 |  | Effects of PIP2 hydrolysis | 8.71E-03 | 0.378 |
| 446 |  | BBSome signaling pathway | 9.33E-03 | 2.598 |
| 447 |  | Chemokine signaling | 9.33E-03 | 1.155 |
| 449 |  | Activation of gene expression by SREBF (SREBP) | 9.55E-03 | 1.000 |
| 448 |  | MITF-M-dependent gene expression | 9.55E-03 | -0.728 |
| 450 |  | nNOS signaling in neurons | 9.77E-03 | NaN |
| 451 |  | Extra-nuclear estrogen signaling | 1.00E-02 | -0.277 |
| 456 |  | FasL/CD95L signaling | 1.02E-02 | NaN |
| 457 |  | CMP-N-acetylneuraminate biosynthesis I (eukaryotes) | 1.02E-02 | NaN |
| 458 |  | Citrulline-nitric oxide cycle | 1.02E-02 | NaN |
| 459 |  | Galactose degradation I (leloir pathway) | 1.02E-02 | NaN |
| 461 |  | Transcriptional regulation of white adipocyte differentiation | 1.02E-02 | -1.069 |
| 453 |  | GPCR-mediated nutrient Sensing in enteroendocrine cells | 1.02E-02 | 1.000 |
| 454 |  | GPVI-mediated activation cascade | 1.02E-02 | -0.707 |
| 455 |  | Transcriptional regulation by NPAS4 | 1.02E-02 | 0.707 |
| 452 |  | Histone modification signaling pathway | 1.02E-02 | -0.169 |
| 460 |  | Leukotriene biosynthesis | 1.02E-02 | 0.000 |
| 462 |  | Transport of vitamins, nucleosides, and related molecules | 1.10E-02 | 0.333 |
| 463 |  | Transcriptional activity of SMAD2/SMAD3:SMAD4 heterotrimer | 1.12E-02 | -1.667 |
| 464 |  | Endocannabinoid neuronal synapse pathway | 1.23E-02 | 2.183 |
| 465 |  | Cardiac hypertrophy signaling | 1.23E-02 | 1.732 |
| 467 |  | Response of EIF2AK1 (HRI) to heme deficiency | 1.26E-02 | -1.890 |
| 466 |  | Peroxisomal lipid metabolism | 1.26E-02 | 1.890 |
| 468 |  | TP53 regulates transcription of cell death genes | 1.26E-02 | 1.667 |
| 469 |  | TBC/RABGAPs | 1.26E-02 | 1.667 |
| 470 |  | Cell cycle: G1/S checkpoint regulation | 1.32E-02 | -1.155 |
| 471 |  | Necroptosis signaling pathway | 1.32E-02 | -1.091 |
| 472 |  | Glutaryl-CoA degradation | 1.35E-02 | 2.000 |
| 473 |  | Signaling by NOTCH1 | 1.35E-02 | -0.277 |
| 474 |  | Class I MHC mediated antigen processing and presentation | 1.38E-02 | -0.762 |
| 475 |  | Sleep NREM signaling pathway | 1.41E-02 | -1.000 |
| 476 |  | Sulfate activation for sulfonation | 1.48E-02 | NaN |
| 479 |  | Tryptophan degradation III (eukaryotic) | 1.51E-02 | 2.236 |
| 478 |  | Signaling by NTRK3 (TRKC) | 1.51E-02 | 1.134 |
| 477 |  | Role of MAPK signaling in inhibiting the pathogenesis of influenza | 1.51E-02 | -0.832 |
| 480 |  | Kinesins | 1.55E-02 | 2.714 |
| 481 |  | Androgen signaling | 1.58E-02 | 0.832 |
| 482 |  | Netrin signaling | 1.58E-02 | 0.209 |
| 483 |  | Mitochondrial protein degradation | 1.62E-02 | 2.840 |
| 485 |  | Resolution of abasic sites (AP sites) | 1.62E-02 | 2.121 |
| 488 |  | Inhibition of matrix metalloproteases | 1.62E-02 | -1.890 |
| 484 |  | MyD88-independent TLR4 cascade | 1.62E-02 | -1.414 |
| 486 |  | Cell cycle regulation by BTG family proteins | 1.62E-02 | 1.342 |
| 487 | 61 | MIF-mediated glucocorticoid regulation | 1.62E-02 | 0.000 |
| 489 |  | Gαi signaling | 1.66E-02 | 1.000 |
| 490 |  | WNK renal signaling pathway | 1.70E-02 | -1.387 |
| 491 |  | Cell surface interactions at the vascular wall | 1.70E-02 | 0.192 |
| 494 |  | Galactose catabolism | 1.78E-02 | NaN |
| 495 |  | Arginine biosynthesis IV | 1.78E-02 | NaN |
| 496 |  | Zymosterol biosynthesis | 1.78E-02 | NaN |
| 497 |  | Degradation of the extracellular matrix | 1.78E-02 | 1.941 |
| 492 |  | Signaling by ALK | 1.78E-02 | 0.378 |
| 493 |  | MicroRNA biogenesis signaling pathway | 1.78E-02 | 0.209 |
| 499 |  | Vitamin-C transport | 1.82E-02 | -0.447 |
| 498 |  | RHO GTPases activate NADPH oxidases | 1.82E-02 | 0.000 |
| 500 |  | MAP kinase activation | 1.86E-02 | -2.828 |
| 502 |  | Cell cycle checkpoints | 1.91E-02 | 4.116 |
| 501 |  | mTOR signaling | 1.91E-02 | -0.408 |
| 505 |  | Synaptic long term depression | 1.95E-02 | 1.460 |
| 504 |  | ROBO SLIT signaling pathway | 1.95E-02 | 0.728 |
| 503 |  | Dermatan sulfate biosynthesis | 1.95E-02 | -0.632 |
| 506 |  | NLR signaling pathways | 2.04E-02 | -1.265 |
| 507 |  | Turbulent (oscillatory, disturbed) flow shear stress activates signaling by PIEZO1 and integrins in endothelial cells | 2.09E-02 | -0.378 |
| 508 |  | Isoleucine degradation I | 2.19E-02 | NaN |
| 511 |  | COPI-mediated anterograde transport | 2.24E-02 | 3.873 |
| 509 |  | Mitochondrial RNA degradation | 2.24E-02 | 1.633 |
| 510 |  | AMPK signaling | 2.24E-02 | 0.943 |
| 512 |  | eNOS signaling | 2.29E-02 | -1.000 |
| 513 |  | HDR through homologous recombination (HRR) or single strand annealing (SSA) | 2.40E-02 | 3.771 |
| 514 |  | Inhibition of angiogenesis by TSP1 | 2.45E-02 | -2.000 |
| 515 |  | Class A/1 (rhodopsin-like receptors) | 2.57E-02 | 1.808 |
| 516 |  | Integrin cell surface interactions | 2.57E-02 | 0.832 |
| 517 |  | Ribavirin ADME | 2.57E-02 | -0.302 |
| 518 |  | FAK signaling | 2.63E-02 | 1.744 |
| 519 |  | Signaling by erythropoietin | 2.63E-02 | -1.633 |
| 520 |  | Cardiomyocyte differentiation via BMP receptors | 2.63E-02 | 0.816 |
| 521 |  | Mitochondrial L-carnitine shuttle pathway | 2.69E-02 | -0.447 |
| 523 |  | Aryl hydrocarbon receptor signaling | 2.75E-02 | NaN |
| 524 |  | TYSND1 cleaves peroxisomal proteins | 2.75E-02 | NaN |
| 525 |  | Regulation of CDH19 expression and function | 2.75E-02 | NaN |
| 522 |  | G alpha (i) signaling events | 2.75E-02 | 2.556 |
| 527 |  | Agrin interactions at neuromuscular junction | 2.82E-02 | 1.890 |
| 526 |  | Polyamine regulation in colon cancer | 2.82E-02 | -1.000 |
| 528 |  | G alpha (s) signaling events | 2.95E-02 | 1.606 |
| 529 |  | Oleate biosynthesis II (animals) | 3.09E-02 | NaN |
| 530 | 62 | Caveolar-mediated endocytosis signaling | 3.09E-02 | 1.134 |
| 532 |  | Syndecan interactions | 3.09E-02 | 0.816 |
| 531 |  | Cytoprotection by HMOX1 | 3.09E-02 | -0.632 |
| 533 |  | DAP12 interactions | 3.16E-02 | 0.000 |
| 534 |  | Late endosomal microautophagy | 3.24E-02 | 1.890 |
| 535 |  | Interleukin-12 family signaling | 3.31E-02 | -0.447 |
| 536 |  | Retinoic acid mediated apoptosis signaling | 3.47E-02 | 1.265 |
| 537 |  | p75 NTR receptor-mediated signaling | 3.47E-02 | -0.447 |
| 538 |  | N-acetylglucosamine degradation I | 3.72E-02 | NaN |
| 539 | 63 | MSP-RON signaling pathway | 3.80E-02 | NaN |
| 541 |  | Opioid signaling | 3.89E-02 | 2.496 |
| 540 |  | Actin cytoskeleton signaling | 3.89E-02 | 0.688 |
| 542 |  | DNA double-strand break repair by homologous recombination | 3.98E-02 | NaN |
| 543 |  | Tight junction signaling | 3.98E-02 | NaN |
| 547 |  | TRAIL signaling | 3.98E-02 | NaN |
| 548 |  | Regulation of NFE2L2 gene expression | 3.98E-02 | NaN |
| 549 |  | NFE2L2 regulating MDR associated enzymes | 3.98E-02 | NaN |
| 550 |  | Sucrose degradation V (mammalian) | 3.98E-02 | NaN |
| 551 |  | γ-glutamyl cycle | 3.98E-02 | NaN |
| 544 |  | Branched-chain amino acid catabolism | 3.98E-02 | 1.342 |
| 545 |  | Synaptic adhesion-like molecules | 3.98E-02 | 1.342 |
| 546 |  | Co-inhibition by CTLA4 | 3.98E-02 | -0.447 |
| 552 |  | Neuropeptides in migraine signaling pathway | 3.98E-02 | 0.447 |
| 553 |  | Dilated cardiomyopathy signaling pathway | 4.17E-02 | 0.832 |
| 554 |  | PTEN regulation | 4.17E-02 | 0.229 |
| 555 |  | Myogenesis | 4.27E-02 | -0.816 |
| 557 |  | WNT/β-catenin signaling | 4.47E-02 | 0.243 |
| 556 | 64 | MIF regulation of innate immunity | 4.47E-02 | 0.000 |
| 558 |  | Semaphorin interactions | 4.57E-02 | 0.632 |
| 559 |  | Chaperone mediated autophagy | 4.68E-02 | 2.236 |
| 560 |  | Pyrimidine deoxyribonucleotides de novo biosynthesis I | 4.68E-02 | 2.000 |
| 562 |  | Signaling by CSF3 (G-CSF) | 4.90E-02 | -1.633 |
| 561 |  | Fatty acid activation | 4.90E-02 | -1.000 |

Table 4: Number of identified H3Ac peaks at each CD8^+^ T cell differentiation stage for each replicate independently including number of MACS called peaks, number of DiffBind consensus peaks, number of DiffBind consensus peaks within ±1,000 bp of the transcription start site (TSS), and number of DiffBind consensus peaks within ±1,000 bp of differentially-expressed genes (DEGs).

|  | **Replicate** | **No. of MACS called peaks** | **No. of Diffbind consensus peaks**  **(total n=11,622)** | **No. of Diffbind consensus peaks**  **within 1 kb of TSS**  **(total n=8,489)** | **No. of Diffbind consensus peaks**  **within 1 kb of TSS of DEGs**  **(total n=4,176)** |
| --- | --- | --- | --- | --- | --- |
| IL-2 | IL2_rep1 | 14,749 | 8,883 | 6,882 | 3,365 |
|  | IL2_rep2 | 2,231 | 1,096 | 867 | 411 |
|  | IL2_rep3 | 4,045 | 1,958 | 1,719 | 751 |
| Tc1 | Tc1_rep1 | 13,169 | 8,160 | 6,270 | 2,988 |
|  | Tc1_rep2 | 11,452 | 7,193 | 6,089 | 2,904 |
|  | Tc1_rep3 | 14,146 | 7,260 | 6,003 | 2,851 |
| IL-2/IL-4 | IL-2/IL-4_rep1 | 16,696 | 8,925 | 6,978 | 3,357 |
|  | IL-2/IL-4_rep2 | 6,875 | 4,186 | 3,520 | 1,629 |
|  | IL-2/IL-4_rep3 | 2,252 | 832 | 714 | 287 |
| Tc2 | Tc2_rep1 | 12,393 | 7,337 | 5,825 | 2,750 |
|  | Tc2_rep2 | 8,311 | 4,701 | 3,648 | 1,735 |
|  | Tc2rep3 | 13,336 | 8,283 | 6,656 | 3,152 |
